# Metagenomic Insight into Lignocellulose Degradation of the Thermophilic Microbial Consortium TMC7

**DOI:** 10.4014/jmb.2106.06015

**Published:** 2021-06-29

**Authors:** Yi Wang, Chen Wang, Yonglun Chen, Beibei Chen, Peng Guo, Zongjun Cui

**Affiliations:** 1 Institute of Agricultural Products Processing and Nuclear Agriculture Technology Research, Hubei Academy of Agricultural Sciences, Wuhan 430064, P.R. China; 2 College of Biology and Pharmacy, Three Gorges University, Yichang 443002, P.R. China; 3 College of Agronomy and Biotechnology, China Agricultural University, Beijing 100193, P.R. China

**Keywords:** Llignocellulose degradation, microbial consortium, metagenomics, CAZymes, sugar cheater

## Abstract

Biodegradation is the key process involved in natural lignocellulose biotransformation and utilization. Microbial consortia represent promising candidates for applications in lignocellulose conversion strategies for biofuel production; however, cooperation among the enzymes and the labor division of microbes in the microbial consortia remains unclear. In this study, metagenomic analysis was performed to reveal the community structure and extremozyme systems of a lignocellulolytic microbial consortium, TMC7. The taxonomic affiliation of TMC7 metagenome included members of the genera *Ruminiclostridium* (42.85%), *Thermoanaerobacterium* (18.41%), *Geobacillus* (10.44%), unclassified_f__Bacillaceae (7.48%), *Aeribacillus* (2.65%), *Symbiobacterium* (2.47%), *Desulfotomaculum* (2.33%), *Caldibacillus* (1.56%), *Clostridium* (1.26%), and others (10.55%). The carbohydrate-active enzyme annotation revealed that TMC7 encoded a broad array of enzymes responsible for cellulose and hemicellulose degradation. Ten glycoside hydrolases (GHs) endoglucanase, 4 GHs exoglucanase, and 6 GHs β-glucosidase were identified for cellulose degradation; 6 GHs endo-β-1,4-xylanase, 9 GHs β-xylosidase, and 3 GHs β-mannanase were identified for degradation of the hemicellulose main chain; 6 GHs arabinofuranosidase, 2 GHs α-mannosidase, 11 GHs galactosidase, 3 GHs α-rhamnosidase, and 4 GHs α-fucosidase were identified as xylan debranching enzymes. Furthermore, by introducing a factor named as the contribution coefficient, we found that *Ruminiclostridium* and *Thermoanaerobacterium* may be the dominant contributors, whereas *Symbiobacterium* and *Desulfotomaculum* may serve as “sugar cheaters” in lignocellulose degradation by TMC7. Our findings provide mechanistic profiles of an array of enzymes that degrade complex lignocellulosic biomass in the microbial consortium TMC7 and provide a promising approach for studying the potential contribution of microbes in microbial consortia.

## Introduction

The development of alternative energy sources to replace fossil fuels is an urgent global priority. Cellulosic biomass, also referred to as lignocellulosic biomass, is considered as the most scalable alternative fuel source [1]. As the skeleton and protective barriers of plant cells, lignocellulose has a recalcitrant structure consisting of three main components: cellulose, hemicellulose, and lignin [2]. The degradation of these polymers requires the synergetic action of complex enzymes, with pure-culture microorganisms showing lower degradation efficiency. In nature, lignocellulose is decomposed mainly by complex lignocellulolytic microbial communities [3]. Inspired by this, enrichment of microbial communities provides good strategies for the biotransformation of lignocellulose as well for studies of the interactions among their microbial members and lignocellulolytic enzymes.

In our previous work, a thermophilic lignocellulolytic microbial consortium TMC7 with high extracellular xylanase activity was enriched from compost habitats [4]. The resulting consortium exhibited effectively degraded both alkali-treated and natural lignocellulosic materials, showing 77.4% and 42.2% weight loss, respectively, over 12 days of incubation at 65°C. The extracellular xylanase activity was greater than 80% and 85%over a wide range of temperatures (50–75°C) and pH values (6.0–11.0), respectively. Phylogenetic analysis of TMC7 using 16S rRNA gene sequencing showed that the constituent bacteria were mainly related to the genera *Clostridium*, *Geobacillus*, *Aeribacillus*, and *Thermoanaerobacterium*. These preliminary findings indicate the potential practical application of TMC7 for lignocellulosic biomass utilization under thermophilic conditions in the biotechnology industry. However, the inventory of valuable thermophilic carbohydrate-active proteins in TMC7 that deconstruct lignocellulose and the interactions of abundant microbial populations involved in this process are not completely understood.

Culture-independent metagenomic approaches are powerful approaches for revealing the lignocellulolytic potential of microbial consortia and substantially expanding the repertoire of genes involved in lignocellulose decomposition [5-7]. The “Carbohydrate-Active Enzyme database” (CAZy, http://www.cazy.org) [8] is an essential resource for studying the process of carbohydrate metabolism. Active enzymes related to the breakdown of carbohydrates are classified into six groups: glycoside hydrolase (GH), glycosyl transferase (GT), auxiliary activity (AA), carbohydrate esterase (CE), polysaccharide lyases (PL), and carbohydrate-binding domains (CBM). The cooperation of multiple CAZymes in lignocellulose degradation was studied in microbial consortia originating from compost [4], woodlice gut [9], forest [10], and biogas-producing digesters [11].

In further, although omics studies may identify CAZymes from microbial consortia enrichment, it is still hard to calculate the functional efficiency of the consortia. According to Jiménez *et al*. [12], in lignocellulolytic microbial consortia microbes occupy different niches defined by the carbon (energy) sources in the culture. Meanwhile, some opportunistic organisms can be selected throughout the enrichment cultures. These “sugar cheaters” consume the public goods produced by other community members, but contribute little in lignocellulose degradation process. The Black Queen Hypothesis explains how public goods dynamics drive the origin of dependencies over an evolutionary timescale, predicting that when an individual loses a costly, leaky function, it will receive a selective advantage and expand in community until the production of public goods is just sufficient to support the equilibrium community [13]. However, high levels of “sugar cheaters” will result demand for the resource overwhelming the supply and decrease the productivity of communities. Thus, calculation of the contribution of the microbes is important for studying the roles of the community members and evaluating the functional efficiency of the microbial consortia.

Compared with natural microbial consortia, a stable microbial consortium has eliminated the useless microbes and got a highly specialized microbial composition during the long-term domestication process with lignocellulose substrate [12]. Thus, study of the stable microbial consortium could exclude the interference of useless microbes in the lignocellulolytic microbial consortia. In this study, a metagenomic approach was used to investigate the thermophilic lignocellulolytic microbial consortium TMC7. CAZyme annotation and analysis were performed to provide a comprehensive framework for the decomposition of the main components of lignocellulose, namely cellulose, hemicellulose, and lignin. On these basis, the taxonomic affiliation of these CAZymes was analyzed to determine the contribution of specific microbes and their potential roles during lignocellulose degradation. This study provides valuable insight into the mechanism of lignocellulose degradation by the TMC7 consortium and a promising approach for studying the contribution of microbes in microbial consortia.

## Materials and Methods

### Lignocellulosic Materials and Microbial Consortium Cultures

Lignocellulosic material preparation and microbial consortium cultivation were performed as described previously [4]. Briefly, corn stalks obtained locally from Wuhan, China were air-dried and alkali-treated before use. The relative contents of cellulose, hemicellulose, and lignin were respectively 37.1%, 24.1%, and 12.1% in air-dried corn stalk and 62.4%, 17.6%, and 5.7% in alkali-treated corn stalk. The microbial consortium TMC7 was enriched from thermophilic compost using a reduplicative subcultivation process [4] and preserved in 20%glycerol (v/v) at -80°C. TMC7 was activated and cultured in 350 ml of sterilized PCS medium (1 g/l peptone, 2 g/l yeast extract, 2 g/l CaCO_3_, 5 g/l NaCl, 0.35 g/l MgSO_4_•7H_2_O, and 1 g/l K_2_HPO_4_) supplemented with 1% (w/v) alkali-treated corn stalk. The cultures were incubated in a 500 mL flask with a loose aluminum cap under static conditions in the dark at 65°C for 7 days. The weight loss of corn stalk and lignocellulosic components was determined according to a gravimetrical method with uninoculated medium serving as a control [14]. The components of the cellulosic substrates were analyzed on a Ankom220 fiber analysator (Ankom, USA) by Van Soest [15] detergent fiber analysis.

### Enzyme Assays

The activities of lignocellulolytic enzymes in the culture supernatants were analyzed at 65°C. For crude enzyme preparation, 5 ml culture sample was centrifuged at 12,000 ×*g* for 10 min, and the supernatants were filtered through a 0.22 μm filter (Merck Millipore, USA) for use as the extracellular enzyme sample. Endoglucanase and xylanase activities were determined by the dinitrosalicylic acid method using low-viscosity carboxymethylcellulose and beechwood xylan as substrates, respectively.

The activities of cellobiohydrolase, β-glucosidase, β-xylosidase, β-galactosidase, and β-mannosidase were assayed using the respective nitrophenyl chromogenic substrates 4-nitrophenyl β-D-cellobioside, 4-nitrophenyl β-D-glucopyranoside, 4-nitrophenyl β-D-xylopyranoside, 4-nitrophenyl β-D-galactopyranoside, and 4-nitrophenyl β-D-mannopyranoside (Sigma-Aldrich, USA) according to the manufacturer’s instructions. One unit of enzyme activity was defined as the amount of enzyme that released 1 μmol of 4-nitrophenol (measured as optical density at 420 nm) in 1 min from the substrate.

### DNA Extraction and Metagenomic Sequencing

After centrifugation at 10,000 ×*g* for 10 min at 4°C, the cell pellets were collected, and total DNA was extracted using the E.Z.N.A Soil DNA kit (Omega BioTek, USA) according to the manufacturer’s instructions. DNA quantity and purity were detected using a NanoDrop 2000 microspectrophotometer (Thermo Fisher Scientific, USA), and DNA integrity was assessed using 1% agarose gels. Approximately 200 ng of total DNA was used to prepare a 300 bp DNA library using the TruSeq DNA Sample Prep Kit (Illumina, USA). DNA sequencing was performed on an Illumina HiSeq 2500 platform by Majorbio (China). Low-quality reads and sequencing adaptors were removed using Seqpreq (https://github.com/jstjohn/SeqPrep). Trimmed reads shorter than 50 bp were removed. After quality filtering and dereplication of the raw reads, *de novo* assembly was performed using IDBA-UD (http://i.cs.hku.hk/~alse/hkubrg/projects/idba_ud/). Assembled contigs longer than 300 bp were subjected to gene prediction using MetaGene (http://metagene.cb.k.u-tokyo.ac.jp/) [16]. A non-redundant gene catalog was constructed using CD-HIT (http://www.bioinformatics.org/cd-hit/) with 95% identity and a 90% coverage threshold [17]. Clean reads were aligned to the non-redundant gene catalog with 95% identity, and the read number of each predicted gene was counted using SOAPaligner (Version 2.22, http://soap.genomics.org.cn/) [18].

### Taxonomic and Carbohydrate-Active Enzyme Annotation

Taxonomic annotation of the predicted genes was performed by BLASTN searching against the NCBI NT database, with an e-value threshold of 1e-5 [19]. The relative abundances of the annotated microbes (R_g_) in the microbial consortium were calculated according to reads numbers as R_g_ = N_g_/N, in which N_g_ was the read number of a specific microbe and N was the total clean read number in the TMC7 metagenome. As the degradation of lignocellulose is closely related to carbohydrate-active enzymes, genes encoding carbohydrate-active enzymes were identified using the CAZy database [20]. The signature domains for every CAZy family were annotated using dbCAN based on hidden Markov models with an e-value threshold of 1e-5 [21]. Taxonomic affiliation of the annotated CAZymes was visualized using Circos (http://mkweb.bcgsc.ca/tableviewer/visualize/) [22].

As a division of labor occurs in a microbial consortium and opportunistic organisms are prevalent [12], functional diversity may be inconsistent with the relative taxonomic diversity. We defined the contribution coefficient (CC) to calculate the contribution of a specific genus to a specific function. First, one or several CAZymes involved in the target function were selected, and the reads numbers of these CAZymes were summed as the total read number of the target function N_f_. Next, the read numbers of these CAZymes annotated as the target genus were summed as N_g-f_, and the abundance of a specific genus with a specific function (R_g-f_ ) was calculated as R_g-f_ = N_g-f_/N_f_. Finally, the CC of the specific genus to the specific function was calculated as: CC = R_g-f_/R_g_.

### Statistical Analysis

All reported values are the average of triplicate evaluation (mean ± SD). The raw data of the TMC7 metagenomic sequence reads were deposited into the NCBI Sequence Read Archive database under the BioProject accession number PRJNA699959 (SRR13645124).

## Results and Discussion

### Lignocellulolytic Enzyme Activities of TMC7

**Fig. 1:**
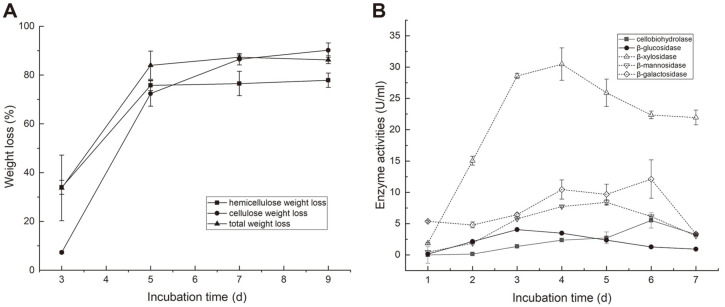
Lignocellulolytic activities of TMC7. (**A**) Weight loss of alkali-treated corn stalk, hemicellulose, and cellulose; (**B**) Activities of cellobiohydrolase, β-glucosidase, β-xylosidase, β-galactosidase, and β-mannosidase. Data are shown as the mean ± SD value of triplicate experiments.

Biotransformation and enzyme activity dynamics were detected to identify a suitable time for metagenomic analysis. The weight loss shown in Fig. 1A demonstrates that degradation of cellulose and hemicellulose mainly occurred during days 3–5. For cellulose degradation, the maximum cellobiohydrolase level of 5.51 U/ml was detected on day 6, and the maximum β-glucosidase level was 4.05 U/ml on day 3 (Fig. 1B). For hemicellulose degradation, the maximum β-xylosidase, β-mannosidase, and β-galactosidase levels were 30.47 U/ml on day 4, 8.40 U/ml on day 5, and 12.10 U/ml on day 6, respectively (Fig. 1B). These results indicate that TMC7 contains diverse glycoside hydrolases that access different structural components of lignocellulose, with degradation activity concentrated on days 3–6. Accordingly, 3-, 4-, 5-, and 6-day TMC7 cultures were sampled and pooled for DNA extraction and metagenomics sequencing.

### Overview of TMC7 Metagenomic Assembly

**Fig. 2:**
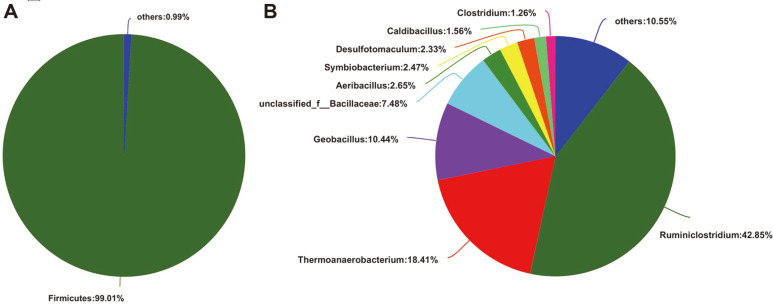
Microbial composition of TMC7 at the phylum (A) and genus (B) levels. The genera with abundance > 1% were present, and genera with abundance < 1% were combined as others.

After quality filtration and *de novo* assembly, 8,641 contigs longer than 300 bp were obtained with 83,777,510 total clean reads, with a 35,005 bp N50 length, 2,068 bp N90 length, and 375,887 bp maximum length. The metagenome of TMC7 was predicted to contain 47,010 open reading frames. Taxonomic analysis of all protein-coding genes in the TMC7 metagenome showed that over 99% of the phyla in TMC7 were assigned to *Firmicutes*. The TMC7 metagenome assembly showed a rather small number of assembled contigs compared to the reported lignocellulolytic microbial consortia metagenome [3, 23, 24], and the predominance of a single phylum was found only in TMC7 (Fig. 2A, Table S1). This indicates that TMC7 has a highly specialized and stable microbial composition during its long-term domestication process. Additionally, the thick peptidoglycan cell wall of *Firmicutes* can help the microbes resist ambient high temperatures.

At the genus level, the taxon of the predicted protein-encoding genes was dominated by nine genera with an abundance greater than 1%: 42.85% *Ruminiclostridium*, 18.41% *Thermoanaerobacterium*, 10.44% *Geobacillus*, 7.48% unclassified_f__Bacillaceae, 2.65% *Aeribacillus*, 2.47% *Symbiobacterium*, 2.33% *Desulfotomaculum*, 1.56%*Caldibacillus*, and 1.26% *Clostridium* (Fig. 2B, Table S2). Our results showed that TMC7 contained a complex of aerobic and anaerobic bacteria in submerged fermentation under static culture conditions. We hypothesized that the aerobic bacteria *Geobacillus* and Unclassified_f__Bacillaceae consume dissolved oxygen and create a suitable environment for the anaerobic bacteria *Ruminiclostridium* and *Thermoanaerobacterium*. Moreover, the coexisting aerobic Bacillus and anaerobic *Clostridium* in the microbial consortium shows positive synergistic interactions for extracellular xylanase secretion and lignocellulose degradation [25].

### Lignocellulose-Degrading CAZymes in TMC7 Metagenome

**Fig. 3:**
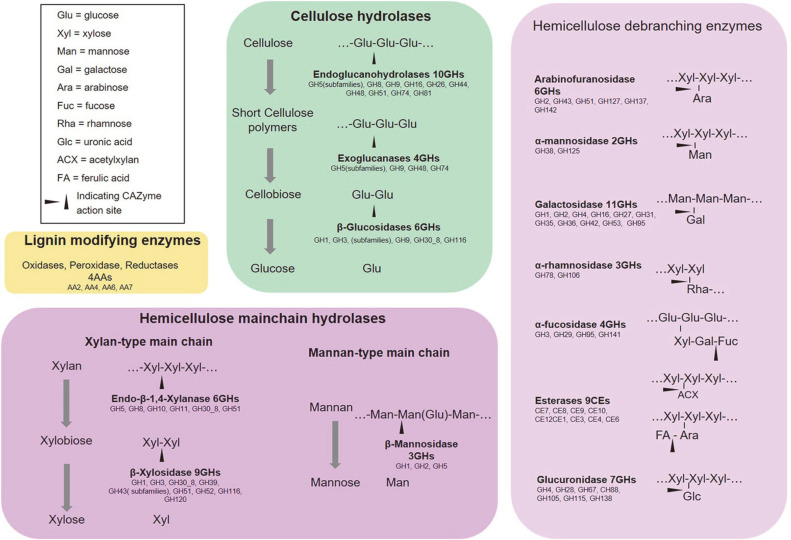
Mechanism profiles of lignocellulose degradation in TMC7. (I) Lignin (orange): TMC7 exhibited little lignin degradation activity with only 4 AAs; (II) Cellulose (green): Full enzymatic hydrolysis of cellulose requires the cooperation of endoglucanase (10 GHs), exoglucanase (4 GHs), and β-glucosidase (6 GHs); (III) Hemicellulose main chain (deep purple): Degradation of the hemicellulose linear β-1,4-linked main chain requires the action of endo-β-1,4-xylanase (6 GHs) and β- xylosidase (9 GHs) for β-1,4-xylan, and β-mannanase (3 GHs) for β-1,4-mannan; (IV) Debranching enzymes (light purple): Debranching enzymes for degradation of complex substituted xylans included 6 GHs arabinofuranosidase, 2 GHs α- mannosidase, 11 GHs galactosidase, 3 GHs α-rhamnosidase, 4 GHs α-fucosidase. Debranching enzymes for pectin included 7 GHs glucuronidase and 9 CEs esterase.

A total of 1658 putative CAZyme-encoding genes was identified in the metagenome of TMC7, with a read number of 4,868,962, accounting for 5.81% of the total reads in the TMC7 metagenome. The CAZyme repertoire of TMC7 contained 57 AA genes from four different families, 227 CBM genes from 27 families, 250 CE genes from 11 families, 643 GH genes from 102 families, 445 GT genes from 26 families, and 36 PL genes from 14 families (Table 1, Table S3). The selected CAZymes related to the degradation of lignocellulose components (cellulose, hemicellulose, pectin, and lignin) were examined in detail, with the 711 identified CAZyme-encoding genes listed in Table 2. Notably, many of these enzymes are multifunctional, each composed of two or more catalytic modules involved in degrading plant cell wall components. These CAZymes are summarized along with their mechanism profiles of lignocellulose degradation in Fig. 3. It was shown that TMC7 harbored almost full set of enzymes for cellulose and hemicellulose degradation.

As most lignin was removed during the alkali-pretreatment process, TMC7 exhibited little lignin degradation activity. Accordingly, only four AA families known to be lignin-modifying enzymes were found in TMC7. Among them, AA4 vanillyl-alcohol oxidase and AA6 benzoquinone reductase were dominant.

Cellulose is the main structural component of the plant cell wall [26]. Full enzymatic hydrolysis of cellulose requires the cooperation of endoglucanase, cellobiohydrolase (exoglucanase), and β-glucosidase [27, 28]. These functions were observed among the TMC7 CAZymes, with 13 GH families exhibiting cellulase activity identified. Among them, 10 endoglucanase GH families, i.e., GH5's subfamilies, GH8, GH9, GH16, GH26, GH44, GH48, GH51, GH74, and GH81 were identified. Four exoglucanase GH families, including GH5 subfamilies, GH9, GH48, and GH74 were identified. Six β-glucosidase GH families, including GH1, GH3, GH5 subfamilies, GH9, GH30_8, and GH116 were identified.

Hemicellulose and pectin link cellulose fibers into microfibrils and create a complex network of bonds that provide structural strength [26]. During the decomposition of lignocellulose, hemicellulose and pectin must be removed to increase the accessibility to cellulose. Based on the structure of highly heterogeneous polysaccharides [29, 30], hydrolysis of hemicellulose and pectin requires both linear β-1,4-linked chains of xylose and various debranching enzymes [28]. Corresponding to the high hemicellulose degradation activity, we found that the TMC7 metagenome harbored diverse CAZymes related to hemicellulose and pectin hydrolysis.

The most abundant hemicelluloses contain a β-1,4-xylan backbone chain, whereas the major hemicelluloses in softwoods are mannan-type with a backbone of β-1,4-linked mannose and glucose [28, 31]. Degradation of the hemicellulose linear β-1,4-linked main chain requires the action of endo-β-1,4-xylanase and β-xylosidase for β-1,4-xylan, or β-mannanase for β-1,4-mannan. Both types of hemicellulose main chain hydrolases were found in the TMC7 metagenome. Six endo-β-1,4-xylanase GH families, including GH5 subfamilies GH8, GH10, GH11, GH30_8, and GH51, were identified. Nine β-xylosidase GH families, including GH1, GH3, GH30_8, GH39, GH43 subfamilies, GH51, GH52, GH116, and GH120 were identified. Three β-mannosidase GH families, including GH1, GH2, and GH5, were identified.

A series of accessory or debranching enzymes is also required to completely degrade complex substituted xylans; these enzymes include arabinofuranosidases and various heteroglycan hydrolases [28]. The six arabinofuranosidase GH families GH2, GH43, GH51, GH127, GH137, and GH142 were identified. The TMC7 metagenome also showed highly diverse debranching enzymes for heteroglycan, including two α-mannosidase GH families (GH38 and GH125) for mannosyl and 11 GH families (GH1, GH2, GH4, GH16, GH27, GH31, GH35, GH36, GH42, GH53, and GH95). In addition, mannosyl and galactosyl are substituted by rhamnosyl (deoxy-mannosyl) and fucosyl (deoxy-galactosyl) in some species [29]. In the TMC7 metagenome, four α-fucosidase GH families (GH3, GH29, GH95, and GH141) were identified, and two GH families (GH78 and GH106) with α-rhamnosidase activity were identified.

In addition to these glycoside hydrolases, debranching of hemicellulose and pectin requires the action of glucuronidase, acetylesterases, acetyl xylan esterases, and feruloyl esterase. Seven GH families (GH4, GH28, GH67, CH88, GH105, GH115, and GH138) involved in the hydrolysis of uronic acid were found in the TMC7 metagenome. Nine CE families (CE1, CE3, CE4, CE6, CE7, CE8, CE9, CE10, and CE12) encoding acetylesterases or acetyl xylan esterases were identified. In addition, feruloyl esterase CE1 involved in the cleavage of hemicellulose and lignin was identified.

The TMC7 metagenome was particularly rich in genes encoding diverse lignocellulose-binding modules. Almost all action sites of the above hydrolases were covered, including 8 CBMs for cellulose (CBM3, CBM4, CBM6, CBM9, CBM16, CBM37, CBM44, and CBM46), 8 CBMs for xylan (CBM4, CBM6, CBM9, CBM31, CBM35, CBM37, CBM42, CBM44, and CBM54), 3 CBMs for mannan (CBM16, CBM23, and CBM35), 2 CBMs for galactose or lactose (CBM32 and CBM51), and 1 CBM for rhamnose (CBM67). According to the literature, thermophilic bacteria typically harbor multiple CBMs that are predicted to counteract the loss of binding affinity between thermophilic enzymes and their substrates at elevated temperatures [32].

### Distribution of TMC7 Microbes in Lignocellulose Degradation

**Fig. 4:**
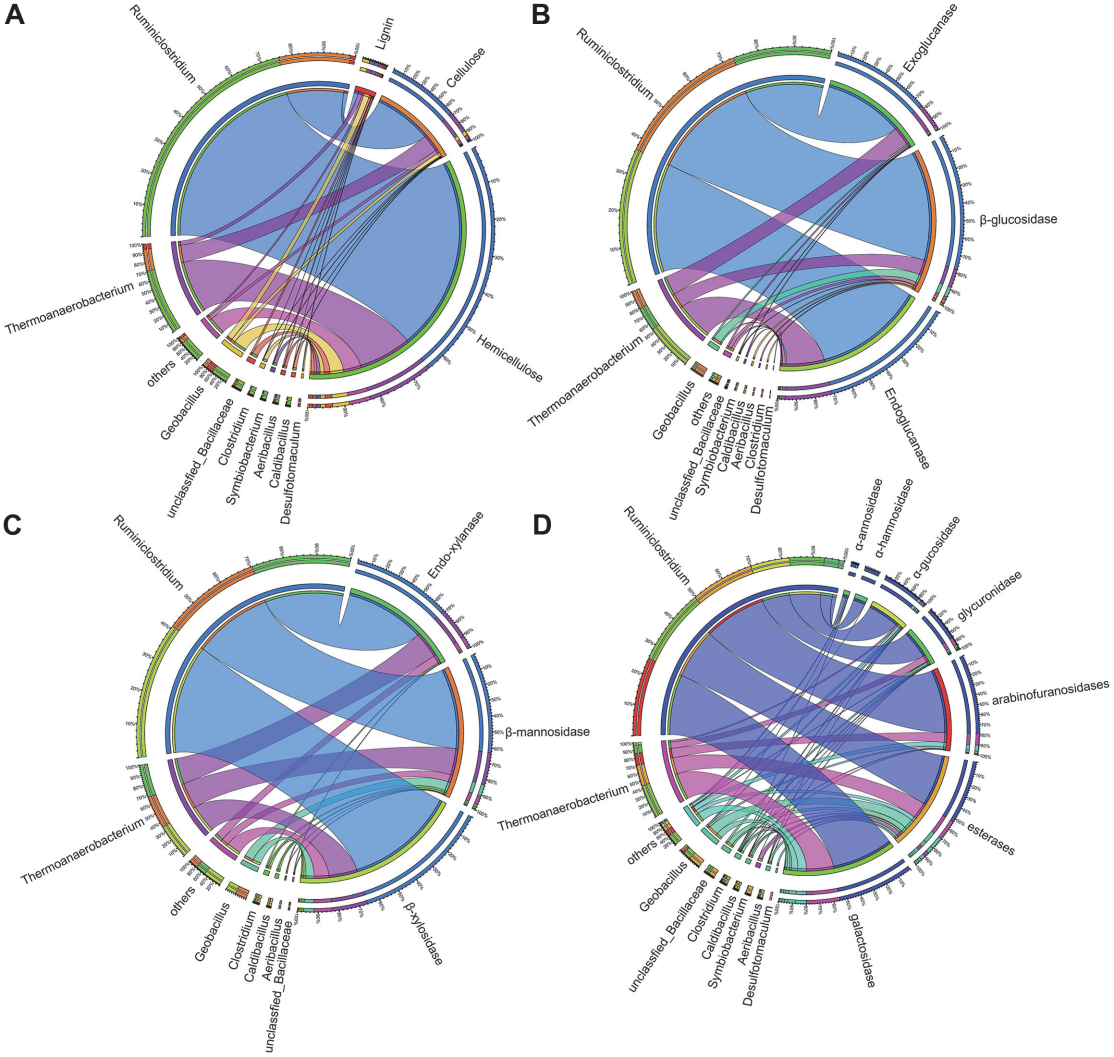
Distribution of microbes in lignocellulose degradation in TMC7. (**A**) Distribution of microbes in degradation of cellulose, hemicellulose, and lignin; (**B**) Distribution of microbes on cellulose degradation gene; (**C**) Distribution of microbes on hemicellulose main chain degradation-gene; (**D**) Distribution of microbes on hemicellulose debranching genes.

To determine the contribution of specific microbes and their potential interactions during lignocellulose degradation, the taxonomic affiliation of the annotated CAZymes was calculated and summarized according to their target structure on lignocellulose. The results were visualized using Circos and are shown in Fig. 4. The CAZymes that degrade the three main components (cellulose, hemicellulose (combined pectin), and lignin) are summarized in Fig. 4A. We observed different labor division strategies among them.

The degradation of cellulose was dominated by *Ruminiclostridium* and *Thermoanaerobacterium*, accounting for 68.25% and 20.45% of cellulose degradation genes, respectively. Regarding hemicellulose degradation, the ratio of contributed CAZymes was reduced to 65.01% *Ruminiclostridium* and 17.75% *Thermoanaerobacterium*, indicating that other low-abundance microbes contributed slightly more hemicellulose-related CAZymes than cellulose-related CAZymes. In contrast, the dominant genera *Ruminiclostridium* and *Thermoanaerobacterium* did not dominate lignin degradation in TMC7, accounting for only 19.98% and 20.14% of lignin degradation genes, respectively. This indicates that TMC7 invested few gene resources in lignin degradation.

In addition, the cellulose degradation genes are summarized as endoglucanase, exoglucanase, and β-glucosidase, as shown in Fig. 4B. Genes involves in degradation of the hemicellulose main chain are summarized as endo-β-1,4-xylanase, β-xylosidase, and β-mannosidase, as shown in Fig. 4C. The hemicellulose debranching genes arabinofuranosidases, α-mannosidase, α-rhamnosidase, galactosidase, α-fucosidase, glucuronidase, and esterases are visualized in Fig. 4D. Generally, *Ruminiclostridium* and *Thermoanaerobacterium* dominated the whole degradation process of lignocellulose; in contrast, complementarity among various microbes was also observed. Particularly, some low-abundance CAZymes were only identified in specific microbes (Fig. S1). Diverse CAZymes provided greater choice for complex lignocellulose structures, enhancing the adaptability of the consortium.

### Potential Contribution of TMC7 Microbes to Lignocellulose Degradation and Search for Sugar Cheaters

In the study of microbial consortium functions, microbial abundance is typically used as a basic index to calculate the contribution of each microbe. However, as labor is divided in a microbial consortium and opportunistic organisms (sugar cheaters) are prevalent, the functional diversity may be inconsistent with the relative taxonomic diversity. Specifically, some opportunistic organisms in the microbial consortium profit from the public goods, namely the nutrients released during lignocellulose degradation, but produce few lignocellulolytic enzymes [12]. Thus, exclusion of taxonomic abundance would calculate the absolute capability of specific microbes and find the cheaters. A CC value >1 indicates that the genera contribute more lignocellulolytic enzymes compared with their resource occupancy, and vice versa.

**Fig. 5:**
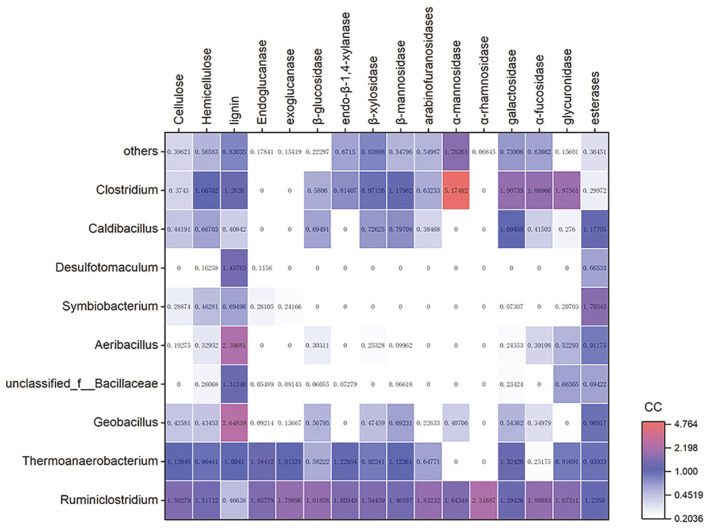
Heatmap showing contribution coefficients of microbes in a variety of lignocellulolytic activities in TMC7. We defined the contribution coefficient (CC) to calculate the contribution of a specific genus to a specific function as: CC = (specific genus abundance of specific function)/(specific genus abundance). CAZymes related to each function were selected according to the CAZymes list in Table 2 and profiles in Fig. 2.

The CC of the top nine genera with an abundance of more than 1% were calculated and visualized in a heatmap (Fig. 5, detailed calculation of CC in Table S4). The CC values of the most abundant *Ruminiclostridium* were >1 in all annotated lignocellulolytic functions except for the value of 0.466 CC in lignin, indicating its dominant role in lignocellulose degradation of TMC7. The second abundant *Thermoanaerobacterium* had >1 CC in endoglucanase, exoglucanase, endo-β-1,4-xylanase, β-mannosidase, and galactosidase, whereas it contributed little to α-mannosidase, α-rhamnosidase, and α-fucosidase. Notably, although it only showed an abundance of 1.26%, *Clostridium* contributed to most lignocellulolytic functions, particularly α-mannosidase, galactosidase, α-fucosidase, and glucuronidase. Moreover, Fig. 5 also shows that *Symbiobacterium* and *Desulfotomaculum* contributed little to most lignocellulolytic functions except for AA and esterases, indicating that they serve as potential “sugar cheaters” during lignocellulose degradation by TMC7.

In addition, as the alkali-pretreatment process removed most of lignin and pectin esters, and TMC7 exhibited little AA and esterase activity. Accordingly, we could see that the dominant genera *Ruminiclostridium* and *Thermoanaerobacterium* did not dominate lignin and pectin degradation in TMC7. This implied that TMC7 invested few resources in lignin and pectin degradation.

CCs may provide an important judgment criterion for studying functional microbial consortia. Because of the ubiquity of lignocellulolytic CAZymes in nature, enrichment of natural cultures may yield microbial consortia rich in lignocellulolytic CAZymes. However, there is no standard for calculating the obtained microbial consortia except for the basic degradation performance index, such as weight loss of the substrates. In microbial consortia with effective lignocellulolytic activity, the contributors (microbes providing lignocellulolytic enzymes) should gain preferential access to the substrate to avoid “tragedy of the commons” [12]. Otherwise, excessive consumption of resources by sugar cheaters may disable the consortium function. By applying the CC value, we could approximately distinguish the potential contributors and cheaters. Combining this information with the relative taxonomic abundance suggested that potential contributors were dominant, whereas potential cheaters were rather low. This indicates that the effective lignocellulolytic microbial consortium TMC7 had a good balance between effective degraders (contributors) and sugar cheaters.

## Conclusion

In summary, lignocellulose degradation by the thermophilic microbial consortium TMC7 was analyzed by metagenomic sequencing. The CAZymes accessing each component of natural lignocellulose were summarized to determine profiles for lignocellulose degradation of TMC7. The diversity CAZymes in TMC7 provides a treasury for the discovery of efficient thermophilic lignocellulolytic enzymes for biotechnological applications. Although this metagenomic study of TMC7 revealed the genetic and enzymatic lignocellulolytic potential of the microbial community, further omics studies using various substrates are required to identify the specific contributions of the microbes to the degradation of lignocellulose components.

Specially, we provided a new tool for studying the potential contribution of microbes in TMC7 to lignocellulose degradation. In accordance with the hypothesis of Jiménez *et al*., the balance between lignocellulolytic contributors and sugar cheaters was demonstrated by calculating the CC. *Ruminiclostridium* and *Thermoanaerobacterium* may be the dominant contributors, whereas *Symbiobacterium* and *Desulfotomaculum* may serve as “sugar cheaters” in lignocellulose degradation by TMC7. However, more detailed mathematical models evaluated using various comparative tests are needed to specify the contributors and cheaters in the microbial consortia.

## Supplemental Materials

Supplementary data for this paper are available on-line only at http://jmb.or.kr.

## Figures and Tables

**Table 1:** List of TMC7 CAZymes involved in lignocellulose degradation on class level.

Class	Class counts	Gene counts	Reads number
AA	4	57	125248
CBM	27	227	545456
CE	11	250	483142
GH	102	643	2556056
GT	26	445	1005060
PL	14	36	154000

Presented are the total numbers of CAZyme classes and the relative reads number.

**Table 2:** List of TMC7 CAZymes involved in lignocellulose degradation on family level.

	Description	Gene Counts	Reads Number
LMEs			
AA2	Manganese peroxidase; versatile peroxidase; lignin peroxidase; peroxidase	1	1240
AA4	Vanillyl-alcohol oxidase	15	56066
AA6	1,4-benzoquinone reductase	39	66334
AA7	Glucooligosaccharide oxidase; chitooligosaccharide oxidase	2	1608
SUM		57	125248
Lignocellulose-binding modules			
CBM3	Cellulose binding	7	28046
CBM4	Cellulose, xylan, β-1,3-glucan, β-1,3-1,4-glucan, β-1,6-glucan binding	3	21588
CBM6	Cellulose and β-1,4-xylan binding	6	37160
CBM9	Xylan and cellulose binding	9	6772
CBM16	Cellulose and glucomannan binding	9	52702
CBM23	Mannan binding	1	108
CBM31	β-1,3-xylan binding	2	1040
CBM32	Galactose and lactose binding	3	4634
CBM35	Xylan, mannan and β-galactan binding	6	24400
CBM37	Cellulose and xylan binding	10	33850
CBM42	Arabinoxylan binding	2	6504
CBM44	Cellulose and xyloglucan binding	1	3110
CBM46	Cellulose binding	2	204
CBM51	Galactose binding	4	6940
CBM54	Xylan and glucan binding	3	560
CBM61	β-1,4-galactan binding	5	38294
CBM67	l-rhamnose binding	4	146
SUM		77	266058
Cellulases and Hemicellulases			
GH1	β-glucosidase; β-galactosidase; β-mannosidase; β-glucuronidase; β-xylosidase	29	49444
GH3	β-glucosidase; β-xylosidase; α-l-fucosidase	29	100644
GH5's subfamilies (GH5_1 GH5_2 GH5_4 GH5_19 GH5_22 GH5_25 GH5_34 GH5_35 GH5_36 GH5_37)	Endo-β-1,4-glucanase; endo-β-1,4-xylanase; β-glucosidase; β-mannosidase; cellobiohydrolase	16	84872
GH8	Endoglucanase; endo-1,4-β-xylanase	1	4926
GH9	Endoglucanase; endo-β-1,3(4)-glucanase; β-glucosidase; cellobiohydrolase	16	100654
GH16	Endo-1,3(4)-β-glucanase; xyloglucanase; endo-β-1,4-galactosidase	3	4480
GH26	β-mannanase; β-1,3-xylanase; endo-β-1,3(4)-glucanase	7	59378
GH30_8	Endo-β-1,4-xylanase; β-glucosidase; β-glucuronidase; β-xylosidase	1	4114
GH44	Endoglucanase; xyloglucanase	1	11206
GH48	Cellobiohydrolase; endo-β-1,4-glucanase	3	20574
GH51	Endoglucanase; endo-β-1,4-xylanase; β-xylosidase; α-l-arabinofuranosidase	8	32728
GH74	Endoglucanase; cellobiohydrolase; xyloglucanase	7	40172
GH81	Endo-β-1,3-glucanase	1	6332
GH116	β-glucosidase; β-xylosidase	1	1236
SUM		123	520760
Hemicellulases			
GH2	β-galactosidase; β-mannosidase; α-l-arabinofuranosidase)	25	168714
GH4	α-glucosidase; α-galactosidase; α-glucuronidase	22	47864
GH10	Endo-β-1,3(4)-xylanase	20	141490
GH11	Endo-β-1,3(4)-xylanase	1	7380
GH27	α-galactosidase	1	8546
GH29	α-l-fucosidase	6	25280
GH31	α-glucosidase; α-galactosidase; α-xylosidase	14	47634
GH35	β-galactosidase	2	16812
GH36	α-galactosidase	11	61974
GH37	α-trehalase	1	11212
GH38	α-mannosidase	8	26766
GH39	β-xylosidase	6	38952
GH42	β-galactosidase; α-l-arabinopyranosidase	8	15742
GH43's subfamily (GH43_1 GH43_2 GH43_4 GH43_10 GH43_11 GH43_12 GH43_17 GH43_20 GH43_22 GH43_24 GH43_26 GH43_27 GH43_29 GH43_35)	β-xylosidase; α-l-arabinofuranosidase	23	139416
GH52	β-xylosidase	1	11314
GH53	Endo-β-1,4-galactanase	5	46350
GH76	α-1,6-mannanase	7	17582
GH78	α-l-rhamnosidase	3	156
GH95	α-l-fucosidase; α-l-galactosidase	11	44410
GH106	α-l-rhamnosidase	8	55788
GH113	β-mannanase	1	1470
GH120	β-xylosidase	5	17812
GH125	Exo-α-1,6-mannosidase	1	12
GH127	β-l-arabinofuranosidase	3	31670
GH137	β-l-arabinofuranosidase	2	38
GH141	α-l-fucosidase	1	9140
GH142	β-l-arabinofuranosidase	1	56
SUM		197	993580
Hemicellulases and pectin			
CE1	Acetyl xylan esterase; cinnamoyl esterase; feruloyl esterase; carboxylesterase;	44	60674
CE3	Acetyl xylan esterase	17	39112
CE4	Acetyl xylan esterase; chitin deacetylase;	95	152940
CE6	Acetyl xylan esterase	1	978
CE7	Acetyl xylan esterase	16	50096
CE8	Pectin methylesterase	8	16440
CE9	N-acetylglucosamine 6-phosphate deacetylase	20	47026
CE10	Arylesterase; carboxyl esterase	21	53606
CE12	Rhamnogalacturonan acetylesterase; acetyl xylan esterase	11	26316
GH28	Polygalacturonase; rhamnogalacturonase	5	28470
GH67	Xylan α-1,2-glucuronidase	4	29962
GH88	β-glucuronyl hydrolase	2	9944
GH105	Rhamnogalacturonyl hydrolase	10	49172
GH115	Xylan α-1,2-glucuronidase	1	12512
GH138	α-galacturonidase	2	2772
SUM		257	580020

Presented are the total numbers of CAZyme families and the relative reads number.
