# Supplementary material for: Metagenomic Insight into Lignocellulose Degradation of the Thermophilic Microbial Consortium TMC7
Source: J Microbiol Biotechnol. 2021 Jun 29;31(8):1123–33. doi: 10.4014/jmb.2106.06015 (PMC9706030; doi:10.4014/jmb.2106.06015)
Supplement: Supplementary file 1 [file jmb-31-8-1123-supple.pdf]

J. Microbiol. Biotechnol.
https://doi.org/10.4014/jmb.2106.06015

Figures S1 CAZymes distribution in microbes in TMC7

Endoglucanase
GH5's subfamilies, GH8, GH9, GH16, GH26, GH44, GH48, GH51, GH74, GH81

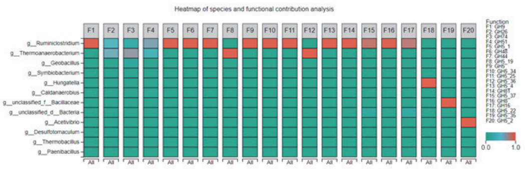

β-glucosidase
GH1, GH3, GH5's subfamilies, GH9, GH30_8, GH116

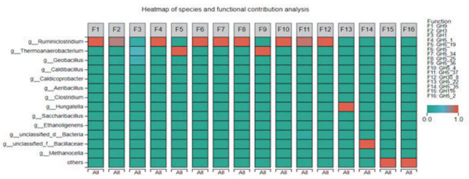

Exoglucanase
GH5's subfamilies, GH9, GH48, GH74

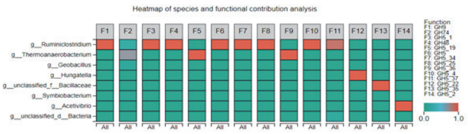

Endo-β-1,4-xylanase 6GHs
GH5, GH8, GH10, GH11, GH30_8, GH51

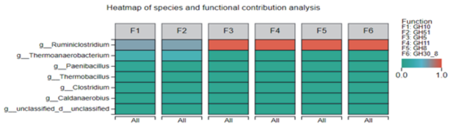

β-xylosidase 9GHs
GH1, GH3, GH30_8, GH39, GH43's subfamilies, GH51, GH52, GH116, GH120

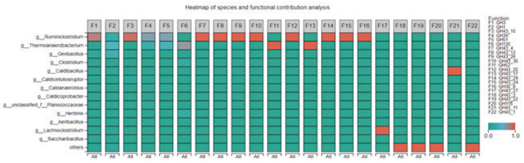

β-mannosidase
GH1, GH2, GH5

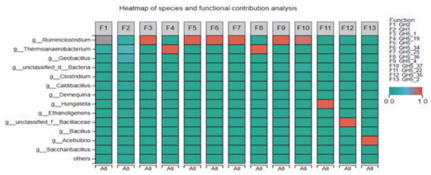

arabinofuranosidases 6GHs
GH2, GH43, GH51, GH127, GH137, GH142

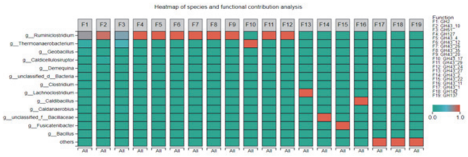

α-mannosidase 2GHs
GH38, GH125

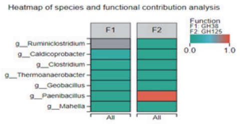

α-rhamnosidase 2GHs
GH78, GH106

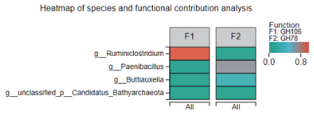

galactosidase 11GHs
GH1, GH2, GH4, GH16, GH27, GH31, GH35, GH36, GH42, GH53, GH95

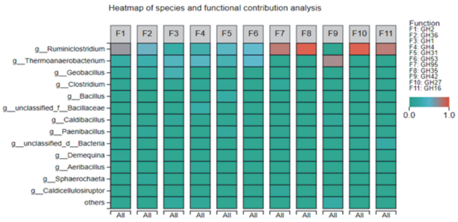

α-fucosidase 4GHs
GH3, GH29, GH95, GH141

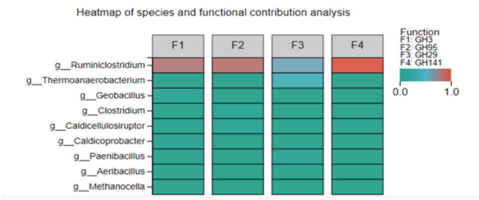

Glycuronidase 7GHs
GH4, GH28, GH67, CH88, GH105, GH115, GH138

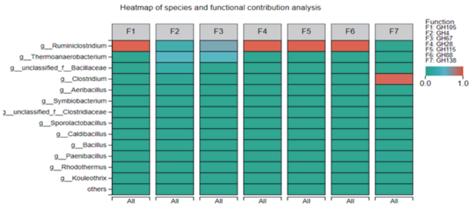

Esterases 9CEs
CE1, CE3, CE4, CE6, CE7, CE8, CE9, CE10, CE12

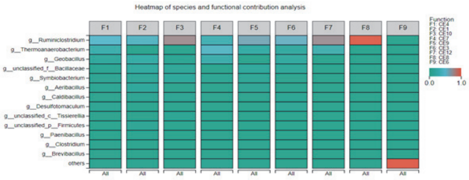

Table S1. Comparation of the metagenomic assembly of several lignocellulolytic microbial consortia

ID	resource	Clean reads	Contig number	N50(bp)	Total length(bp)	Dominate phyla	Author
TMC7	Compost<br>structurally stable consortium	83, 777, 510	8, 641	35, 005	45, 945, 177	>99% *Firmicutes*	This study
EMSD5	Compost<br>structurally stable consortium	864, 196	17, 908	16, 757	39, 141, 874	25.7% *Firmicutes* , 38.6% *Proteobacteria* 4.2% *Bacteroidetes*	Zhu et al., 2016
ZCTH02	Compost<br>structurally stable consortium	3, 046, 968	13, 240	17, 996	27, 962, 858	86% *Firmicutes*	Lemos et al., 2017
RSA	Compost<br>structurally stable consortium	59, 627, 950	83, 237	2, 153	129, 551, 542	79.64% *Actinobacteria* , 14.14% *Bacteroidetes* , 5.84% *Chloroflexi*	Wang et al., 2016
gut microbiom e	Common Black Slug Arion ater gut<br>natural microbial consortium	25, 996, 846	48, 089	1800	--	88.15% *Proteobacteria* , 10.53% *Bacteroidetes*	Joynson et al., 2017
ERAC	Cow rumen enrichment	21M	103,541	21,714	197M	*Firmicutes, Synergistetes, Spirochaetes, Proteobacteia, Bacteroidetes*	Tomazetto et al., 2020
RSV	Vermicompost enrichment	18, 831, 906	--	22,185	--	56.91% *Firmicutes* 28.32% *Proteobacteria* ， 12.22% *Bacteroidetes*	Gavande, et al., 2021
BD-C	Beaver droppings<br>3 years cultivation with cellulose	74,999,337	5,010	92,758	78M	*Firmicutes, Bacteroidetes, Chloroflexi,* and *Proteobacteria*	Wong et al., 2017
BD-PH	Beaver droppings<br>3 years cultivation with poplar hydrolysate	78,144,385	10,553	68,167	81.5M	*Firmicutes, Bacteroidetes, Chloroflexi,* and *Proteobacteria*	Wong et al., 2017
MR-C	Moose rumen<br>3 years cultivation with cellulose	78,144,385	10,553	71,246	58.3M	*Firmicutes, Bacteroidetes, Chloroflexi,* and *Proteobacteria*	Wong et al., 2017
MR-PH	Moose rumen<br>3 years cultivation with poplar hydrolysate	88,305,224	6,941	106,046	67.6M	*Firmicutes, Bacteroidetes, Chloroflexi,* and *Proteobacteria*	Wong et al., 2017

Interestingly, we found TMC7 in this study and the four microbial consortia in the study of Wong et al. had rather low contigs and high N50. The common of them were that they were obtained by long-term domestication using lignocellulose materials. The results of TMC7 in our study and these four consortia in Wong's study both indicated that the consortia would get a more specialized and stable microbial composition after long-term enrichment. This is important for study of the consortium, the "dilution-to-extinction" during the repeating subcultivation process (Lee et al. 2013) can exclude the "useless microbes" and obtain the information of "structure microbes" of the consortium.

Reference

Gavande, P.V., Basak, A., Sen, S. et al. Functional characterization of thermotolerant microbial consortium for lignocellulolytic enzymes with central role of Firmicutes in rice straw depolymerization. Sci Rep 11, 3032 (2021). https://doi.org/10.1038/s41598-021-82163-x

Joynson, R., Pritchard, L., Osemwekha, E., & Ferry, N. (2017). Metagenomic analysis of the gut microbiome of the common black slug Arion ater in search of novel lignocellulose degrading enzymes. Front. Microbiol. 8, 2181. doi: 10.3389/fmicb.2017.02181

Lee, D. J., Show, K. Y., & Wang, A.. (2013). Unconventional approaches to isolation and enrichment of functional microbial consortium - a review. Bioresour. Technol., 136(3), 697-706. https://doi.org/10.1016/j.biortech.2013.02.075

Lemos, L. N., Pereira, R. V., Quaggio, R. B., Martins, L. F., Moura, L. M. S., da Silva, A. R. et al. (2017). Genome-centric analysis of a thermophilic and Cellulolytic Bacterial Consortium Derived from composting. Front. Microbiol. 8, 644. doi:10.3389/fmicb.2017.00644.

Wang, C., Dong, D., Wang, H. S., Müller, K., Qin, Y., Wang, H. L., et al (2016). Metagenomic analysis of microbial consortia enriched from compost: new insights into the role of Actinobacteria in lignocellulose decomposition. Biotechnol. Biofuels 9, 22. doi:10.1186/s13068-016-0440-2.

Wong MT, Wang W, Couturier M, et al. Comparative Metagenomics of Cellulose- and Poplar Hydrolysate-Degrading Microcosms from Gut Microflora of the Canadian Beaver (Castor canadensis) and North American Moose (Alces americanus) after Long-Term Enrichment. (2017). Front. Microbiol. 8:2504. DOI: 10.3389/fmicb.2017.02504.

Zhu, N., Yang, J., Ji, L., Liu, J., Yang, Y., and Yuan, H. (2016). Metagenomic and metaproteomic analyses of a corn stover-adapted microbial consortium emsd5 reveal its taxonomic and enzymatic basis for degrading lignocellulose. Biotechnol. Biofuels 9, 243. doi:10.1186/s13068-016-0658-z.

Table S2. Taxonomic abundance of TMC7 metagenome.

Domain	Kingdom	Phylum	Class	Order	Family	Genus	Reads_num	
d__Bacteria	k__unclassified_d__Bacteria	p__Firmicutes	c__Clostridia	o__Clostridiales	f__Ruminococcaceae	g__Ruminiclostridium	35902460	**42.85%**
d__Bacteria	k__unclassified_d__Bacteria	p__Firmicutes	c__Clostridia	o__Thermoanaerobacterales	f__Thermoanaerobacterales_Family_III._Incertae_Sedis	g__Thermoanaerobacterium	15423128	**18.41%**
d__Bacteria	k__unclassified_d__Bacteria	p__Firmicutes	c__Bacilli	o__Bacillales	f__Bacillaceae	g__Geobacillus	8747348	**10.44%**
d__Bacteria	k__unclassified_d__Bacteria	p__Firmicutes	c__Bacilli	o__Bacillales	f__Bacillaceae	g__unclassified_f__Bacillaceae	6268772	**7.48%**
d__Bacteria	k__unclassified_d__Bacteria	p__Firmicutes	c__Bacilli	o__Bacillales	f__Bacillaceae	g__Aeribacillus	2220868	**2.65%**
d__Bacteria	k__unclassified_d__Bacteria	p__Firmicutes	c__Clostridia	o__Clostridiales	f__Symbiobacteriaceae	g__Symbiobacterium	2067626	**2.47%**
d__Bacteria	k__unclassified_d__Bacteria	p__Firmicutes	c__Clostridia	o__Clostridiales	f__Peptococcaceae	g__Desulfotomaculum	1949868	**2.33%**
d__Bacteria	k__unclassified_d__Bacteria	p__Firmicutes	c__Bacilli	o__Bacillales	f__Bacillaceae	g__Caldibacillus	1308640	**1.56%**
d__Bacteria	k__unclassified_d__Bacteria	p__Firmicutes	c__Clostridia	o__Clostridiales	f__Clostridiaceae	g__Clostridium	1053662	**1.26%**
d__Bacteria	k__unclassified_d__Bacteria	p__Firmicutes	c__Bacilli	o__Bacillales	f__Bacillaceae	g__Bacillus	631324	**0.75%**
d__Bacteria	k__unclassified_d__Bacteria	p__Firmicutes	c__Bacilli	o__Bacillales	f__Bacillaceae	g__Anoxybacillus	596834	**0.71%**
d__Bacteria	k__unclassified_d__Bacteria	p__Firmicutes	c__Clostridia	o__Clostridiales	f__unclassified_o__Clostridiales	g__unclassified_o__Clostridiales	521966	**0.62%**
d__Bacteria	k__unclassified_d__Bacteria	p__Firmicutes	c__Tissierellia	o__unclassified_c__Tissierellia	f__unclassified_c__Tissierellia	g__unclassified_c__Tissierellia	505058	**0.60%**
d__Bacteria	k__unclassified_d__Bacteria	p__Firmicutes	c__Clostridia	o__Clostridiales	f__Lachnospiraceae	g__Herbinix	504260	**0.60%**
d__Bacteria	k__unclassified_d__Bacteria	p__Firmicutes	c__Clostridia	o__Thermoanaerobacterales	f__Thermoanaerobacteraceae	g__Thermoanaerobacter	452902	**0.54%**
d__Bacteria	k__unclassified_d__Bacteria	p__Firmicutes	c__Clostridia	o__Clostridiales	f__Clostridiaceae	g__Fervidicella	408822	**0.49%**
d__Bacteria	k__unclassified_d__Bacteria	p__Firmicutes	c__Bacilli	o__Bacillales	f__Paenibacillaceae	g__Paenibacillus	356652	**0.43%**
d__Bacteria	k__unclassified_d__Bacteria	p__Firmicutes	c__Clostridia	o__Clostridiales	f__Clostridiaceae	g__Caloramator	239948	**0.29%**
d__Bacteria	k__unclassified_d__Bacteria	p__Firmicutes	c__Clostridia	o__Thermoanaerobacterales	f__Thermoanaerobacteraceae	g__Tepidanaerobacter	237790	**0.28%**
d__Bacteria	k__unclassified_d__Bacteria	p__unclassified_d__Bacteria	c__unclassified_d__Bacteria	o__unclassified_d__Bacteria	f__unclassified_d__Bacteria	g__unclassified_d__Bacteria	231408	**0.28%**
d__Bacteria	k__unclassified_d__Bacteria	p__Firmicutes	c__Clostridia	o__Clostridiales	f__Ruminococcaceae	g__Ruminococcus	215406	**0.26%**
d__Bacteria	k__unclassified_d__Bacteria	p__Firmicutes	c__Clostridia	o__unclassified_c__Clostridia	f__unclassified_c__Clostridia	g__unclassified_c__Clostridia	161504	**0.19%**
d__Bacteria	k__unclassified_d__Bacteria	p__Firmicutes	c__Clostridia	o__Clostridiales	f__Clostridiaceae	g__Thermotalea	141796	**0.17%**
d__Bacteria	k__unclassified_d__Bacteria	p__Firmicutes	c__Clostridia	o__Thermoanaerobacterales	f__Thermoanaerobacterales_Family_III._Incertae_Sedis	g__Thermosediminibacter	136264	**0.16%**
d__Bacteria	k__unclassified_d__Bacteria	p__Firmicutes	c__Clostridia	o__Clostridiales	f__Caldicoprobacteraceae	g__Caldicoprobacter	109300	**0.13%**
d__Bacteria	k__unclassified_d__Bacteria	p__Firmicutes	c__Clostridia	o__Clostridiales	f__Lachnospiraceae	g__Lachnoclostridium	103952	**0.12%**
d__Bacteria	k__unclassified_d__Bacteria	p__Firmicutes	c__Clostridia	o__Clostridiales	f__Clostridiaceae	g__Thermobrachium	101312	**0.12%**
d__Bacteria	k__unclassified_d__Bacteria	p__Firmicutes	c__Clostridia	o__Clostridiales	f__Peptococcaceae	g__Desulfitibacter	95862	**0.11%**
d__Bacteria	k__unclassified_d__Bacteria	p__Firmicutes	c__Clostridia	o__Thermoanaerobacterales	f__Thermoanaerobacterales_Family_III._Incertae_Sedis	g__Caldicellulosiruptor	91362	**0.11%**
d__Bacteria	k__unclassified_d__Bacteria	p__Firmicutes	c__Clostridia	o__Clostridiales	f__Ruminococcaceae	g__Acetivibrio	85012	**0.10%**
d__Bacteria	k__unclassified_d__Bacteria	p__Synergistetes	c__Synergistia	o__Synergistales	f__Synergistaceae	g__Anaerobaculum	84046	**0.10%**
d__Bacteria	k__unclassified_d__Bacteria	p__Firmicutes	c__unclassified_p__Firmicutes	o__unclassified_p__Firmicutes	f__unclassified_p__Firmicutes	g__unclassified_p__Firmicutes	83854	**0.10%**
d__Bacteria	k__unclassified_d__Bacteria	p__Firmicutes	c__Clostridia	o__Clostridiales	f__Ruminococcaceae	g__Pseudobacteroides	78322	**0.09%**
d__Bacteria	k__unclassified_d__Bacteria	p__Firmicutes	c__Clostridia	o__Clostridiales	f__Clostridiaceae	g__unclassified_f__Clostridiaceae	72404	**0.09%**
d__Bacteria	k__unclassified_d__Bacteria	p__Firmicutes	c__Bacilli	o__Bacillales	f__Paenibacillaceae	g__Brevibacillus	68994	**0.08%**
d__Bacteria	k__unclassified_d__Bacteria	p__Firmicutes	c__Clostridia	o__Clostridiales	f__Clostridiaceae	g__Caldisalinibacter	64048	**0.08%**
d__Bacteria	k__unclassified_d__Bacteria	p__Firmicutes	c__Clostridia	o__Clostridiales	f__Lachnospiraceae	g__Blautia	60392	**0.07%**
d__Bacteria	k__unclassified_d__Bacteria	p__Firmicutes	c__Clostridia	o__Clostridiales	f__Peptococcaceae	g__Desulfosporosinus	59556	**0.07%**
d__Bacteria	k__unclassified_d__Bacteria	p__Firmicutes	c__Clostridia	o__Clostridiales	f__Clostridiaceae	g__Caloranaerobacter	57876	**0.07%**
d__Bacteria	k__unclassified_d__Bacteria	p__Firmicutes	c__Clostridia	o__Thermoanaerobacterales	f__Thermoanaerobacterales_Family_IV._Incertae_Sedis	g__Mahella	57560	**0.07%**
d__Bacteria	k__unclassified_d__Bacteria	p__Firmicutes	c__Clostridia	o__Clostridiales	f__Clostridiaceae	g__Alkaliphilus	57528	**0.07%**
d__Archaea	k__unclassified_d__Archaea	p__Euryarchaeota	c__Methanomicrobia	o__Methanocellales	f__Methanocellaceae	g__Methanocella	54550	**0.07%**
d__Bacteria	k__unclassified_d__Bacteria	p__Firmicutes	c__Clostridia	o__Clostridiales	f__Peptococcaceae	g__unclassified_f__Peptococcaceae	53934	**0.06%**
d__Bacteria	k__unclassified_d__Bacteria	p__Firmicutes	c__Clostridia	o__Clostridiales	f__Lachnospiraceae	g__unclassified_f__Lachnospiraceae	51788	**0.06%**
d__Bacteria	k__unclassified_d__Bacteria	p__Firmicutes	c__Clostridia	o__Clostridiales	f__Eubacteriaceae	g__Eubacterium	50702	**0.06%**
d__Bacteria	k__unclassified_d__Bacteria	p__Firmicutes	c__Clostridia	o__Clostridiales	f__Clostridiaceae	g__Oxobacter	48010	**0.06%**
d__Bacteria	k__unclassified_d__Bacteria	p__Firmicutes	c__Clostridia	o__Clostridiales	f__Peptostreptococcaceae	g__Peptoclostridium	45812	**0.05%**
d__Bacteria	k__unclassified_d__Bacteria	p__Firmicutes	c__Clostridia	o__Clostridiales	f__Defluviitaleaceae	g__unclassified_f__Defluviitaleaceae	44670	**0.05%**
d__Bacteria	k__unclassified_d__Bacteria	p__Firmicutes	c__Clostridia	o__Clostridiales	f__Syntrophomonadaceae	g__Syntrophomonas	40160	**0.05%**
d__Bacteria	k__unclassified_d__Bacteria	p__Firmicutes	c__Clostridia	o__Clostridiales	f__Ruminococcaceae	g__unclassified_f__Ruminococcaceae	35344	**0.04%**
d__Bacteria	k__unclassified_d__Bacteria	p__Firmicutes	c__Clostridia	o__Thermoanaerobacterales	f__Thermodesulfobiaceae	g__Thermodesulfobium	35276	**0.04%**
d__Bacteria	k__unclassified_d__Bacteria	p__Firmicutes	c__Clostridia	o__Thermoanaerobacterales	f__Thermoanaerobacteraceae	g__Caldanaerobius	34502	**0.04%**
d__Bacteria	k__unclassified_d__Bacteria	p__Firmicutes	c__Clostridia	o__Clostridiales	f__Peptococcaceae	g__Desulfurispora	34214	**0.04%**
d__Bacteria	k__unclassified_d__Bacteria	p__Firmicutes	c__Clostridia	o__Thermoanaerobacterales	f__Thermoanaerobacteraceae	g__Caldanaerobacter	34026	**0.04%**
d__Bacteria	k__unclassified_d__Bacteria	p__Firmicutes	c__Clostridia	o__Clostridiales	f__Clostridiaceae	g__Anaerosalibacter	32068	**0.04%**
d__Bacteria	k__unclassified_d__Bacteria	p__Firmicutes	c__Tissierellia	o__unclassified_c__Tissierellia	f__unclassified_c__Tissierellia	g__Sedimentibacter	30448	**0.04%**
d__Bacteria	k__unclassified_d__Bacteria	p__Firmicutes	c__Clostridia	o__Thermoanaerobacterales	f__Thermoanaerobacteraceae	g__Carboxydothermus	30446	**0.04%**
d__Bacteria	k__unclassified_d__Bacteria	p__Firmicutes	c__Clostridia	o__Halanaerobiales	f__Halanaerobiaceae	g__Halanaerobium	28580	**0.03%**
d__Bacteria	k__unclassified_d__Bacteria	p__Firmicutes	c__Clostridia	o__Clostridiales	f__Peptococcaceae	g__Thermincola	27400	**0.03%**
d__Bacteria	k__unclassified_d__Bacteria	p__Firmicutes	c__Clostridia	o__Thermoanaerobacterales	f__Thermoanaerobacteraceae	g__Thermacetogenium	26192	**0.03%**
d__Bacteria	k__unclassified_d__Bacteria	p__Firmicutes	c__Clostridia	o__Clostridiales	f__unclassified_o__Clostridiales	g__Gottschalkia	26046	**0.03%**
d__Bacteria	k__unclassified_d__Bacteria	p__Thermotogae	c__Thermotogae	o__Petrotogales	f__unclassified_o__Petrotogales	g__Defluviitoga	25926	**0.03%**
d__Bacteria	k__unclassified_d__Bacteria	p__Firmicutes	c__Bacilli	o__Bacillales	f__Bacillaceae	g__Lysinibacillus	24080	**0.03%**
d__Bacteria	k__unclassified_d__Bacteria	p__Firmicutes	c__Clostridia	o__Clostridiales	f__Clostridiaceae	g__Youngiibacter	23860	**0.03%**
d__Bacteria	k__unclassified_d__Bacteria	p__Firmicutes	c__Clostridia	o__Thermoanaerobacterales	f__Thermoanaerobacterales_Family_III._Incertae_Sedis	g__Thermovenabulum	23618	**0.03%**
d__Bacteria	k__unclassified_d__Bacteria	p__Firmicutes	c__Clostridia	o__Clostridiales	f__Eubacteriaceae	g__Acetobacterium	23370	**0.03%**
d__Bacteria	k__unclassified_d__Bacteria	p__Firmicutes	c__Clostridia	o__Clostridiales	f__Heliobacteriaceae	g__Heliobacterium	22618	**0.03%**
d__Bacteria	k__unclassified_d__Bacteria	p__Firmicutes	c__Clostridia	o__Clostridiales	f__Ruminococcaceae	g__Subdoligranulum	20788	**0.02%**
d__Bacteria	k__unclassified_d__Bacteria	p__Firmicutes	c__Clostridia	o__Clostridiales	f__Peptococcaceae	g__Desulfitobacterium	20714	**0.02%**
d__Bacteria	k__unclassified_d__Bacteria	p__Firmicutes	c__Bacilli	o__Bacillales	f__Paenibacillaceae	g__Aneurinibacillus	20124	**0.02%**
d__Bacteria	k__unclassified_d__Bacteria	p__Firmicutes	c__Bacilli	o__Bacillales	f__Bacillales_Family_X._Incertae_Sedis	g__Thermicanus	20036	**0.02%**
d__Bacteria	k__unclassified_d__Bacteria	p__Firmicutes	c__Negativicutes	o__Selenomonadales	f__Veillonellaceae	g__Thermosinus	20004	**0.02%**
d__Bacteria	k__unclassified_d__Bacteria	p__Bacteroidetes	c__Bacteroidia	o__Bacteroidales	f__unclassified_o__Bacteroidales	g__unclassified_o__Bacteroidales	19678	**0.02%**
d__Bacteria	k__unclassified_d__Bacteria	p__Firmicutes	c__Clostridia	o__Clostridiales	f__Clostridiaceae	g__Hungatella	18964	**0.02%**
d__Bacteria	k__unclassified_d__Bacteria	p__Firmicutes	c__Clostridia	o__Thermoanaerobacterales	f__Thermoanaerobacteraceae	g__Fervidicola	18834	**0.02%**
d__Bacteria	k__unclassified_d__Bacteria	p__Firmicutes	c__Clostridia	o__Clostridiales	f__Clostridiales_Family_XIII._Incertae_Sedis	g__Anaerovorax	18712	**0.02%**
d__Viruses	k__unclassified_d__Viruses	p__unclassified_d__Viruses	c__unclassified_d__Viruses	o__Caudovirales	f__unclassified_o__Caudovirales	g__unclassified_o__Caudovirales	18570	**0.02%**
d__Bacteria	k__unclassified_d__Bacteria	p__Firmicutes	c__Clostridia	o__Clostridiales	f__Ruminococcaceae	g__Anaerotruncus	18246	**0.02%**
d__Bacteria	k__unclassified_d__Bacteria	p__Firmicutes	c__Bacilli	o__Bacillales	f__Paenibacillaceae	g__Thermobacillus	17792	**0.02%**
d__Bacteria	k__unclassified_d__Bacteria	p__Firmicutes	c__Bacilli	o__Bacillales	f__Paenibacillaceae	g__Cohnella	17396	**0.02%**
d__Bacteria	k__unclassified_d__Bacteria	p__Firmicutes	c__Bacilli	o__Bacillales	f__Alicyclobacillaceae	g__Alicyclobacillus	17362	**0.02%**
d__Bacteria	k__unclassified_d__Bacteria	p__Firmicutes	c__Negativicutes	o__Selenomonadales	f__Veillonellaceae	g__Pelosinus	16258	**0.02%**
d__Bacteria	k__unclassified_d__Bacteria	p__Proteobacteria	c__Deltaproteobacteria	o__Bdellovibrionales	f__Bdellovibrionaceae	g__Bdellovibrio	15882	**0.02%**
d__Bacteria	k__unclassified_d__Bacteria	p__Firmicutes	c__Negativicutes	o__Selenomonadales	f__Veillonellaceae	g__Anaerovibrio	14848	**0.02%**
d__Bacteria	k__unclassified_d__Bacteria	p__Spirochaetes	c__Spirochaetia	o__Spirochaetales	f__Spirochaetaceae	g__Sphaerochaeta	14840	**0.02%**
d__Bacteria	k__unclassified_d__Bacteria	p__Firmicutes	c__Bacilli	o__Bacillales	f__Bacillaceae	g__Fictibacillus	14802	**0.02%**
d__Bacteria	k__unclassified_d__Bacteria	p__Firmicutes	c__Clostridia	o__Clostridiales	f__Lachnospiraceae	g__Robinsoniella	14688	**0.02%**
d__Bacteria	k__unclassified_d__Bacteria	p__Firmicutes	c__Bacilli	o__Bacillales	f__Bacillaceae	g__Tepidibacillus	14616	**0.02%**
d__Bacteria	k__unclassified_d__Bacteria	p__Firmicutes	c__Clostridia	o__Thermoanaerobacterales	f__Thermoanaerobacteraceae	g__Moorella	14562	**0.02%**
d__Bacteria	k__unclassified_d__Bacteria	p__Firmicutes	c__Clostridia	o__Clostridiales	f__Lachnospiraceae	g__Fusicatenibacter	14560	**0.02%**
d__Bacteria	k__unclassified_d__Bacteria	p__Firmicutes	c__Bacilli	o__Bacillales	f__Bacillaceae	g__Hydrogenibacillus	13850	**0.02%**
d__Bacteria	k__unclassified_d__Bacteria	p__Firmicutes	c__Clostridia	o__Clostridiales	f__Lachnospiraceae	g__Cellulosilyticum	13708	**0.02%**
d__Bacteria	k__unclassified_d__Bacteria	p__Firmicutes	c__Clostridia	o__Clostridiales	f__Lachnospiraceae	g__Dorea	13632	**0.02%**
d__Bacteria	k__unclassified_d__Bacteria	p__Firmicutes	c__Bacilli	o__Bacillales	f__unclassified_o__Bacillales	g__unclassified_o__Bacillales	13540	**0.02%**
d__Bacteria	k__unclassified_d__Bacteria	p__Spirochaetes	c__Spirochaetia	o__Spirochaetales	f__Spirochaetaceae	g__Treponema	13174	**0.02%**
d__Bacteria	k__unclassified_d__Bacteria	p__Proteobacteria	c__Deltaproteobacteria	o__Desulfobacterales	f__Desulfobulbaceae	g__Desulfotalea	12364	**0.01%**
d__Bacteria	k__unclassified_d__Bacteria	p__Firmicutes	c__Tissierellia	o__Tissierellales	f__Peptoniphilaceae	g__Peptoniphilus	11756	**0.01%**
d__Bacteria	k__unclassified_d__Bacteria	p__Firmicutes	c__Bacilli	o__Bacillales	f__Bacillaceae	g__Ornithinibacillus	11674	**0.01%**
d__Bacteria	k__unclassified_d__Bacteria	p__Firmicutes	c__Bacilli	o__Bacillales	f__Planococcaceae	g__Sporosarcina	11638	**0.01%**
d__Bacteria	k__unclassified_d__Bacteria	p__Firmicutes	c__Clostridia	o__Clostridiales	f__Lachnospiraceae	g__Tyzzerella	11448	**0.01%**
d__Viruses	k__unclassified_d__Viruses	p__unclassified_d__Viruses	c__unclassified_d__Viruses	o__Caudovirales	f__Siphoviridae	g__unclassified_f__Siphoviridae	11342	**0.01%**
d__Bacteria	k__unclassified_d__Bacteria	p__Firmicutes	c__Clostridia	o__Clostridiales	f__Peptococcaceae	g__Pelotomaculum	11204	**0.01%**
d__Bacteria	k__unclassified_d__Bacteria	p__Firmicutes	c__Clostridia	o__Clostridiales	f__Lachnospiraceae	g__Butyrivibrio	10728	**0.01%**
d__Bacteria	k__unclassified_d__Bacteria	p__Firmicutes	c__Bacilli	o__Lactobacillales	f__Enterococcaceae	g__Enterococcus	9932	**0.01%**
d__Bacteria	k__unclassified_d__Bacteria	p__Firmicutes	c__Clostridia	o__Clostridiales	f__Clostridiaceae	g__Clostridiisalibacter	9904	**0.01%**
d__Bacteria	k__unclassified_d__Bacteria	p__Firmicutes	c__Bacilli	o__Lactobacillales	f__Carnobacteriaceae	g__Carnobacterium	9344	**0.01%**
d__Bacteria	k__unclassified_d__Bacteria	p__Firmicutes	c__Bacilli	o__Bacillales	f__Bacillaceae	g__Caldalkalibacillus	8942	**0.01%**
d__Bacteria	k__unclassified_d__Bacteria	p__Firmicutes	c__Clostridia	o__Thermoanaerobacterales	f__Thermoanaerobacteraceae	g__Desulfovirgula	8912	**0.01%**
d__Bacteria	k__unclassified_d__Bacteria	p__Firmicutes	c__Clostridia	o__Clostridiales	f__Syntrophomonadaceae	g__Syntrophothermus	8794	**0.01%**
d__Bacteria	k__unclassified_d__Bacteria	p__Firmicutes	c__Clostridia	o__Clostridiales	f__Lachnospiraceae	g__Roseburia	8228	**0.01%**
d__Bacteria	k__unclassified_d__Bacteria	p__Firmicutes	c__Bacilli	o__Bacillales	f__Paenibacillaceae	g__Gorillibacterium	8164	**0.01%**
d__Bacteria	k__unclassified_d__Bacteria	p__Firmicutes	c__Clostridia	o__Clostridiales	f__Peptococcaceae	g__Dehalobacter	8094	**0.01%**
d__Bacteria	k__unclassified_d__Bacteria	p__Firmicutes	c__Clostridia	o__Clostridiales	f__Lachnospiraceae	g__Coprococcus	8056	**0.01%**
d__Bacteria	k__unclassified_d__Bacteria	p__Actinobacteria	c__Coriobacteriia	o__Eggerthellales	f__Eggerthellaceae	g__Adlercreutzia	7930	**0.01%**
d__Bacteria	k__unclassified_d__Bacteria	p__Firmicutes	c__Bacilli	o__Bacillales	f__Sporolactobacillaceae	g__Tuberibacillus	7832	**0.01%**
d__Archaea	k__unclassified_d__Archaea	p__Euryarchaeota	c__Methanomicrobia	o__unclassified_c__Methanomicrobia	f__unclassified_c__Methanomicrobia	g__unclassified_c__Methanomicrobia	7788	**0.01%**
d__Bacteria	k__unclassified_d__Bacteria	p__Bacteroidetes	c__Cytophagia	o__Cytophagales	f__Cytophagaceae	g__Spirosoma	7646	**0.01%**
d__Bacteria	k__unclassified_d__Bacteria	p__Firmicutes	c__Clostridia	o__Clostridiales	f__unclassified_o__Clostridiales	g__Intestinimonas	7638	**0.01%**
d__Bacteria	k__unclassified_d__Bacteria	p__Firmicutes	c__Clostridia	o__Clostridiales	f__Lachnospiraceae	g__Marvinbryantia	7614	**0.01%**
d__Bacteria	k__unclassified_d__Bacteria	p__Firmicutes	c__Clostridia	o__Clostridiales	f__unclassified_o__Clostridiales	g__Pseudoflavonifractor	7528	**0.01%**
d__Archaea	k__unclassified_d__Archaea	p__Euryarchaeota	c__unclassified_p__Euryarchaeota	o__unclassified_p__Euryarchaeota	f__unclassified_p__Euryarchaeota	g__unclassified_p__Euryarchaeota	7242	**0.01%**
d__Bacteria	k__unclassified_d__Bacteria	p__Cyanobacteria	c__unclassified_p__Cyanobacteria	o__Chroococcales	f__unclassified_o__Chroococcales	g__Acaryochloris	7090	**0.01%**
d__Bacteria	k__unclassified_d__Bacteria	p__Firmicutes	c__Clostridia	o__Clostridiales	f__Clostridiaceae	g__Butyricicoccus	7056	**0.01%**
d__Bacteria	k__unclassified_d__Bacteria	p__Bacteroidetes	c__Bacteroidia	o__Bacteroidales	f__Porphyromonadaceae	g__Fermentimonas	7052	**0.01%**
d__Bacteria	k__unclassified_d__Bacteria	p__Firmicutes	c__Clostridia	o__Clostridiales	f__Peptostreptococcaceae	g__Peptostreptococcus	6954	**0.01%**
d__Archaea	k__unclassified_d__Archaea	p__Euryarchaeota	c__Methanomicrobia	o__Methanosarcinales	f__Methanosarcinaceae	g__Methanosarcina	6930	**0.01%**
d__Bacteria	k__unclassified_d__Bacteria	p__Firmicutes	c__Bacilli	o__Bacillales	f__Planococcaceae	g__Viridibacillus	6794	**0.01%**
d__Bacteria	k__unclassified_d__Bacteria	p__Firmicutes	c__Clostridia	o__Clostridiales	f__Oscillospiraceae	g__Oscillibacter	6784	**0.01%**
d__Bacteria	k__unclassified_d__Bacteria	p__Firmicutes	c__Clostridia	o__Clostridiales	f__Gracilibacteraceae	g__Gracilibacter	6714	**0.01%**
d__Bacteria	k__unclassified_d__Bacteria	p__Firmicutes	c__Clostridia	o__Clostridiales	f__Clostridiales_Family_XVII._Incertae_Sedis	g__Thermaerobacter	6636	**0.01%**
d__Bacteria	k__unclassified_d__Bacteria	p__Tenericutes	c__Mollicutes	o__unclassified_c__Mollicutes	f__unclassified_c__Mollicutes	g__unclassified_c__Mollicutes	6514	**0.01%**
d__Bacteria	k__unclassified_d__Bacteria	p__Firmicutes	c__Clostridia	o__Clostridiales	f__Ruminococcaceae	g__Ethanoligenens	6482	**0.01%**
d__Bacteria	k__unclassified_d__Bacteria	p__Firmicutes	c__Bacilli	o__Bacillales	f__Bacillaceae	g__Thalassobacillus	6082	**0.01%**
d__Bacteria	k__unclassified_d__Bacteria	p__Firmicutes	c__Bacilli	o__Bacillales	f__Sporolactobacillaceae	g__Sporolactobacillus	6060	**0.01%**
d__Eukaryota	k__Fungi	p__unclassified_k__Fungi	c__unclassified_k__Fungi	o__Mucorales	f__Mucoraceae	g__Mucor	5902	**0.01%**
d__Bacteria	k__unclassified_d__Bacteria	p__Firmicutes	c__Bacilli	o__Bacillales	f__Planococcaceae	g__Ureibacillus	5878	**0.01%**
d__Bacteria	k__unclassified_d__Bacteria	p__Spirochaetes	c__Spirochaetia	o__Spirochaetales	f__Spirochaetaceae	g__Spirochaeta	5772	**0.01%**
d__Bacteria	k__unclassified_d__Bacteria	p__Firmicutes	c__Bacilli	o__Bacillales	f__Bacillaceae	g__Virgibacillus	5608	**0.01%**
d__Bacteria	k__unclassified_d__Bacteria	p__Aquificae	c__Aquificae	o__Aquificales	f__Aquificaceae	g__Thermocrinis	5492	**0.01%**
d__Bacteria	k__unclassified_d__Bacteria	p__Thermotogae	c__Thermotogae	o__Thermotogales	f__Fervidobacteriaceae	g__Fervidobacterium	5442	**0.01%**
d__Archaea	k__unclassified_d__Archaea	p__Euryarchaeota	c__Methanobacteria	o__Methanobacteriales	f__Methanobacteriaceae	g__Methanobrevibacter	5288	**0.01%**
d__Bacteria	k__unclassified_d__Bacteria	p__Firmicutes	c__Clostridia	o__Thermoanaerobacterales	f__Thermoanaerobacterales_Family_III._Incertae_Sedis	g__Syntrophaceticus	5288	**0.01%**
d__Bacteria	k__unclassified_d__Bacteria	p__Firmicutes	c__Bacilli	o__Bacillales	f__Bacillaceae	g__Calditerricola	5246	**0.01%**
d__Bacteria	k__unclassified_d__Bacteria	p__Proteobacteria	c__Betaproteobacteria	o__Burkholderiales	f__Burkholderiaceae	g__Burkholderia	5052	**0.01%**
d__Viruses	k__unclassified_d__Viruses	p__unclassified_d__Viruses	c__unclassified_d__Viruses	o__Caudovirales	f__Myoviridae	g__unclassified_f__Myoviridae	4946	**0.01%**
d__Bacteria	k__unclassified_d__Bacteria	p__Firmicutes	c__Bacilli	o__Bacillales	f__Alicyclobacillaceae	g__Effusibacillus	4942	**0.01%**

d__Bacteria	k__unclassified_d__Bacteria	p__Dictyoglomi	c__Dictyoglomia	o__Dictyoglomales	f__Dictyoglomaceae	g__Dictyoglomus	4746	**0.01%**
d__Bacteria	k__unclassified_d__Bacteria	p__Firmicutes	c__Bacilli	o__Bacillales	f__Alicyclobacillaceae	g__Tumebacillus	4608	**0.01%**
d__Bacteria	k__unclassified_d__Bacteria	p__Firmicutes	c__Clostridia	o__Thermoanaerobacterales	f__unclassified_o__Thermoanaerobacterales	g__unclassified_o__Thermoanaerobacterales	4604	**0.01%**
d__Bacteria	k__unclassified_d__Bacteria	p__Actinobacteria	c__Actinobacteria	o__Micrococcales	f__Demequinaceae	g__Demequina	4590	**0.01%**
d__Bacteria	k__unclassified_d__Bacteria	p__Proteobacteria	c__Gammaproteobacteria	o__Vibrionales	f__Vibrionaceae	g__Vibrio	4472	**0.01%**
d__Bacteria	k__unclassified_d__Bacteria	p__Thermotogae	c__Thermotogae	o__Petrotogales	f__unclassified_o__Petrotogales	g__Petrotoga	4410	**0.01%**
d__Bacteria	k__unclassified_d__Bacteria	p__Firmicutes	c__Clostridia	o__Clostridiales	f__Ruminococcaceae	g__Faecalibacterium	4376	**0.01%**
d__Bacteria	k__unclassified_d__Bacteria	p__Firmicutes	c__Bacilli	o__Bacillales	f__Bacillaceae	g__Oceanobacillus	4374	**0.01%**
d__Bacteria	k__unclassified_d__Bacteria	p__Firmicutes	c__Clostridia	o__Clostridiales	f__Peptococcaceae	g__Syntrophobotulus	4312	**0.01%**
d__Bacteria	k__unclassified_d__Bacteria	p__Cyanobacteria	c__unclassified_p__Cyanobacteria	o__Oscillatoriales	f__unclassified_o__Oscillatoriales	g__Planktothrix	4290	**0.01%**
d__Bacteria	k__unclassified_d__Bacteria	p__Proteobacteria	c__Gammaproteobacteria	o__Pseudomonadales	f__Pseudomonadaceae	g__Pseudomonas	4258	**0.01%**
d__Bacteria	k__unclassified_d__Bacteria	p__Firmicutes	c__Bacilli	o__Bacillales	f__Planococcaceae	g__unclassified_f__Planococcaceae	4126	**0.00%**
d__Archaea	k__unclassified_d__Archaea	p__Euryarchaeota	c__Methanococci	o__Methanococcales	f__Methanocaldococcaceae	g__Methanocaldococcus	4058	**0.00%**
d__Bacteria	k__unclassified_d__Bacteria	p__Firmicutes	c__Clostridia	o__Halanaerobiales	f__Halobacteroidaceae	g__unclassified_f__Halobacteroidaceae	3978	**0.00%**
d__Bacteria	k__unclassified_d__Bacteria	p__Firmicutes	c__Clostridia	o__Halanaerobiales	f__Halobacteroidaceae	g__Orenia	3816	**0.00%**
d__Bacteria	k__unclassified_d__Bacteria	p__Chloroflexi	c__Anaerolineae	o__Anaerolineales	f__Anaerolineaceae	g__Flexilinea	3792	**0.00%**
d__Bacteria	k__unclassified_d__Bacteria	p__Firmicutes	c__Negativicutes	o__Selenomonadales	f__Veillonellaceae	g__Propionispora	3686	**0.00%**
d__Bacteria	k__unclassified_d__Bacteria	p__Firmicutes	c__Erysipelotrichia	o__Erysipelotrichales	f__Erysipelotrichaceae	g__Allobaculum	3486	**0.00%**
d__Bacteria	k__unclassified_d__Bacteria	p__Firmicutes	c__Bacilli	o__Bacillales	f__Staphylococcaceae	g__Salinicoccus	3468	**0.00%**
d__Bacteria	k__unclassified_d__Bacteria	p__Firmicutes	c__Clostridia	o__Clostridiales	f__Ruminococcaceae	g__Mageeibacillus	3454	**0.00%**
d__Bacteria	k__unclassified_d__Bacteria	p__Firmicutes	c__Bacilli	o__Lactobacillales	f__Streptococcaceae	g__Streptococcus	3428	**0.00%**
d__Bacteria	k__unclassified_d__Bacteria	p__Firmicutes	c__Bacilli	o__Lactobacillales	f__Lactobacillaceae	g__Lactobacillus	3386	**0.00%**
d__Bacteria	k__unclassified_d__Bacteria	p__Candidatus_Yanofskybacteria	c__unclassified_p__Candidatus_Yanofskybacteria	o__unclassified_p__Candidatus_Yanofskybacteria	f__unclassified_p__Candidatus_Yanofskybacteria	g__unclassified_p__Candidatus_Yanofskybacteria	3378	**0.00%**
d__Bacteria	k__unclassified_d__Bacteria	p__Firmicutes	c__Tissierellia	o__Tissierellales	f__Peptoniphilaceae	g__unclassified_f__Peptoniphilaceae	3376	**0.00%**
d__Bacteria	k__unclassified_d__Bacteria	p__Firmicutes	c__Erysipelotrichia	o__Erysipelotrichales	f__Erysipelotrichaceae	g__Turicibacter	3182	**0.00%**
d__Archaea	k__unclassified_d__Archaea	p__Euryarchaeota	c__Methanococci	o__Methanococcales	f__Methanococcaceae	g__Methanococcus	3176	**0.00%**
d__Bacteria	k__unclassified_d__Bacteria	p__Proteobacteria	c__Gammaproteobacteria	o__unclassified_c__Gammaproteobacteria	f__unclassified_c__Gammaproteobacteria	g__unclassified_c__Gammaproteobacteria	2944	**0.00%**
d__Bacteria	k__unclassified_d__Bacteria	p__Chloroflexi	c__Ardenticatenia	o__Ardenticatenales	f__Ardenticatenaceae	g__Ardenticatena	2902	**0.00%**
d__Viruses	k__unclassified_d__Viruses	p__unclassified_d__Viruses	c__unclassified_d__Viruses	o__unclassified_d__Viruses	f__unclassified_d__Viruses	g__unclassified_d__Viruses	2866	**0.00%**
d__Archaea	k__unclassified_d__Archaea	p__Euryarchaeota	c__Methanobacteria	o__Methanobacteriales	f__Methanobacteriaceae	g__Methanobacterium	2850	**0.00%**
d__Bacteria	k__unclassified_d__Bacteria	p__Firmicutes	c__Negativicutes	o__Selenomonadales	f__Veillonellaceae	g__Anaerosporomusa	2760	**0.00%**
d__Bacteria	k__unclassified_d__Bacteria	p__Firmicutes	c__Clostridia	o__Clostridiales	f__Syntrophomonadaceae	g__Dethiobacter	2748	**0.00%**
d__Bacteria	k__unclassified_d__Bacteria	p__Firmicutes	c__Erysipelotrichia	o__Erysipelotrichales	f__Erysipelotrichaceae	g__unclassified_f__Erysipelotrichaceae	2738	**0.00%**
d__Bacteria	k__unclassified_d__Bacteria	p__Firmicutes	c__Bacilli	o__Bacillales	f__Thermoactinomycetaceae	g__Desmospora	2716	**0.00%**
d__Bacteria	k__unclassified_d__Bacteria	p__Firmicutes	c__Bacilli	o__Bacillales	f__Bacillaceae	g__Pontibacillus	2702	**0.00%**
d__Bacteria	k__unclassified_d__Bacteria	p__Firmicutes	c__Negativicutes	o__Selenomonadales	f__Veillonellaceae	g__Acetonema	2620	**0.00%**
d__Bacteria	k__unclassified_d__Bacteria	p__Firmicutes	c__Bacilli	o__Bacillales	f__Bacillaceae	g__Domibacillus	2606	**0.00%**
d__Eukaryota	k__Metazoa	p__Chordata	c__Mammalia	o__Primates	f__Hominidae	g__Homo	2602	**0.00%**
d__Bacteria	k__unclassified_d__Bacteria	p__Firmicutes	c__Negativicutes	o__Selenomonadales	f__Veillonellaceae	g__Selenomonas	2578	**0.00%**
d__Bacteria	k__unclassified_d__Bacteria	p__Proteobacteria	c__Gammaproteobacteria	o__Enterobacteriales	f__Enterobacteriaceae	g__Klebsiella	2578	**0.00%**
d__Bacteria	k__unclassified_d__Bacteria	p__Firmicutes	c__Clostridia	o__Clostridiales	f__unclassified_o__Clostridiales	g__Flavonifractor	2556	**0.00%**
d__Bacteria	k__unclassified_d__Bacteria	p__Firmicutes	c__Clostridia	o__Clostridiales	f__Catabacteriaceae	g__Catabacter	2534	**0.00%**
d__Archaea	k__unclassified_d__Archaea	p__Euryarchaeota	c__Methanomicrobia	o__Methanomicrobiales	f__unclassified_o__Methanomicrobiales	g__unclassified_o__Methanomicrobiales	2526	**0.00%**
d__Bacteria	k__unclassified_d__Bacteria	p__Firmicutes	c__Clostridia	o__Thermoanaerobacterales	f__Thermoanaerobacteraceae	g__unclassified_f__Thermoanaerobacteraceae	2516	**0.00%**
d__Bacteria	k__unclassified_d__Bacteria	p__Proteobacteria	c__Deltaproteobacteria	o__Myxococcales	f__Myxococcaceae	g__Corallococcus	2484	**0.00%**
d__Bacteria	k__unclassified_d__Bacteria	p__Proteobacteria	c__Deltaproteobacteria	o__unclassified_c__Deltaproteobacteria	f__unclassified_c__Deltaproteobacteria	g__unclassified_c__Deltaproteobacteria	2432	**0.00%**
d__Bacteria	k__unclassified_d__Bacteria	p__Firmicutes	c__Clostridia	o__Clostridiales	f__Lachnospiraceae	g__Oribacterium	2404	**0.00%**
d__Bacteria	k__unclassified_d__Bacteria	p__Proteobacteria	c__Deltaproteobacteria	o__Desulfovibrionales	f__Desulfovibrionaceae	g__Desulfovibrio	2372	**0.00%**
d__Bacteria	k__unclassified_d__Bacteria	p__Proteobacteria	c__Alphaproteobacteria	o__Sphingomonadales	f__Sphingomonadaceae	g__Sphingopyxis	2332	**0.00%**
d__Bacteria	k__unclassified_d__Bacteria	p__Bacteroidetes	c__Sphingobacteriia	o__Sphingobacteriales	f__Sphingobacteriaceae	g__Pedobacter	2330	**0.00%**
d__Bacteria	k__unclassified_d__Bacteria	p__Proteobacteria	c__Alphaproteobacteria	o__Rhizobiales	f__Rhizobiaceae	g__Rhizobium	2240	**0.00%**
d__Bacteria	k__unclassified_d__Bacteria	p__Nitrospirae	c__Nitrospira	o__Nitrospirales	f__Nitrospiraceae	g__Nitrospira	2238	**0.00%**
d__Bacteria	k__unclassified_d__Bacteria	p__Proteobacteria	c__Gammaproteobacteria	o__Pasteurellales	f__Pasteurellaceae	g__Actinobacillus	2236	**0.00%**
d__Bacteria	k__unclassified_d__Bacteria	p__Bacteroidetes	c__Bacteroidia	o__Bacteroidales	f__Bacteroidaceae	g__Bacteroides	2234	**0.00%**
d__Bacteria	k__unclassified_d__Bacteria	p__Firmicutes	c__Bacilli	o__Bacillales	f__Paenibacillaceae	g__Saccharibacillus	2210	**0.00%**
d__Bacteria	k__unclassified_d__Bacteria	p__Proteobacteria	c__Deltaproteobacteria	o__Desulfobacterales	f__Desulfobulbaceae	g__Desulfocapsa	2200	**0.00%**
d__Bacteria	k__unclassified_d__Bacteria	p__Firmicutes	c__Bacilli	o__Bacillales	f__Thermoactinomycetaceae	g__Thermoactinomyces	2186	**0.00%**
d__Bacteria	k__unclassified_d__Bacteria	p__Thermotogae	c__Thermotogae	o__Thermotogales	f__Thermotogaceae	g__Thermotoga	2182	**0.00%**
d__Bacteria	k__unclassified_d__Bacteria	p__Firmicutes	c__Negativicutes	o__Selenomonadales	f__Veillonellaceae	g__Anaeromusa	2156	**0.00%**
d__Bacteria	k__unclassified_d__Bacteria	p__Fusobacteria	c__Fusobacteriia	o__Fusobacteriales	f__Fusobacteriaceae	g__Fusobacterium	2128	**0.00%**
d__Bacteria	k__unclassified_d__Bacteria	p__Tenericutes	c__Mollicutes	o__Acholeplasmatales	f__Acholeplasmataceae	g__Acholeplasma	2118	**0.00%**
d__Bacteria	k__unclassified_d__Bacteria	p__Firmicutes	c__Bacilli	o__Bacillales	f__Bacillaceae	g__Terribacillus	2110	**0.00%**
d__Bacteria	k__unclassified_d__Bacteria	p__Cyanobacteria	c__unclassified_p__Cyanobacteria	o__Oscillatoriales	f__unclassified_o__Oscillatoriales	g__Pseudanabaena	2022	**0.00%**
d__Bacteria	k__unclassified_d__Bacteria	p__Cyanobacteria	c__unclassified_p__Cyanobacteria	o__Oscillatoriales	f__unclassified_o__Oscillatoriales	g__Coleofasciculus	1986	**0.00%**
d__Bacteria	k__unclassified_d__Bacteria	p__Firmicutes	c__Bacilli	o__Lactobacillales	f__Carnobacteriaceae	g__Trichococcus	1980	**0.00%**
d__Bacteria	k__unclassified_d__Bacteria	p__Firmicutes	c__Clostridia	o__Clostridiales	f__Clostridiaceae	g__Proteiniclasticum	1936	**0.00%**
d__Bacteria	k__unclassified_d__Bacteria	p__Firmicutes	c__Bacilli	o__Bacillales	f__Planococcaceae	g__Bhargavaea	1932	**0.00%**
d__Bacteria	k__unclassified_d__Bacteria	p__Firmicutes	c__Clostridia	o__Clostridiales	f__Peptostreptococcaceae	g__Terrisporobacter	1912	**0.00%**
d__Bacteria	k__unclassified_d__Bacteria	p__Proteobacteria	c__Gammaproteobacteria	o__Xanthomonadales	f__Rhodanobacteraceae	g__Rhodanobacter	1900	**0.00%**
d__Bacteria	k__unclassified_d__Bacteria	p__Fusobacteria	c__Fusobacteriia	o__Fusobacteriales	f__Leptotrichiaceae	g__Sebaldella	1878	**0.00%**
d__Bacteria	k__unclassified_d__Bacteria	p__Firmicutes	c__Clostridia	o__Clostridiales	f__Lachnospiraceae	g__Anaerostipes	1872	**0.00%**
d__Bacteria	k__unclassified_d__Bacteria	p__Firmicutes	c__Clostridia	o__Halanaerobiales	f__Halobacteroidaceae	g__Halobacteroides	1862	**0.00%**
d__Bacteria	k__unclassified_d__Bacteria	p__Fibrobacteres	c__Chitinivibrionia	o__unclassified_c__Chitinivibrionia	f__unclassified_c__Chitinivibrionia	g__unclassified_c__Chitinivibrionia	1862	**0.00%**
d__Bacteria	k__unclassified_d__Bacteria	p__Bacteroidetes	c__Flavobacteriia	o__Flavobacteriales	f__Flavobacteriaceae	g__Capnocytophaga	1860	**0.00%**
d__Bacteria	k__unclassified_d__Bacteria	p__Nitrospirae	c__Nitrospira	o__unclassified_c__Nitrospira	f__unclassified_c__Nitrospira	g__unclassified_c__Nitrospira	1840	**0.00%**
d__Bacteria	k__unclassified_d__Bacteria	p__Bacteroidetes	c__Bacteroidia	o__Bacteroidales	f__Rikenellaceae	g__Alistipes	1836	**0.00%**
d__Bacteria	k__unclassified_d__Bacteria	p__Firmicutes	c__Bacilli	o__Bacillales	f__Staphylococcaceae	g__Staphylococcus	1834	**0.00%**
d__Viruses	k__unclassified_d__Viruses	p__unclassified_d__Viruses	c__unclassified_d__Viruses	o__Caudovirales	f__Myoviridae	g__Spounalikevirus	1824	**0.00%**
d__Bacteria	k__unclassified_d__Bacteria	p__Firmicutes	c__Bacilli	o__Bacillales	f__Thermoactinomycetaceae	g__Risungbinella	1808	**0.00%**
d__Bacteria	k__unclassified_d__Bacteria	p__Proteobacteria	c__Gammaproteobacteria	o__Pasteurellales	f__Pasteurellaceae	g__Bibersteinia	1794	**0.00%**
d__Bacteria	k__unclassified_d__Bacteria	p__Chlamydiae	c__Chlamydiia	o__Chlamydiales	f__Chlamydiaceae	g__Chlamydia	1790	**0.00%**
d__Bacteria	k__unclassified_d__Bacteria	p__Proteobacteria	c__Gammaproteobacteria	o__Pseudomonadales	f__Moraxellaceae	g__Acinetobacter	1788	**0.00%**
d__Bacteria	k__unclassified_d__Bacteria	p__Firmicutes	c__Bacilli	o__Bacillales	f__Bacillaceae	g__Salimicrobium	1780	**0.00%**
d__Bacteria	k__unclassified_d__Bacteria	p__unclassified_d__Bacteria	c__unclassified_d__Bacteria	o__unclassified_d__Bacteria	f__unclassified_d__Bacteria	g__Thermobaculum	1758	**0.00%**
d__Viruses	k__unclassified_d__Viruses	p__unclassified_d__Viruses	c__unclassified_d__Viruses	o__unclassified_d__Viruses	f__Phycodnaviridae	g__Chlorovirus	1750	**0.00%**
d__Viruses	k__unclassified_d__Viruses	p__unclassified_d__Viruses	c__unclassified_d__Viruses	o__unclassified_d__Viruses	f__Tectiviridae	g__Tectivirus	1734	**0.00%**
d__Archaea	k__unclassified_d__Archaea	p__unclassified_d__Archaea	c__unclassified_d__Archaea	o__unclassified_d__Archaea	f__unclassified_d__Archaea	g__unclassified_d__Archaea	1690	**0.00%**
d__Bacteria	k__unclassified_d__Bacteria	p__Chloroflexi	c__Anaerolineae	o__Anaerolineales	f__Anaerolineaceae	g__Longilinea	1678	**0.00%**
d__Bacteria	k__unclassified_d__Bacteria	p__Firmicutes	c__Bacilli	o__Bacillales	f__Planococcaceae	g__Planococcus	1658	**0.00%**
d__Bacteria	k__unclassified_d__Bacteria	p__Firmicutes	c__Bacilli	o__Bacillales	f__Bacillaceae	g__Gracilibacillus	1646	**0.00%**
d__Bacteria	k__unclassified_d__Bacteria	p__Aquificae	c__Aquificae	o__Aquificales	f__Hydrogenothermaceae	g__Sulfurihydrogenibium	1638	**0.00%**
d__Bacteria	k__unclassified_d__Bacteria	p__Proteobacteria	c__Alphaproteobacteria	o__Rhizobiales	f__Methylobacteriaceae	g__Methylobacterium	1628	**0.00%**
d__Bacteria	k__unclassified_d__Bacteria	p__Firmicutes	c__Clostridia	o__Thermoanaerobacterales	f__Thermoanaerobacteraceae	g__Ammonifex	1610	**0.00%**
d__Archaea	k__unclassified_d__Archaea	p__Euryarchaeota	c__Methanomicrobia	o__Methanosarcinales	f__Candidatus_Methanoperedenaceae	g__Candidatus_Methanoperedens	1592	**0.00%**
d__Archaea	k__unclassified_d__Archaea	p__Thaumarchaeota	c__unclassified_p__Thaumarchaeota	o__Nitrosopumilales	f__Nitrosopumilaceae	g__Nitrosopumilus	1582	**0.00%**
d__Bacteria	k__unclassified_d__Bacteria	p__Firmicutes	c__Bacilli	o__Bacillales	f__Bacillaceae	g__Paucisalibacillus	1580	**0.00%**
d__Bacteria	k__unclassified_d__Bacteria	p__unclassified_d__Bacteria	c__unclassified_d__Bacteria	o__Haloplasmatales	f__Haloplasmataceae	g__Haloplasma	1570	**0.00%**
d__Bacteria	k__unclassified_d__Bacteria	p__Actinobacteria	c__Coriobacteriia	o__Coriobacteriales	f__Coriobacteriaceae	g__Collinsella	1550	**0.00%**
d__Archaea	k__unclassified_d__Archaea	p__Euryarchaeota	c__Methanomicrobia	o__Methanosarcinales	f__Methanosaetaceae	g__Methanosaeta	1546	**0.00%**
d__Bacteria	k__unclassified_d__Bacteria	p__Firmicutes	c__Bacilli	o__Bacillales	f__Bacillaceae	g__Lentibacillus	1542	**0.00%**
d__Bacteria	k__unclassified_d__Bacteria	p__Firmicutes	c__Bacilli	o__Bacillales	f__Bacillaceae	g__Salinibacillus	1518	**0.00%**
d__Bacteria	k__unclassified_d__Bacteria	p__Deinococcus-Thermus	c__Deinococci	o__Deinococcales	f__Trueperaceae	g__Truepera	1512	**0.00%**
d__Bacteria	k__unclassified_d__Bacteria	p__Cyanobacteria	c__unclassified_p__Cyanobacteria	o__Oscillatoriales	f__unclassified_o__Oscillatoriales	g__Trichodesmium	1486	**0.00%**
d__Bacteria	k__unclassified_d__Bacteria	p__Deinococcus-Thermus	c__Deinococci	o__Deinococcales	f__Deinococcaceae	g__Deinococcus	1462	**0.00%**
d__Bacteria	k__unclassified_d__Bacteria	p__Chlorobi	c__Chlorobia	o__Chlorobiales	f__Chlorobiaceae	g__Chlorobium	1460	**0.00%**
d__Bacteria	k__unclassified_d__Bacteria	p__Firmicutes	c__Bacilli	o__Bacillales	f__Listeriaceae	g__Listeria	1448	**0.00%**
d__Bacteria	k__unclassified_d__Bacteria	p__Firmicutes	c__Negativicutes	o__Selenomonadales	f__Veillonellaceae	g__Veillonella	1426	**0.00%**
d__Bacteria	k__unclassified_d__Bacteria	p__Thermotogae	c__Thermotogae	o__Kosmotogales	f__Kosmotogaceae	g__Mesotoga	1418	**0.00%**
d__Bacteria	k__unclassified_d__Bacteria	p__Firmicutes	c__Bacilli	o__Bacillales	f__Bacillaceae	g__Halobacillus	1404	**0.00%**
d__Bacteria	k__unclassified_d__Bacteria	p__Firmicutes	c__Bacilli	o__Bacillales	f__Planococcaceae	g__Paenisporosarcina	1378	**0.00%**
d__Bacteria	k__unclassified_d__Bacteria	p__Firmicutes	c__Bacilli	o__Lactobacillales	f__Carnobacteriaceae	g__Alkalibacterium	1362	**0.00%**
d__Bacteria	k__unclassified_d__Bacteria	p__candidate_division_CPR2	c__unclassified_p__candidate_division_CPR2	o__unclassified_p__candidate_division_CPR2	f__unclassified_p__candidate_division_CPR2	g__unclassified_p__candidate_division_CPR2	1350	**0.00%**
d__Bacteria	k__unclassified_d__Bacteria	p__Firmicutes	c__Clostridia	o__Halanaerobiales	f__Halobacteroidaceae	g__Halonatronum	1344	**0.00%**
d__Bacteria	k__unclassified_d__Bacteria	p__Proteobacteria	c__Alphaproteobacteria	o__Sphingomonadales	f__Erythrobacteraceae	g__Erythrobacter	1312	**0.00%**
d__Bacteria	k__unclassified_d__Bacteria	p__Proteobacteria	c__Alphaproteobacteria	o__Rhodospirillales	f__Acetobacteraceae	g__Acidiphilium	1304	**0.00%**
d__Bacteria	k__unclassified_d__Bacteria	p__Firmicutes	c__Bacilli	o__Lactobacillales	f__Carnobacteriaceae	g__Jeotgalibaca	1302	**0.00%**
d__Bacteria	k__unclassified_d__Bacteria	p__candidate_division_NC10	c__unclassified_p__candidate_division_NC10	o__unclassified_p__candidate_division_NC10	f__unclassified_p__candidate_division_NC10	g__Candidatus_Methylomirabilis	1250	**0.00%**
d__Bacteria	k__unclassified_d__Bacteria	p__Cyanobacteria	c__unclassified_p__Cyanobacteria	o__Oscillatoriales	f__unclassified_o__Oscillatoriales	g__Geitlerinema	1230	**0.00%**
d__Bacteria	k__unclassified_d__Bacteria	p__Firmicutes	c__Clostridia	o__Clostridiales	f__Peptostreptococcaceae	g__unclassified_f__Peptostreptococcaceae	1216	**0.00%**
d__Bacteria	k__unclassified_d__Bacteria	p__Firmicutes	c__Erysipelotrichia	o__Erysipelotrichales	f__Erysipelotrichaceae	g__Erysipelothrix	1210	**0.00%**
d__Bacteria	k__unclassified_d__Bacteria	p__Proteobacteria	c__Alphaproteobacteria	o__Rhodobacterales	f__Rhodobacteraceae	g__Paracoccus	1208	**0.00%**
d__Bacteria	k__unclassified_d__Bacteria	p__Firmicutes	c__Clostridia	o__Natranaerobiales	f__Natranaerobiaceae	g__Natranaerobius	1172	**0.00%**
d__Bacteria	k__unclassified_d__Bacteria	p__Synergistetes	c__Synergistia	o__Synergistales	f__Synergistaceae	g__unclassified_f__Synergistaceae	1170	**0.00%**
d__Bacteria	k__unclassified_d__Bacteria	p__Proteobacteria	c__Gammaproteobacteria	o__Chromatiales	f__Ectothiorhodospiraceae	g__Thioalkalivibrio	1150	**0.00%**
d__Archaea	k__unclassified_d__Archaea	p__Candidatus_Bathyarchaeota	c__unclassified_p__Candidatus_Bathyarchaeota	o__unclassified_p__Candidatus_Bathyarchaeota	f__unclassified_p__Candidatus_Bathyarchaeota	g__unclassified_p__Candidatus_Bathyarchaeota	1142	**0.00%**
d__Bacteria	k__unclassified_d__Bacteria	p__Firmicutes	c__Clostridia	o__Clostridiales	f__Christensenellaceae	g__Christensenella	1128	**0.00%**
d__Bacteria	k__unclassified_d__Bacteria	p__Chloroflexi	c__Caldilineae	o__Caldilineales	f__Caldilineaceae	g__Caldilinea	1118	**0.00%**
d__Bacteria	k__unclassified_d__Bacteria	p__Bacteroidetes	c__Sphingobacteriia	o__Sphingobacteriales	f__Saprospiraceae	g__Aureispira	1108	**0.00%**
d__Bacteria	k__unclassified_d__Bacteria	p__Bacteroidetes	c__Bacteroidia	o__Bacteroidales	f__Porphyromonadaceae	g__Odoribacter	1088	**0.00%**
d__Bacteria	k__unclassified_d__Bacteria	p__Candidatus_Amesbacteria	c__unclassified_p__Candidatus_Amesbacteria	o__unclassified_p__Candidatus_Amesbacteria	f__unclassified_p__Candidatus_Amesbacteria	g__unclassified_p__Candidatus_Amesbacteria	1084	**0.00%**
d__Bacteria	k__unclassified_d__Bacteria	p__Proteobacteria	c__Alphaproteobacteria	o__Rhizobiales	f__Bartonellaceae	g__Bartonella	1074	**0.00%**
d__Bacteria	k__unclassified_d__Bacteria	p__Acidobacteria	c__Acidobacteriia	o__Acidobacteriales	f__Acidobacteriaceae	g__Acidobacterium	1062	**0.00%**
d__Bacteria	k__unclassified_d__Bacteria	p__Chloroflexi	c__Thermomicrobia	o__Sphaerobacterales	f__Sphaerobacteraceae	g__Nitrolancea	1060	**0.00%**
d__Bacteria	k__unclassified_d__Bacteria	p__Cyanobacteria	c__unclassified_p__Cyanobacteria	o__Nostocales	f__Nostocaceae	g__Nostoc	1054	**0.00%**
d__Bacteria	k__unclassified_d__Bacteria	p__Candidatus_Atribacteria	c__unclassified_p__Candidatus_Atribacteria	o__unclassified_p__Candidatus_Atribacteria	f__unclassified_p__Candidatus_Atribacteria	g__unclassified_p__Candidatus_Atribacteria	1044	**0.00%**
d__Bacteria	k__unclassified_d__Bacteria	p__Firmicutes	c__Bacilli	o__Bacillales	f__unclassified_o__Bacillales	g__Acidibacillus	1026	**0.00%**
d__Bacteria	k__unclassified_d__Bacteria	p__Synergistetes	c__Synergistia	o__Synergistales	f__Synergistaceae	g__Thermovirga	1012	**0.00%**
d__Bacteria	k__unclassified_d__Bacteria	p__Tenericutes	c__Mollicutes	o__Mycoplasmatales	f__Mycoplasmataceae	g__Mycoplasma	1008	**0.00%**
d__Bacteria	k__unclassified_d__Bacteria	p__Thermotogae	c__Thermotogae	o__Thermotogales	f__Thermotogaceae	g__Pseudothermotoga	992	**0.00%**
d__Bacteria	k__unclassified_d__Bacteria	p__Proteobacteria	c__Betaproteobacteria	o__Nitrosomonadales	f__Nitrosomonadaceae	g__Nitrosomonas	972	**0.00%**
d__unclassified	k__unclassified_d__unclassified	p__unclassified_d__unclassified	c__unclassified_d__unclassified	o__unclassified_d__unclassified	f__unclassified_d__unclassified	g__unclassified_d__unclassified	968	**0.00%**
d__Bacteria	k__unclassified_d__Bacteria	p__Acidobacteria	c__Holophagae	o__Holophagales	f__Holophagaceae	g__Holophaga	968	**0.00%**
d__Bacteria	k__unclassified_d__Bacteria	p__Chlorobi	c__unclassified_p__Chlorobi	o__unclassified_p__Chlorobi	f__unclassified_p__Chlorobi	g__unclassified_p__Chlorobi	960	**0.00%**
d__Bacteria	k__unclassified_d__Bacteria	p__Synergistetes	c__Synergistia	o__Synergistales	f__Synergistaceae	g__Pyramidobacter	948	**0.00%**
d__Bacteria	k__unclassified_d__Bacteria	p__Firmicutes	c__Bacilli	o__Bacillales	f__unclassified_o__Bacillales	g__Exiguobacterium	946	**0.00%**
d__Bacteria	k__unclassified_d__Bacteria	p__Cyanobacteria	c__unclassified_p__Cyanobacteria	o__Pleurocapsales	f__unclassified_o__Pleurocapsales	g__Myxosarcina	930	**0.00%**
d__Bacteria	k__unclassified_d__Bacteria	p__Candidatus_Dadabacteria	c__unclassified_p__Candidatus_Dadabacteria	o__unclassified_p__Candidatus_Dadabacteria	f__unclassified_p__Candidatus_Dadabacteria	g__unclassified_p__Candidatus_Dadabacteria	918	**0.00%**
d__Bacteria	k__unclassified_d__Bacteria	p__Firmicutes	c__Bacilli	o__Bacillales	f__Bacillaceae	g__Sediminibacillus	914	**0.00%**
d__Bacteria	k__unclassified_d__Bacteria	p__Bacteroidetes	c__Flavobacteriia	o__unclassified_c__Flavobacteriia	f__unclassified_c__Flavobacteriia	g__unclassified_c__Flavobacteriia	896	**0.00%**

d__Bacteria	k__unclassified_d__Bacteria	p__Proteobacteria	c__Deltaproteobacteria	o__Desulfobacterales	f__Desulfobulbaceae	g__Desulfurivibrio	896	**0.00%**
d__Bacteria	k__unclassified_d__Bacteria	p__Proteobacteria	c__Gammaproteobacteria	o__Methylococcales	f__Methylococcaceae	g__Methylobacter	890	**0.00%**
d__Bacteria	k__unclassified_d__Bacteria	p__Firmicutes	c__Bacilli	o__Lactobacillales	f__Aerococcaceae	g__Aerococcus	884	**0.00%**
d__Bacteria	k__unclassified_d__Bacteria	p__Elusimicrobia	c__Elusimicrobia	o__Elusimicrobiales	f__Elusimicrobiaceae	g__Elusimicrobium	882	**0.00%**
d__Bacteria	k__unclassified_d__Bacteria	p__Cyanobacteria	c__unclassified_p__Cyanobacteria	o__Chroococcales	f__unclassified_o__Chroococcales	g__Halothece	872	**0.00%**
d__Bacteria	k__unclassified_d__Bacteria	p__Bacteroidetes	c__Bacteroidia	o__Bacteroidales	f__Porphyromonadaceae	g__Dysgonomonas	858	**0.00%**
d__Bacteria	k__unclassified_d__Bacteria	p__Spirochaetes	c__Spirochaetia	o__Brachyspirales	f__Brachyspiraceae	g__Brachyspira	844	**0.00%**
d__Bacteria	k__unclassified_d__Bacteria	p__Proteobacteria	c__Gammaproteobacteria	o__Enterobacteriales	f__Enterobacteriaceae	g__Pectobacterium	844	**0.00%**
d__Bacteria	k__unclassified_d__Bacteria	p__Firmicutes	c__Clostridia	o__Clostridiales	f__Lachnospiraceae	g__Pseudobutyrivibrio	840	**0.00%**
d__Bacteria	k__unclassified_d__Bacteria	p__Proteobacteria	c__Deltaproteobacteria	o__Desulfobacterales	f__Desulfobacteraceae	g__Desulfospira	830	**0.00%**
d__Bacteria	k__unclassified_d__Bacteria	p__Aquificae	c__Aquificae	o__Aquificales	f__unclassified_o__Aquificales	g__Thermosulfidibacter	824	**0.00%**
d__Bacteria	k__unclassified_d__Bacteria	p__Chloroflexi	c__Anaerolineae	o__Anaerolineales	f__Anaerolineaceae	g__Leptolinea	806	**0.00%**
d__Bacteria	k__unclassified_d__Bacteria	p__Proteobacteria	c__Alphaproteobacteria	o__Rhizobiales	f__Rhizobiaceae	g__Sinorhizobium	804	**0.00%**
d__Bacteria	k__unclassified_d__Bacteria	p__Firmicutes	c__Bacilli	o__Bacillales	f__Alicyclobacillaceae	g__Kyrpidia	804	**0.00%**
d__Bacteria	k__unclassified_d__Bacteria	p__Actinobacteria	c__Actinobacteria	o__Micrococcales	f__Micrococcaceae	g__Kocuria	792	**0.00%**
d__Bacteria	k__unclassified_d__Bacteria	p__Proteobacteria	c__Epsilonproteobacteria	o__Campylobacterales	f__Helicobacteraceae	g__Sulfuricurvum	784	**0.00%**
d__Bacteria	k__unclassified_d__Bacteria	p__Actinobacteria	c__Actinobacteria	o__Geodermatophilales	f__Geodermatophilaceae	g__Modestobacter	782	**0.00%**
d__Bacteria	k__unclassified_d__Bacteria	p__Actinobacteria	c__Coriobacteriia	o__Coriobacteriales	f__Atopobiaceae	g__Olsenella	780	**0.00%**
d__Bacteria	k__unclassified_d__Bacteria	p__candidate_division_WOR-3	c__unclassified_p__candidate_division_WOR-3	o__unclassified_p__candidate_division_WOR-3	f__unclassified_p__candidate_division_WOR-3	g__unclassified_p__candidate_division_WOR-3	774	**0.00%**
d__Bacteria	k__unclassified_d__Bacteria	p__Proteobacteria	c__Alphaproteobacteria	o__Magnetococcales	f__Magnetococcaceae	g__Magnetococcus	764	**0.00%**
d__Bacteria	k__unclassified_d__Bacteria	p__Proteobacteria	c__Deltaproteobacteria	o__Myxococcales	f__Myxococcaceae	g__Myxococcus	762	**0.00%**
d__Bacteria	k__unclassified_d__Bacteria	p__Chloroflexi	c__Chloroflexia	o__Chloroflexales	f__Chloroflexaceae	g__Chloroflexus	752	**0.00%**
d__Archaea	k__unclassified_d__Archaea	p__Euryarchaeota	c__Methanomicrobia	o__Methanomicrobiales	f__Methanomicrobiaceae	g__Methanoculleus	752	**0.00%**
d__Bacteria	k__unclassified_d__Bacteria	p__Candidatus_Daviesbacteria	c__unclassified_p__Candidatus_Daviesbacteria	o__unclassified_p__Candidatus_Daviesbacteria	f__unclassified_p__Candidatus_Daviesbacteria	g__unclassified_p__Candidatus_Daviesbacteria	748	**0.00%**
d__Bacteria	k__unclassified_d__Bacteria	p__Proteobacteria	c__Deltaproteobacteria	o__Desulfuromonadales	f__Geobacteraceae	g__Geobacter	748	**0.00%**
d__Bacteria	k__unclassified_d__Bacteria	p__Bacteroidetes	c__Cytophagia	o__Cytophagales	f__Flammeovirgaceae	g__Roseivirga	730	**0.00%**
d__Bacteria	k__unclassified_d__Bacteria	p__Firmicutes	c__Negativicutes	o__Selenomonadales	f__Veillonellaceae	g__Dialister	730	**0.00%**
d__Bacteria	k__unclassified_d__Bacteria	p__Chloroflexi	c__Chloroflexia	o__Chloroflexales	f__Roseiflexaceae	g__Roseiflexus	724	**0.00%**
d__Bacteria	k__unclassified_d__Bacteria	p__Synergistetes	c__Synergistia	o__Synergistales	f__Synergistaceae	g__Aminiphilus	718	**0.00%**
d__Bacteria	k__unclassified_d__Bacteria	p__Cyanobacteria	c__unclassified_p__Cyanobacteria	o__Nostocales	f__Nostocaceae	g__Nodularia	704	**0.00%**
d__Bacteria	k__unclassified_d__Bacteria	p__Proteobacteria	c__Betaproteobacteria	o__Burkholderiales	f__Oxalobacteraceae	g__Herbaspirillum	690	**0.00%**
d__Bacteria	k__unclassified_d__Bacteria	p__Candidatus_Marinimicrobia	c__unclassified_p__Candidatus_Marinimicrobia	o__unclassified_p__Candidatus_Marinimicrobia	f__unclassified_p__Candidatus_Marinimicrobia	g__unclassified_p__Candidatus_Marinimicrobia	672	**0.00%**
d__Bacteria	k__unclassified_d__Bacteria	p__Bacteroidetes	c__Sphingobacteriia	o__Sphingobacteriales	f__Chitinophagaceae	g__Segetibacter	670	**0.00%**
d__Bacteria	k__unclassified_d__Bacteria	p__Synergistetes	c__Synergistia	o__Synergistales	f__Synergistaceae	g__Cloacibacillus	654	**0.00%**
d__Bacteria	k__unclassified_d__Bacteria	p__Bacteroidetes	c__Sphingobacteriia	o__Sphingobacteriales	f__Sphingobacteriaceae	g__Mucilaginibacter	648	**0.00%**
d__Archaea	k__unclassified_d__Archaea	p__Euryarchaeota	c__Methanomicrobia	o__Methanomicrobiales	f__Methanomicrobiaceae	g__Methanolacinia	644	**0.00%**
d__Bacteria	k__unclassified_d__Bacteria	p__Synergistetes	c__Synergistia	o__Synergistales	f__Synergistaceae	g__Aminomonas	642	**0.00%**
d__Bacteria	k__unclassified_d__Bacteria	p__Fusobacteria	c__Fusobacteriia	o__Fusobacteriales	f__Leptotrichiaceae	g__Leptotrichia	638	**0.00%**
d__Bacteria	k__unclassified_d__Bacteria	p__Proteobacteria	c__Gammaproteobacteria	o__Enterobacteriales	f__Enterobacteriaceae	g__Providencia	636	**0.00%**
d__Bacteria	k__unclassified_d__Bacteria	p__Firmicutes	c__Bacilli	o__unclassified_c__Bacilli	f__unclassified_c__Bacilli	g__unclassified_c__Bacilli	634	**0.00%**
d__Bacteria	k__unclassified_d__Bacteria	p__Firmicutes	c__Bacilli	o__Bacillales	f__Thermoactinomycetaceae	g__Shimazuella	630	**0.00%**
d__Bacteria	k__unclassified_d__Bacteria	p__Cyanobacteria	c__unclassified_p__Cyanobacteria	o__Oscillatoriales	f__unclassified_o__Oscillatoriales	g__Arthrospira	628	**0.00%**
d__Bacteria	k__unclassified_d__Bacteria	p__Proteobacteria	c__Gammaproteobacteria	o__Alteromonadales	f__Colwelliaceae	g__Colwellia	620	**0.00%**
d__Bacteria	k__unclassified_d__Bacteria	p__Proteobacteria	c__Deltaproteobacteria	o__Desulfobacterales	f__Desulfobulbaceae	g__Desulfobulbus	618	**0.00%**
d__Archaea	k__unclassified_d__Archaea	p__Euryarchaeota	c__Archaeoglobi	o__Archaeoglobales	f__Archaeoglobaceae	g__Ferroglobus	616	**0.00%**
d__Bacteria	k__unclassified_d__Bacteria	p__Acidobacteria	c__Blastocatellia	o__unclassified_c__Blastocatellia	f__unclassified_c__Blastocatellia	g__Pyrinomonas	612	**0.00%**
d__Bacteria	k__unclassified_d__Bacteria	p__Gemmatimonadetes	c__Gemmatimonadetes	o__Gemmatimonadales	f__Gemmatimonadaceae	g__Gemmatimonas	606	**0.00%**
d__Bacteria	k__unclassified_d__Bacteria	p__Proteobacteria	c__Deltaproteobacteria	o__Desulfarculales	f__Desulfarculaceae	g__Dethiosulfatarculus	604	**0.00%**
d__Bacteria	k__unclassified_d__Bacteria	p__Proteobacteria	c__Gammaproteobacteria	o__Enterobacteriales	f__Enterobacteriaceae	g__Salmonella	602	**0.00%**
d__Bacteria	k__unclassified_d__Bacteria	p__Cyanobacteria	c__unclassified_p__Cyanobacteria	o__Oscillatoriales	f__unclassified_o__Oscillatoriales	g__Kamptonema	602	**0.00%**
d__Bacteria	k__unclassified_d__Bacteria	p__Chloroflexi	c__Ktedonobacteria	o__Ktedonobacterales	f__Ktedonobacteraceae	g__Ktedonobacter	598	**0.00%**
d__Bacteria	k__unclassified_d__Bacteria	p__Proteobacteria	c__Gammaproteobacteria	o__Aeromonadales	f__Succinivibrionaceae	g__Anaerobiospirillum	598	**0.00%**
d__Bacteria	k__unclassified_d__Bacteria	p__Firmicutes	c__Erysipelotrichia	o__Erysipelotrichales	f__Erysipelotrichaceae	g__Coprobacillus	598	**0.00%**
d__Bacteria	k__unclassified_d__Bacteria	p__Candidatus_Giovannonibacteri	c__unclassified_p__Candidatus_Giovannonibacteria	o__unclassified_p__Candidatus_Giovannonibacteria	f__unclassified_p__Candidatus_Giovannonibacteria	g__unclassified_p__Candidatus_Giovannonibacteria	590	**0.00%**
d__Bacteria	k__unclassified_d__Bacteria	p__Cyanobacteria	c__unclassified_p__Cyanobacteria	o__Oscillatoriales	f__unclassified_o__Oscillatoriales	g__Leptolyngbya	588	**0.00%**
d__Bacteria	k__unclassified_d__Bacteria	p__Proteobacteria	c__Betaproteobacteria	o__Burkholderiales	f__Comamonadaceae	g__Polaromonas	582	**0.00%**
d__Bacteria	k__unclassified_d__Bacteria	p__Proteobacteria	c__Alphaproteobacteria	o__Sphingomonadales	f__unclassified_o__Sphingomonadales	g__unclassified_o__Sphingomonadales	580	**0.00%**
d__Bacteria	k__unclassified_d__Bacteria	p__Actinobacteria	c__Thermoleophilia	o__Solirubrobacterales	f__unclassified_o__Solirubrobacterales	g__unclassified_o__Solirubrobacterales	578	**0.00%**
d__Bacteria	k__unclassified_d__Bacteria	p__Chloroflexi	c__Anaerolineae	o__Anaerolineales	f__Anaerolineaceae	g__Ornatilinea	568	**0.00%**
d__Bacteria	k__unclassified_d__Bacteria	p__Firmicutes	c__Bacilli	o__Bacillales	f__unclassified_o__Bacillales	g__Gemella	566	**0.00%**
d__Bacteria	k__unclassified_d__Bacteria	p__Firmicutes	c__Tissierellia	o__Tissierellales	f__Peptoniphilaceae	g__Anaerococcus	556	**0.00%**
d__Bacteria	k__unclassified_d__Bacteria	p__Firmicutes	c__Bacilli	o__Bacillales	f__Planococcaceae	g__Jeotgalibacillus	554	**0.00%**
d__Archaea	k__unclassified_d__Archaea	p__Euryarchaeota	c__Thermoplasmata	o__unclassified_c__Thermoplasmata	f__unclassified_c__Thermoplasmata	g__unclassified_c__Thermoplasmata	550	**0.00%**
d__Bacteria	k__unclassified_d__Bacteria	p__Actinobacteria	c__Coriobacteriia	o__Coriobacteriales	f__Atopobiaceae	g__Atopobium	534	**0.00%**
d__Bacteria	k__unclassified_d__Bacteria	p__Thermotogae	c__Thermotogae	o__Thermotogales	f__Fervidobacteriaceae	g__Thermosipho	526	**0.00%**
d__Bacteria	k__unclassified_d__Bacteria	p__Candidatus_Parcubacteria	c__unclassified_p__Candidatus_Parcubacteria	o__unclassified_p__Candidatus_Parcubacteria	f__unclassified_p__Candidatus_Parcubacteria	g__unclassified_p__Candidatus_Parcubacteria	524	**0.00%**
d__Bacteria	k__unclassified_d__Bacteria	p__Proteobacteria	c__Gammaproteobacteria	o__Aeromonadales	f__Succinivibrionaceae	g__Succinatimonas	510	**0.00%**
d__Bacteria	k__unclassified_d__Bacteria	p__Cyanobacteria	c__unclassified_p__Cyanobacteria	o__Nostocales	f__Rivulariaceae	g__Calothrix	508	**0.00%**
d__Bacteria	k__unclassified_d__Bacteria	p__Chloroflexi	c__Anaerolineae	o__Anaerolineales	f__Anaerolineaceae	g__Anaerolinea	504	**0.00%**
d__Bacteria	k__unclassified_d__Bacteria	p__Bacteroidetes	c__Flavobacteriia	o__Flavobacteriales	f__Flavobacteriaceae	g__Olleya	500	**0.00%**
d__Bacteria	k__unclassified_d__Bacteria	p__Deferribacteres	c__Deferribacteres	o__Deferribacterales	f__Deferribacteraceae	g__Deferribacter	500	**0.00%**
d__Bacteria	k__unclassified_d__Bacteria	p__Proteobacteria	c__Gammaproteobacteria	o__Enterobacteriales	f__Enterobacteriaceae	g__Serratia	494	**0.00%**
d__Bacteria	k__unclassified_d__Bacteria	p__Bacteroidetes	c__Flavobacteriia	o__Flavobacteriales	f__Cryomorphaceae	g__unclassified_f__Cryomorphaceae	484	**0.00%**
d__Bacteria	k__unclassified_d__Bacteria	p__Planctomycetes	c__Planctomycetia	o__Planctomycetales	f__Planctomycetaceae	g__Gemmata	474	**0.00%**
d__Bacteria	k__unclassified_d__Bacteria	p__Deinococcus-Thermus	c__Deinococci	o__Thermales	f__Thermaceae	g__Meiothermus	472	**0.00%**
d__Bacteria	k__unclassified_d__Bacteria	p__Proteobacteria	c__Deltaproteobacteria	o__Desulfobacterales	f__Desulfobacteraceae	g__Desulfobacterium	470	**0.00%**
d__Bacteria	k__unclassified_d__Bacteria	p__Bacteroidetes	c__Flavobacteriia	o__Flavobacteriales	f__Flavobacteriaceae	g__Flavobacterium	464	**0.00%**
d__Bacteria	k__unclassified_d__Bacteria	p__Bacteroidetes	c__Cytophagia	o__Cytophagales	f__Cytophagaceae	g__Flexibacter	464	**0.00%**
d__Bacteria	k__unclassified_d__Bacteria	p__Proteobacteria	c__Alphaproteobacteria	o__Rhizobiales	f__Bradyrhizobiaceae	g__Bradyrhizobium	458	**0.00%**
d__Bacteria	k__unclassified_d__Bacteria	p__Actinobacteria	c__Actinobacteria	o__Pseudonocardiales	f__Pseudonocardiaceae	g__Saccharothrix	446	**0.00%**
d__Bacteria	k__unclassified_d__Bacteria	p__Firmicutes	c__Clostridia	o__Clostridiales	f__Ruminococcaceae	g__Candidatus_Soleaferrea	442	**0.00%**
d__Bacteria	k__unclassified_d__Bacteria	p__Planctomycetes	c__Planctomycetia	o__Planctomycetales	f__Planctomycetaceae	g__Pirellula	438	**0.00%**
d__Viruses	k__unclassified_d__Viruses	p__unclassified_d__Viruses	c__unclassified_d__Viruses	o__Caudovirales	f__Myoviridae	g__T4likevirus	422	**0.00%**
d__Bacteria	k__unclassified_d__Bacteria	p__Planctomycetes	c__Planctomycetia	o__Candidatus_Brocadiales	f__Candidatus_Brocadiaceae	g__Candidatus_Kuenenia	420	**0.00%**
d__Archaea	k__unclassified_d__Archaea	p__Euryarchaeota	c__Methanobacteria	o__Methanobacteriales	f__Methanobacteriaceae	g__Methanothermobacter	416	**0.00%**
d__Bacteria	k__unclassified_d__Bacteria	p__Thermodesulfobacteria	c__Thermodesulfobacteria	o__Thermodesulfobacteriales	f__Thermodesulfobacteriaceae	g__Thermodesulfobacterium	408	**0.00%**
d__Bacteria	k__unclassified_d__Bacteria	p__Bacteroidetes	c__Cytophagia	o__Cytophagales	f__Cytophagaceae	g__Rufibacter	406	**0.00%**
d__Bacteria	k__unclassified_d__Bacteria	p__Firmicutes	c__Clostridia	o__Halanaerobiales	f__Halobacteroidaceae	g__Acetohalobium	394	**0.00%**
d__Bacteria	k__unclassified_d__Bacteria	p__Bacteroidetes	c__Flavobacteriia	o__Flavobacteriales	f__Flavobacteriaceae	g__Elizabethkingia	388	**0.00%**
d__Bacteria	k__unclassified_d__Bacteria	p__Bacteroidetes	c__Cytophagia	o__Cytophagales	f__Cytophagaceae	g__Fibrisoma	386	**0.00%**
d__Bacteria	k__unclassified_d__Bacteria	p__Fusobacteria	c__Fusobacteriia	o__Fusobacteriales	f__Fusobacteriaceae	g__Ilyobacter	384	**0.00%**
d__Bacteria	k__unclassified_d__Bacteria	p__Chloroflexi	c__unclassified_p__Chloroflexi	o__unclassified_p__Chloroflexi	f__unclassified_p__Chloroflexi	g__unclassified_p__Chloroflexi	378	**0.00%**
d__Bacteria	k__unclassified_d__Bacteria	p__Firmicutes	c__Negativicutes	o__Selenomonadales	f__Veillonellaceae	g__Megamonas	352	**0.00%**
d__Bacteria	k__unclassified_d__Bacteria	p__Firmicutes	c__Negativicutes	o__Selenomonadales	f__Veillonellaceae	g__Sporomusa	350	**0.00%**
d__Bacteria	k__unclassified_d__Bacteria	p__Actinobacteria	c__Actinobacteria	o__unclassified_c__Actinobacteria	f__unclassified_c__Actinobacteria	g__unclassified_c__Actinobacteria	346	**0.00%**
d__Bacteria	k__unclassified_d__Bacteria	p__Proteobacteria	c__Alphaproteobacteria	o__Rhizobiales	f__Brucellaceae	g__Ochrobactrum	344	**0.00%**
d__Bacteria	k__unclassified_d__Bacteria	p__Firmicutes	c__Clostridia	o__Clostridiales	f__Peptostreptococcaceae	g__Intestinibacter	336	**0.00%**
d__Bacteria	k__unclassified_d__Bacteria	p__Firmicutes	c__Bacilli	o__Lactobacillales	f__Aerococcaceae	g__Facklamia	328	**0.00%**
d__Bacteria	k__unclassified_d__Bacteria	p__Proteobacteria	c__Epsilonproteobacteria	o__Campylobacterales	f__Campylobacteraceae	g__Campylobacter	322	**0.00%**
d__Bacteria	k__unclassified_d__Bacteria	p__Firmicutes	c__Clostridia	o__Halanaerobiales	f__Halanaerobiaceae	g__Halothermothrix	320	**0.00%**
d__Bacteria	k__unclassified_d__Bacteria	p__Firmicutes	c__Bacilli	o__Bacillales	f__Paenibacillaceae	g__unclassified_f__Paenibacillaceae	318	**0.00%**
d__Archaea	k__unclassified_d__Archaea	p__Euryarchaeota	c__Halobacteria	o__Haloferacales	f__Haloferacaceae	g__Halogeometricum	316	**0.00%**
d__Bacteria	k__unclassified_d__Bacteria	p__Actinobacteria	c__Actinobacteria	o__Frankiales	f__Frankiaceae	g__Frankia	310	**0.00%**
d__Bacteria	k__unclassified_d__Bacteria	p__Planctomycetes	c__unclassified_p__Planctomycetes	o__unclassified_p__Planctomycetes	f__unclassified_p__Planctomycetes	g__unclassified_p__Planctomycetes	310	**0.00%**
d__Bacteria	k__unclassified_d__Bacteria	p__Proteobacteria	c__Gammaproteobacteria	o__Alteromonadales	f__Pseudoalteromonadaceae	g__Pseudoalteromonas	306	**0.00%**
d__Bacteria	k__unclassified_d__Bacteria	p__Firmicutes	c__Negativicutes	o__Selenomonadales	f__Veillonellaceae	g__Negativicoccus	298	**0.00%**
d__Bacteria	k__unclassified_d__Bacteria	p__Firmicutes	c__Clostridia	o__Thermoanaerobacterales	f__Thermodesulfobiaceae	g__Coprothermobacter	288	**0.00%**
d__Bacteria	k__unclassified_d__Bacteria	p__Thermotogae	c__Thermotogae	o__Petrotogales	f__unclassified_o__Petrotogales	g__Marinitoga	280	**0.00%**
d__Bacteria	k__unclassified_d__Bacteria	p__Proteobacteria	c__Alphaproteobacteria	o__Rhodospirillales	f__Acetobacteraceae	g__Roseomonas	274	**0.00%**
d__Bacteria	k__unclassified_d__Bacteria	p__Firmicutes	c__Clostridia	o__Clostridiales	f__Lachnospiraceae	g__Stomatobaculum	272	**0.00%**
d__Bacteria	k__unclassified_d__Bacteria	p__Actinobacteria	c__Actinobacteria	o__Streptomycetales	f__Streptomycetaceae	g__Streptomyces	264	**0.00%**
d__Bacteria	k__unclassified_d__Bacteria	p__Actinobacteria	c__Actinobacteria	o__Corynebacteriales	f__Mycobacteriaceae	g__Mycobacterium	260	**0.00%**
d__Bacteria	k__unclassified_d__Bacteria	p__Armatimonadetes	c__unclassified_p__Armatimonadetes	o__unclassified_p__Armatimonadetes	f__unclassified_p__Armatimonadetes	g__unclassified_p__Armatimonadetes	250	**0.00%**
d__Bacteria	k__unclassified_d__Bacteria	p__Proteobacteria	c__Gammaproteobacteria	o__Thiotrichales	f__Thiotrichaceae	g__Thioploca	242	**0.00%**
d__Bacteria	k__unclassified_d__Bacteria	p__Firmicutes	c__Negativicutes	o__Selenomonadales	f__Veillonellaceae	g__Propionispira	240	**0.00%**
d__Bacteria	k__unclassified_d__Bacteria	p__Firmicutes	c__Erysipelotrichia	o__Erysipelotrichales	f__Erysipelotrichaceae	g__Solobacterium	230	**0.00%**
d__Bacteria	k__unclassified_d__Bacteria	p__Actinobacteria	c__Actinobacteria	o__Pseudonocardiales	f__Pseudonocardiaceae	g__Lechevalieria	228	**0.00%**
d__Bacteria	k__unclassified_d__Bacteria	p__Cyanobacteria	c__unclassified_p__Cyanobacteria	o__Oscillatoriales	f__unclassified_o__Oscillatoriales	g__unclassified_o__Oscillatoriales	222	**0.00%**
d__Bacteria	k__unclassified_d__Bacteria	p__Synergistetes	c__Synergistia	o__Synergistales	f__Synergistaceae	g__Aminobacterium	220	**0.00%**
d__Bacteria	k__unclassified_d__Bacteria	p__Candidatus_Acetothermia	c__unclassified_p__Candidatus_Acetothermia	o__unclassified_p__Candidatus_Acetothermia	f__unclassified_p__Candidatus_Acetothermia	g__unclassified_p__Candidatus_Acetothermia	218	**0.00%**
d__Bacteria	k__unclassified_d__Bacteria	p__Chloroflexi	c__Anaerolineae	o__unclassified_c__Anaerolineae	f__unclassified_c__Anaerolineae	g__unclassified_c__Anaerolineae	206	**0.00%**
d__Bacteria	k__unclassified_d__Bacteria	p__Proteobacteria	c__Alphaproteobacteria	o__Rickettsiales	f__Holosporaceae	g__unclassified_f__Holosporaceae	202	**0.00%**
d__Bacteria	k__unclassified_d__Bacteria	p__Proteobacteria	c__Betaproteobacteria	o__Rhodocyclales	f__Rhodocyclaceae	g__Thauera	182	**0.00%**
d__Bacteria	k__unclassified_d__Bacteria	p__Proteobacteria	c__Betaproteobacteria	o__Burkholderiales	f__Burkholderiaceae	g__Ralstonia	180	**0.00%**
d__Bacteria	k__unclassified_d__Bacteria	p__Actinobacteria	c__Actinobacteria	o__Micromonosporales	f__Micromonosporaceae	g__Micromonospora	180	**0.00%**
d__Bacteria	k__unclassified_d__Bacteria	p__Firmicutes	c__Erysipelotrichia	o__Erysipelotrichales	f__Erysipelotrichaceae	g__Erysipelatoclostridium	178	**0.00%**
d__Bacteria	k__unclassified_d__Bacteria	p__Firmicutes	c__Negativicutes	o__Selenomonadales	f__Veillonellaceae	g__Mitsuokella	172	**0.00%**
d__Bacteria	k__unclassified_d__Bacteria	p__Candidatus_Levybacteria	c__unclassified_p__Candidatus_Levybacteria	o__unclassified_p__Candidatus_Levybacteria	f__unclassified_p__Candidatus_Levybacteria	g__unclassified_p__Candidatus_Levybacteria	172	**0.00%**
d__Archaea	k__unclassified_d__Archaea	p__Thaumarchaeota	c__unclassified_p__Thaumarchaeota	o__unclassified_p__Thaumarchaeota	f__unclassified_p__Thaumarchaeota	g__unclassified_p__Thaumarchaeota	172	**0.00%**
d__Bacteria	k__unclassified_d__Bacteria	p__Proteobacteria	c__Alphaproteobacteria	o__Sphingomonadales	f__Sphingomonadaceae	g__Sphingobium	162	**0.00%**
d__Bacteria	k__unclassified_d__Bacteria	p__Synergistetes	c__Synergistia	o__Synergistales	f__Synergistaceae	g__Synergistes	160	**0.00%**
d__Bacteria	k__unclassified_d__Bacteria	p__Cyanobacteria	c__unclassified_p__Cyanobacteria	o__Nostocales	f__Nostocaceae	g__Anabaena	154	**0.00%**
d__Bacteria	k__unclassified_d__Bacteria	p__Bacteroidetes	c__Flavobacteriia	o__Flavobacteriales	f__Flavobacteriaceae	g__Gramella	146	**0.00%**
d__Bacteria	k__unclassified_d__Bacteria	p__Actinobacteria	c__Actinobacteria	o__Micrococcales	f__Cellulomonadaceae	g__Cellulomonas	142	**0.00%**
d__Bacteria	k__unclassified_d__Bacteria	p__Deferribacteres	c__Deferribacteres	o__Deferribacterales	f__Deferribacteraceae	g__Calditerrivibrio	140	**0.00%**
d__Bacteria	k__unclassified_d__Bacteria	p__Proteobacteria	c__Deltaproteobacteria	o__Syntrophobacterales	f__Syntrophorhabdaceae	g__Syntrophorhabdus	138	**0.00%**
d__Bacteria	k__unclassified_d__Bacteria	p__Proteobacteria	c__Gammaproteobacteria	o__Enterobacteriales	f__Enterobacteriaceae	g__Erwinia	136	**0.00%**
d__Bacteria	k__unclassified_d__Bacteria	p__Actinobacteria	c__Coriobacteriia	o__Coriobacteriales	f__Coriobacteriaceae	g__Enorma	134	**0.00%**
d__Bacteria	k__unclassified_d__Bacteria	p__Proteobacteria	c__Betaproteobacteria	o__Burkholderiales	f__Burkholderiaceae	g__Cupriavidus	128	**0.00%**
d__Bacteria	k__unclassified_d__Bacteria	p__Proteobacteria	c__Gammaproteobacteria	o__Oceanospirillales	f__Halomonadaceae	g__Cobetia	122	**0.00%**
d__Bacteria	k__unclassified_d__Bacteria	p__Firmicutes	c__Negativicutes	o__Selenomonadales	f__Veillonellaceae	g__Megasphaera	118	**0.00%**
d__Bacteria	k__unclassified_d__Bacteria	p__Proteobacteria	c__Alphaproteobacteria	o__Rhodospirillales	f__Acetobacteraceae	g__Acetobacter	116	**0.00%**
d__Bacteria	k__unclassified_d__Bacteria	p__Proteobacteria	c__Deltaproteobacteria	o__Syntrophobacterales	f__Syntrophaceae	g__Desulfomonile	106	**0.00%**
d__Bacteria	k__unclassified_d__Bacteria	p__Proteobacteria	c__Deltaproteobacteria	o__Syntrophobacterales	f__Syntrophaceae	g__Syntrophus	106	**0.00%**
d__Bacteria	k__unclassified_d__Bacteria	p__Proteobacteria	c__Gammaproteobacteria	o__Oceanospirillales	f__Oleiphilaceae	g__Oleiphilus	100	**0.00%**
d__Bacteria	k__unclassified_d__Bacteria	p__Chloroflexi	c__Thermomicrobia	o__Sphaerobacterales	f__Sphaerobacteraceae	g__Sphaerobacter	98	**0.00%**
d__Bacteria	k__unclassified_d__Bacteria	p__Proteobacteria	c__Alphaproteobacteria	o__Rhizobiales	f__Methylobacteriaceae	g__Microvirga	96	**0.00%**
d__Bacteria	k__unclassified_d__Bacteria	p__Planctomycetes	c__Planctomycetia	o__Candidatus_Brocadiales	f__Candidatus_Brocadiaceae	g__Candidatus_Scalindua	94	**0.00%**
d__Bacteria	k__unclassified_d__Bacteria	p__Planctomycetes	c__Phycisphaerae	o__unclassified_c__Phycisphaerae	f__unclassified_c__Phycisphaerae	g__unclassified_c__Phycisphaerae	92	**0.00%**

d__Bacteria	k__unclassified_d__Bacteria	p__Bacteroidetes	c__unclassified_p__Bacteroidetes	o__Bacteroidetes_Order_II._Incertae_sedis	f__Rhodothermaceae	g__unclassified_f__Rhodothermaceae	90	0.00%
d__Bacteria	k__unclassified_d__Bacteria	p__Firmicutes	c__Limnochordia	o__Limnochordales	f__Limnochordaceae	g__Limnochorda	90	0.00%
d__Bacteria	k__unclassified_d__Bacteria	p__Firmicutes	c__Bacilli	o__Bacillales	f__Sporolactobacillaceae	g__unclassified_f__Sporolactobacillaceae	88	0.00%
d__Bacteria	k__unclassified_d__Bacteria	p__Firmicutes	c__Bacilli	o__Bacillales	f__Bacillaceae	g__Paraliobacillus	88	0.00%
d__Bacteria	k__unclassified_d__Bacteria	p__Proteobacteria	c__Betaproteobacteria	o__Burkholderiales	f__Comamonadaceae	g__Hydrogenophaga	88	0.00%
d__Bacteria	k__unclassified_d__Bacteria	p__Actinobacteria	c__Actinobacteria	o__Actinomycetales	f__Actinomycetaceae	g__Actinomyces	88	0.00%
d__Bacteria	k__unclassified_d__Bacteria	p__Firmicutes	c__Erysipelotrichia	o__Erysipelotrichales	f__Erysipelotrichaceae	g__Faecalibaculum	84	0.00%
d__Bacteria	k__unclassified_d__Bacteria	p__Firmicutes	c__Bacilli	o__Bacillales	f__Planococcaceae	g__Kurthia	84	0.00%
d__Bacteria	k__unclassified_d__Bacteria	p__Firmicutes	c__Erysipelotrichia	o__Erysipelotrichales	f__Erysipelotrichaceae	g__Holdemania	82	0.00%
d__Bacteria	k__unclassified_d__Bacteria	p__Proteobacteria	c__Alphaproteobacteria	o__Rhodospirillales	f__Rhodospirillaceae	g__Inquilinus	78	0.00%
d__Bacteria	k__unclassified_d__Bacteria	p__Deinococcus-Thermus	c__Deinococci	o__Thermales	f__Thermaceae	g__Thermus	76	0.00%
d__Bacteria	k__unclassified_d__Bacteria	p__Bacteroidetes	c__Flavobacteriia	o__Flavobacteriales	f__Flavobacteriaceae	g__Polaribacter	74	0.00%
d__Bacteria	k__unclassified_d__Bacteria	p__Actinobacteria	c__Actinobacteria	o__Streptosporangiales	f__Nocardiopsaceae	g__Nocardiopsis	74	0.00%
d__Bacteria	k__unclassified_d__Bacteria	p__Chloroflexi	c__Anaerolineae	o__Anaerolineales	f__Anaerolineaceae	g__Bellilinea	70	0.00%
d__Bacteria	k__unclassified_d__Bacteria	p__Spirochaetes	c__Spirochaetia	o__unclassified_c__Spirochaetia	f__Leptospiraceae	g__Leptospira	70	0.00%
d__Bacteria	k__unclassified_d__Bacteria	p__Proteobacteria	c__Alphaproteobacteria	o__Rhodospirillales	f__Rhodospirillaceae	g__Azospirillum	70	0.00%
d__Bacteria	k__unclassified_d__Bacteria	p__Verrucomicrobia	c__unclassified_p__Verrucomicrobia	o__unclassified_p__Verrucomicrobia	f__unclassified_p__Verrucomicrobia	g__unclassified_p__Verrucomicrobia	68	0.00%
d__Bacteria	k__unclassified_d__Bacteria	p__Aquificae	c__Aquificae	o__Desulfurobacteriales	f__Desulfurobacteriaceae	g__Desulfurobacterium	68	0.00%
d__Archaea	k__unclassified_d__Archaea	p__Euryarchaeota	c__unclassified_p__Euryarchaeota	o__unclassified_p__Euryarchaeota	f__unclassified_p__Euryarchaeota	g__Aciduliprofundum	68	0.00%
d__Bacteria	k__unclassified_d__Bacteria	p__Firmicutes	c__Bacilli	o__Bacillales	f__Bacillaceae	g__Amphibacillus	64	0.00%
d__Bacteria	k__unclassified_d__Bacteria	p__Cloacimonetes	c__unclassified_p__Cloacimonetes	o__unclassified_p__Cloacimonetes	f__unclassified_p__Cloacimonetes	g__Candidatus_Cloacimonas	64	0.00%
d__Bacteria	k__unclassified_d__Bacteria	p__Proteobacteria	c__Alphaproteobacteria	o__Rhizobiales	f__Bradyrhizobiaceae	g__Afipia	64	0.00%
d__Bacteria	k__unclassified_d__Bacteria	p__Thermotogae	c__Thermotogae	o__Mesoaciditogales	f__Mesoaciditogaceae	g__Mesoaciditoga	64	0.00%
d__Bacteria	k__unclassified_d__Bacteria	p__Bacteroidetes	c__unclassified_p__Bacteroidetes	o__Bacteroidetes_Order_II._Incertae_sedis	f__Rhodothermaceae	g__Rhodothermus	60	0.00%
d__Bacteria	k__unclassified_d__Bacteria	p__Proteobacteria	c__Alphaproteobacteria	o__Sphingomonadales	f__Sphingomonadaceae	g__Sphingomonas	60	0.00%
d__Bacteria	k__unclassified_d__Bacteria	p__Actinobacteria	c__Rubrobacteria	o__Rubrobacterales	f__Rubrobacteraceae	g__Rubrobacter	60	0.00%
d__Bacteria	k__unclassified_d__Bacteria	p__Firmicutes	c__Clostridia	o__Clostridiales	f__Lachnospiraceae	g__Catonella	60	0.00%
d__Bacteria	k__unclassified_d__Bacteria	p__Cyanobacteria	c__unclassified_p__Cyanobacteria	o__Nostocales	f__Microchaetaceae	g__Microchaete	58	0.00%
d__Bacteria	k__unclassified_d__Bacteria	p__Proteobacteria	c__Gammaproteobacteria	o__Xanthomonadales	f__Xanthomonadaceae	g__Pseudoxanthomonas	56	0.00%
d__Bacteria	k__unclassified_d__Bacteria	p__Candidatus_Rokubacteria	c__unclassified_p__Candidatus_Rokubacteria	o__unclassified_p__Candidatus_Rokubacteria	f__unclassified_p__Candidatus_Rokubacteria	g__unclassified_p__Candidatus_Rokubacteria	56	0.00%
d__Bacteria	k__unclassified_d__Bacteria	p__Bacteroidetes	c__Bacteroidia	o__Bacteroidales	f__Prevotellaceae	g__Prevotella	54	0.00%
d__Bacteria	k__unclassified_d__Bacteria	p__Proteobacteria	c__Alphaproteobacteria	o__Rhodospirillales	f__unclassified_o__Rhodospirillales	g__Elioraea	54	0.00%
d__Archaea	k__unclassified_d__Archaea	p__Euryarchaeota	c__Archaeoglobi	o__Archaeoglobales	f__Archaeoglobaceae	g__Archaeoglobus	54	0.00%
d__Bacteria	k__unclassified_d__Bacteria	p__Actinobacteria	c__Actinobacteria	o__Propionibacteriales	f__Nocardioidaceae	g__Aeromicrobium	52	0.00%
d__Bacteria	k__unclassified_d__Bacteria	p__Proteobacteria	c__Deltaproteobacteria	o__Desulfobacterales	f__Desulfobacteraceae	g__Desulfatirhabdium	50	0.00%
d__Bacteria	k__unclassified_d__Bacteria	p__Proteobacteria	c__Gammaproteobacteria	o__Pasteurellales	f__Pasteurellaceae	g__Mannheimia	50	0.00%
d__Bacteria	k__unclassified_d__Bacteria	p__Proteobacteria	c__Betaproteobacteria	o__Burkholderiales	f__Burkholderiaceae	g__Paraburkholderia	48	0.00%
d__Bacteria	k__unclassified_d__Bacteria	p__Cyanobacteria	c__unclassified_p__Cyanobacteria	o__Stigonematales	f__unclassified_o__Stigonematales	g__Fischerella	48	0.00%
d__Bacteria	k__unclassified_d__Bacteria	p__Actinobacteria	c__Actinobacteria	o__Streptosporangiales	f__Streptosporangiaceae	g__Planomonospora	48	0.00%
d__Bacteria	k__unclassified_d__Bacteria	p__Proteobacteria	c__Deltaproteobacteria	o__Syntrophobacterales	f__Syntrophaceae	g__Desulfobacca	48	0.00%
d__Bacteria	k__unclassified_d__Bacteria	p__Proteobacteria	c__Alphaproteobacteria	o__Caulobacterales	f__Caulobacteraceae	g__Asticcacaulis	48	0.00%
d__Bacteria	k__unclassified_d__Bacteria	p__Firmicutes	c__Bacilli	o__Bacillales	f__Bacillaceae	g__Psychrobacillus	46	0.00%
d__Bacteria	k__unclassified_d__Bacteria	p__Proteobacteria	c__Gammaproteobacteria	o__Enterobacteriales	f__Enterobacteriaceae	g__Buttiauxella	46	0.00%
d__Bacteria	k__unclassified_d__Bacteria	p__Bacteroidetes	c__Cytophagia	o__Cytophagales	f__Cyclobacteriaceae	g__Belliella	46	0.00%
d__Bacteria	k__unclassified_d__Bacteria	p__Thermotogae	c__Thermotogae	o__Kosmotogales	f__Kosmotogaceae	g__Kosmotoga	44	0.00%
d__Bacteria	k__unclassified_d__Bacteria	p__Bacteroidetes	c__Cytophagia	o__Cytophagales	f__Flammeovirgaceae	g__Flexithrix	44	0.00%
d__Bacteria	k__unclassified_d__Bacteria	p__Proteobacteria	c__Gammaproteobacteria	o__Orbales	f__Orbaceae	g__Frischella	42	0.00%
d__Bacteria	k__unclassified_d__Bacteria	p__Proteobacteria	c__Gammaproteobacteria	o__Cardiobacteriales	f__Cardiobacteriaceae	g__Cardiobacterium	40	0.00%
d__Bacteria	k__unclassified_d__Bacteria	p__Cyanobacteria	c__unclassified_p__Cyanobacteria	o__Chroococcales	f__unclassified_o__Chroococcales	g__Synechococcus	40	0.00%
d__Bacteria	k__unclassified_d__Bacteria	p__Actinobacteria	c__Coriobacteriia	o__Eggerthellales	f__Eggerthellaceae	g__Eggerthella	36	0.00%
d__Bacteria	k__unclassified_d__Bacteria	p__Bacteroidetes	c__Flavobacteriia	o__Flavobacteriales	f__Flavobacteriaceae	g__Aquimarina	36	0.00%
d__Bacteria	k__unclassified_d__Bacteria	p__Actinobacteria	c__Actinobacteria	o__Micrococcales	f__Microbacteriaceae	g__Leifsonia	36	0.00%
d__Eukaryota	k__Viridiplantae	p__Streptophyta	c__Marchantiopsida	o__Marchantiales	f__Marchantiaceae	g__Marchantia	36	0.00%
d__Bacteria	k__unclassified_d__Bacteria	p__Proteobacteria	c__Gammaproteobacteria	o__Oceanospirillales	f__Halomonadaceae	g__Halomonas	30	0.00%
d__Bacteria	k__unclassified_d__Bacteria	p__Chloroflexi	c__unclassified_p__Chloroflexi	o__unclassified_p__Chloroflexi	f__unclassified_p__Chloroflexi	g__Kouleothrix	30	0.00%
d__Archaea	k__unclassified_d__Archaea	p__Euryarchaeota	c__Halobacteria	o__Natrialbales	f__Natrialbaceae	g__Halopiger	30	0.00%
d__Bacteria	k__unclassified_d__Bacteria	p__Planctomycetes	c__Planctomycetia	o__Planctomycetales	f__Planctomycetaceae	g__Rhodopirellula	30	0.00%
d__Bacteria	k__unclassified_d__Bacteria	p__Proteobacteria	c__Gammaproteobacteria	o__Chromatiales	f__Chromatiaceae	g__Thiohalocapsa	28	0.00%
d__Bacteria	k__unclassified_d__Bacteria	p__Firmicutes	c__Erysipelotrichia	o__Erysipelotrichales	f__Erysipelotrichaceae	g__Dielma	28	0.00%
d__Bacteria	k__unclassified_d__Bacteria	p__Proteobacteria	c__Deltaproteobacteria	o__Syntrophobacterales	f__Syntrophaceae	g__Smithella	28	0.00%
d__Bacteria	k__unclassified_d__Bacteria	p__Actinobacteria	c__Thermoleophilia	o__Solirubrobacterales	f__Conexibacteraceae	g__Conexibacter	28	0.00%
d__Archaea	k__unclassified_d__Archaea	p__Euryarchaeota	c__Thermococci	o__Thermococcales	f__Thermococcaceae	g__Thermococcus	26	0.00%
d__Bacteria	k__unclassified_d__Bacteria	p__Verrucomicrobia	c__Opitutae	o__Opitutales	f__Opitutaceae	g__unclassified_f__Opitutaceae	26	0.00%
d__Bacteria	k__unclassified_d__Bacteria	p__Proteobacteria	c__Alphaproteobacteria	o__Caulobacterales	f__Caulobacteraceae	g__Caulobacter	26	0.00%
d__Bacteria	k__unclassified_d__Bacteria	p__Cyanobacteria	c__unclassified_p__Cyanobacteria	o__Nostocales	f__Microchaetaceae	g__Tolypothrix	24	0.00%
d__Archaea	k__unclassified_d__Archaea	p__Euryarchaeota	c__Thermoplasmata	o__Methanomassiliicoccales	f__unclassified_o__Methanomassiliicoccales	g__unclassified_o__Methanomassiliicoccales	24	0.00%
d__Bacteria	k__unclassified_d__Bacteria	p__Acidobacteria	c__Acidobacteriia	o__Acidobacteriales	f__Acidobacteriaceae	g__Edaphobacter	24	0.00%
d__Bacteria	k__unclassified_d__Bacteria	p__Bacteroidetes	c__Flavobacteriia	o__Flavobacteriales	f__Flavobacteriaceae	g__Dokdonia	24	0.00%
d__Bacteria	k__unclassified_d__Bacteria	p__Proteobacteria	c__Deltaproteobacteria	o__Desulfuromonadales	f__Desulfuromonadaceae	g__Desulfuromonas	22	0.00%
d__Bacteria	k__unclassified_d__Bacteria	p__Proteobacteria	c__Gammaproteobacteria	o__Methylococcales	f__Methylococcaceae	g__Methylomicrobium	22	0.00%
d__Bacteria	k__unclassified_d__Bacteria	p__Proteobacteria	c__Gammaproteobacteria	o__Enterobacteriales	f__Enterobacteriaceae	g__Escherichia	22	0.00%
d__Bacteria	k__unclassified_d__Bacteria	p__Proteobacteria	c__Alphaproteobacteria	o__Rhizobiales	f__Bradyrhizobiaceae	g__Salinarimonas	22	0.00%
d__Bacteria	k__unclassified_d__Bacteria	p__Firmicutes	c__Bacilli	o__Bacillales	f__Planococcaceae	g__Planomicrobium	22	0.00%
d__Bacteria	k__unclassified_d__Bacteria	p__Candidatus_Magasanikbacteria	c__unclassified_p__Candidatus_Magasanikbacteria	o__unclassified_p__Candidatus_Magasanikbacteria	f__unclassified_p__Candidatus_Magasanikbacteria	g__unclassified_p__Candidatus_Magasanikbacteria	22	0.00%
d__Bacteria	k__unclassified_d__Bacteria	p__Proteobacteria	c__Gammaproteobacteria	o__Chromatiales	f__Ectothiorhodospiraceae	g__Alkalilimnicola	20	0.00%
d__Viruses	k__unclassified_d__Viruses	p__unclassified_d__Viruses	c__unclassified_d__Viruses	o__Caudovirales	f__Podoviridae	g__unclassified_f__Podoviridae	20	0.00%
d__Bacteria	k__unclassified_d__Bacteria	p__Spirochaetes	c__Spirochaetia	o__Spirochaetales	f__Borreliaceae	g__Borreliella	20	0.00%
d__Bacteria	k__unclassified_d__Bacteria	p__candidate_division_WWE3	c__unclassified_p__candidate_division_WWE3	o__unclassified_p__candidate_division_WWE3	f__unclassified_p__candidate_division_WWE3	g__unclassified_p__candidate_division_WWE3	18	0.00%
d__Bacteria	k__unclassified_d__Bacteria	p__Firmicutes	c__Bacilli	o__Bacillales	f__Bacillaceae	g__Anaerobacillus	18	0.00%
d__Bacteria	k__unclassified_d__Bacteria	p__Bacteroidetes	c__Sphingobacteriia	o__Sphingobacteriales	f__Chitinophagaceae	g__Filimonas	16	0.00%
d__Bacteria	k__unclassified_d__Bacteria	p__Firmicutes	c__Negativicutes	o__Selenomonadales	f__Acidaminococcaceae	g__Phascolarctobacterium	16	0.00%
d__Bacteria	k__unclassified_d__Bacteria	p__Verrucomicrobia	c__Verrucomicrobiae	o__Verrucomicrobiales	f__Verrucomicrobiaceae	g__unclassified_f__Verrucomicrobiaceae	16	0.00%
d__Bacteria	k__unclassified_d__Bacteria	p__Spirochaetes	c__unclassified_p__Spirochaetes	o__unclassified_p__Spirochaetes	f__unclassified_p__Spirochaetes	g__unclassified_p__Spirochaetes	16	0.00%
d__Bacteria	k__unclassified_d__Bacteria	p__Bacteroidetes	c__Flavobacteriia	o__Flavobacteriales	f__Flavobacteriaceae	g__Chryseobacterium	16	0.00%
d__Bacteria	k__unclassified_d__Bacteria	p__Cyanobacteria	c__unclassified_p__Cyanobacteria	o__Nostocales	f__Nostocaceae	g__Cylindrospermum	16	0.00%
d__Bacteria	k__unclassified_d__Bacteria	p__Actinobacteria	c__Actinobacteria	o__Propionibacteriales	f__Nocardioidaceae	g__Nocardioides	16	0.00%
d__Bacteria	k__unclassified_d__Bacteria	p__Actinobacteria	c__Actinobacteria	o__Micrococcales	f__Microbacteriaceae	g__Microbacterium	16	0.00%
d__Bacteria	k__unclassified_d__Bacteria	p__Actinobacteria	c__Actinobacteria	o__Acidothermales	f__Acidothermaceae	g__Acidothermus	14	0.00%
d__Bacteria	k__unclassified_d__Bacteria	p__candidate_division_Zixibacteria	c__unclassified_p__candidate_division_Zixibacteria	o__unclassified_p__candidate_division_Zixibacteria	f__unclassified_p__candidate_division_Zixibacteria	g__unclassified_p__candidate_division_Zixibacteria	14	0.00%
d__Bacteria	k__unclassified_d__Bacteria	p__Proteobacteria	c__Betaproteobacteria	o__Rhodocyclales	f__Rhodocyclaceae	g__Azoarcus	14	0.00%
d__Bacteria	k__unclassified_d__Bacteria	p__Candidatus_Moranbacteria	c__unclassified_p__Candidatus_Moranbacteria	o__unclassified_p__Candidatus_Moranbacteria	f__unclassified_p__Candidatus_Moranbacteria	g__unclassified_p__Candidatus_Moranbacteria	12	0.00%
d__Bacteria	k__unclassified_d__Bacteria	p__Proteobacteria	c__Deltaproteobacteria	o__Myxococcales	f__Polyangiaceae	g__Sorangium	12	0.00%
d__Bacteria	k__unclassified_d__Bacteria	p__Proteobacteria	c__Gammaproteobacteria	o__Xanthomonadales	f__Xanthomonadaceae	g__Arenimonas	12	0.00%
d__Bacteria	k__unclassified_d__Bacteria	p__Proteobacteria	c__Alphaproteobacteria	o__Rhizobiales	f__Rhizobiaceae	g__Kaistia	12	0.00%
d__Bacteria	k__unclassified_d__Bacteria	p__Firmicutes	c__Erysipelotrichia	o__Erysipelotrichales	f__Erysipelotrichaceae	g__Faecalitalea	12	0.00%
d__Bacteria	k__unclassified_d__Bacteria	p__Firmicutes	c__Clostridia	o__Clostridiales	f__Lachnospiraceae	g__Shuttleworthia	12	0.00%
d__Bacteria	k__unclassified_d__Bacteria	p__Latescibacteria	c__unclassified_p__Latescibacteria	o__unclassified_p__Latescibacteria	f__unclassified_p__Latescibacteria	g__unclassified_p__Latescibacteria	12	0.00%
d__Bacteria	k__unclassified_d__Bacteria	p__Cyanobacteria	c__unclassified_p__Cyanobacteria	o__Oscillatoriales	f__unclassified_o__Oscillatoriales	g__Microcoleus	10	0.00%
d__Bacteria	k__unclassified_d__Bacteria	p__Proteobacteria	c__Gammaproteobacteria	o__Pasteurellales	f__Pasteurellaceae	g__Pasteurella	10	0.00%
d__Bacteria	k__unclassified_d__Bacteria	p__Actinobacteria	c__Actinobacteria	o__Micrococcales	f__Intrasporangiaceae	g__Knoellia	10	0.00%
d__Bacteria	k__unclassified_d__Bacteria	p__Candidatus_Shapirobacteria	c__unclassified_p__Candidatus_Shapirobacteria	o__unclassified_p__Candidatus_Shapirobacteria	f__unclassified_p__Candidatus_Shapirobacteria	g__unclassified_p__Candidatus_Shapirobacteria	10	0.00%
d__Bacteria	k__unclassified_d__Bacteria	p__Verrucomicrobia	c__Spartobacteria	o__unclassified_c__Spartobacteria	f__unclassified_c__Spartobacteria	g__Terrimicrobium	10	0.00%
d__Bacteria	k__unclassified_d__Bacteria	p__Proteobacteria	c__Betaproteobacteria	o__Burkholderiales	f__Comamonadaceae	g__Ramlibacter	10	0.00%
d__Bacteria	k__unclassified_d__Bacteria	p__Proteobacteria	c__Deltaproteobacteria	o__Desulfobacterales	f__unclassified_o__Desulfobacterales	g__unclassified_o__Desulfobacterales	8	0.00%
d__Bacteria	k__unclassified_d__Bacteria	p__Bacteroidetes	c__Sphingobacteriia	o__Sphingobacteriales	f__Chitinophagaceae	g__Chitinophaga	8	0.00%
d__Bacteria	k__unclassified_d__Bacteria	p__Proteobacteria	c__Gammaproteobacteria	o__Thiotrichales	f__Francisellaceae	g__Francisella	8	0.00%
d__Bacteria	k__unclassified_d__Bacteria	p__Candidatus_Roizmanbacteria	c__unclassified_p__Candidatus_Roizmanbacteria	o__unclassified_p__Candidatus_Roizmanbacteria	f__unclassified_p__Candidatus_Roizmanbacteria	g__unclassified_p__Candidatus_Roizmanbacteria	8	0.00%
d__Bacteria	k__unclassified_d__Bacteria	p__Bacteroidetes	c__Cytophagia	o__Cytophagales	f__Cytophagaceae	g__Dyadobacter	8	0.00%
d__Bacteria	k__unclassified_d__Bacteria	p__Proteobacteria	c__Alphaproteobacteria	o__Rickettsiales	f__Rickettsiaceae	g__Rickettsia	8	0.00%
d__Bacteria	k__unclassified_d__Bacteria	p__Proteobacteria	c__Gammaproteobacteria	o__Enterobacteriales	f__Enterobacteriaceae	g__Enterobacter	8	0.00%
d__Archaea	k__unclassified_d__Archaea	p__Euryarchaeota	c__Methanococci	o__Methanococcales	f__Methanococcaceae	g__Methanothermococcus	8	0.00%
d__Bacteria	k__unclassified_d__Bacteria	p__Proteobacteria	c__Deltaproteobacteria	o__Myxococcales	f__Cystobacteraceae	g__Cystobacter	8	0.00%
d__Archaea	k__unclassified_d__Archaea	p__Euryarchaeota	c__Thermoplasmata	o__Thermoplasmatales	f__unclassified_o__Thermoplasmatales	g__unclassified_o__Thermoplasmatales	8	0.00%
d__Bacteria	k__unclassified_d__Bacteria	p__Bacteroidetes	c__Bacteroidia	o__Bacteroidales	f__Marinilabiliaceae	g__Anaerophaga	8	0.00%
d__Eukaryota	k__Metazoa	p__Nematoda	c__Enoplea	o__Trichocephalida	f__Trichinellidae	g__Trichinella	8	0.00%
d__Bacteria	k__unclassified_d__Bacteria	p__Synergistetes	c__Synergistia	o__Synergistales	f__unclassified_o__Synergistales	g__unclassified_o__Synergistales	8	0.00%
d__Bacteria	k__unclassified_d__Bacteria	p__Firmicutes	c__Negativicutes	o__Selenomonadales	f__Veillonellaceae	g__Centipeda	8	0.00%
d__Bacteria	k__unclassified_d__Bacteria	p__Tenericutes	c__Mollicutes	o__Entomoplasmatales	f__Spiroplasmataceae	g__Spiroplasma	6	0.00%
d__Bacteria	k__unclassified_d__Bacteria	p__Proteobacteria	c__Alphaproteobacteria	o__Rhodobacterales	f__Rhodobacteraceae	g__Loktanella	6	0.00%
d__Eukaryota	k__unclassified_d__Eukaryota	p__Apicomplexa	c__Aconoidasida	o__Haemosporida	f__Plasmodiidae	g__Plasmodium	6	0.00%
d__Bacteria	k__unclassified_d__Bacteria	p__Synergistetes	c__Synergistia	o__Synergistales	f__Synergistaceae	g__Thermanaerovibrio	6	0.00%
d__Bacteria	k__unclassified_d__Bacteria	p__Proteobacteria	c__Betaproteobacteria	o__Burkholderiales	f__unclassified_o__Burkholderiales	g__Thiomonas	6	0.00%
d__Bacteria	k__unclassified_d__Bacteria	p__Thermodesulfobacteria	c__Thermodesulfobacteria	o__Thermodesulfobacteriales	f__Thermodesulfobacteriaceae	g__Thermodesulfatator	6	0.00%
d__Bacteria	k__unclassified_d__Bacteria	p__Actinobacteria	c__Actinobacteria	o__Propionibacteriales	f__Nocardioidaceae	g__Propionicicella	6	0.00%
d__Bacteria	k__unclassified_d__Bacteria	p__Firmicutes	c__Clostridia	o__Clostridiales	f__Peptococcaceae	g__Candidatus_Desulforudis	6	0.00%
d__Eukaryota	k__Metazoa	p__Chordata	c__Mammalia	o__Rodentia	f__Muridae	g__Mus	4	0.00%
d__Bacteria	k__unclassified_d__Bacteria	p__Proteobacteria	c__Deltaproteobacteria	o__Desulfovibrionales	f__Desulfonatronaceae	g__Desulfonatronum	4	0.00%
d__Bacteria	k__unclassified_d__Bacteria	p__Actinobacteria	c__Actinobacteria	o__Actinomycetales	f__Actinomycetaceae	g__Mobiluncus	4	0.00%
d__Bacteria	k__unclassified_d__Bacteria	p__Bacteroidetes	c__Flavobacteriia	o__Flavobacteriales	f__Flavobacteriaceae	g__Weeksella	4	0.00%
d__Bacteria	k__unclassified_d__Bacteria	p__Actinobacteria	c__Actinobacteria	o__Streptosporangiales	f__Streptosporangiaceae	g__Microbispora	4	0.00%
d__Bacteria	k__unclassified_d__Bacteria	p__Chloroflexi	c__Thermomicrobia	o__Thermomicrobiales	f__Thermomicrobiaceae	g__Thermomicrobium	4	0.00%
d__Bacteria	k__unclassified_d__Bacteria	p__Proteobacteria	c__Gammaproteobacteria	o__Enterobacteriales	f__Enterobacteriaceae	g__Shigella	4	0.00%
d__Archaea	k__unclassified_d__Archaea	p__Euryarchaeota	c__Halobacteria	o__Natrialbales	f__Natrialbaceae	g__Natrinema	4	0.00%
d__Bacteria	k__unclassified_d__Bacteria	p__Proteobacteria	c__Deltaproteobacteria	o__Syntrophobacterales	f__Syntrophobacteraceae	g__Syntrophobacter	4	0.00%
d__Bacteria	k__unclassified_d__Bacteria	p__Proteobacteria	c__Gammaproteobacteria	o__Legionellales	f__Legionellaceae	g__Legionella	4	0.00%
d__Bacteria	k__unclassified_d__Bacteria	p__Verrucomicrobia	c__Opitutae	o__Opitutales	f__Opitutaceae	g__Diplosphaera	4	0.00%
d__Bacteria	k__unclassified_d__Bacteria	p__Bacteroidetes	c__Flavobacteriia	o__Flavobacteriales	f__Flavobacteriaceae	g__Zobellia	4	0.00%
d__Bacteria	k__unclassified_d__Bacteria	p__Proteobacteria	c__Gammaproteobacteria	o__Chromatiales	f__Chromatiaceae	g__Thiocystis	4	0.00%
d__Bacteria	k__unclassified_d__Bacteria	p__Actinobacteria	c__Actinobacteria	o__Corynebacteriales	f__Nocardiaceae	g__Nocardia	4	0.00%
d__Bacteria	k__unclassified_d__Bacteria	p__Proteobacteria	c__Gammaproteobacteria	o__Pasteurellales	f__Pasteurellaceae	g__Haemophilus	4	0.00%
d__Bacteria	k__unclassified_d__Bacteria	p__Bacteroidetes	c__Sphingobacteriia	o__Sphingobacteriales	f__Chitinophagaceae	g__Niabella	4	0.00%
d__Eukaryota	k__Fungi	p__Ascomycota	c__Leotiomycetes	o__unclassified_c__Leotiomycetes	f__Pseudeurotiaceae	g__Pseudogymnoascus	4	0.00%
d__Bacteria	k__unclassified_d__Bacteria	p__Bacteroidetes	c__Flavobacteriia	o__Flavobacteriales	f__Flavobacteriaceae	g__Lutibacter	4	0.00%
d__Bacteria	k__unclassified_d__Bacteria	p__Cyanobacteria	c__unclassified_p__Cyanobacteria	o__Pleurocapsales	f__unclassified_o__Pleurocapsales	g__Pleurocapsa	2	0.00%
d__Bacteria	k__unclassified_d__Bacteria	p__Actinobacteria	c__Actinobacteria	o__Pseudonocardiales	f__Pseudonocardiaceae	g__Amycolatopsis	2	0.00%
d__Bacteria	k__unclassified_d__Bacteria	p__Bacteroidetes	c__Cytophagia	o__Cytophagales	f__Flammeovirgaceae	g__unclassified_f__Flammeovirgaceae	2	0.00%
d__Bacteria	k__unclassified_d__Bacteria	p__Proteobacteria	c__Alphaproteobacteria	o__Rhizobiales	f__Aurantimonadaceae	g__Aureimonas	2	0.00%
d__Eukaryota	k__Viridiplantae	p__Streptophyta	c__Liliopsida	o__Poales	f__Poaceae	g__Setaria	2	0.00%
d__Bacteria	k__unclassified_d__Bacteria	p__Cyanobacteria	c__unclassified_p__Cyanobacteria	o__Nostocales	f__Nostocaceae	g__Chrysosporum	2	0.00%

d__Bacteria	k__unclassified_d__Bacteria	p__Actinobacteria	c__Actinobacteria	o__Streptomycetales	f__Streptomycetaceae	g__Kitasatospora	2	0.00%
d__Bacteria	k__unclassified_d__Bacteria	p__Firmicutes	c__Clostridia	o__Clostridiales	f__unclassified_o__Clostridiales	g__Epulopiscium	2	0.00%
d__Bacteria	k__unclassified_d__Bacteria	p__Proteobacteria	c__Gammaproteobacteria	o__Pseudomonadales	f__Moraxellaceae	g__Moraxella	2	0.00%
d__Bacteria	k__unclassified_d__Bacteria	p__Firmicutes	c__Clostridia	o__Clostridiales	f__Lachnospiraceae	g__Lachnospira	2	0.00%
d__Bacteria	k__unclassified_d__Bacteria	p__Bacteroidetes	c__Bacteroidia	o__Bacteroidales	f__Porphyromonadaceae	g__Coprobacter	2	0.00%
d__Bacteria	k__unclassified_d__Bacteria	p__Firmicutes	c__Clostridia	o__Clostridiales	f__unclassified_o__Clostridiales	g__Fenollaria	2	0.00%
d__Archaea	k__unclassified_d__Archaea	p__Euryarchaeota	c__Halobacteria	o__Natrialbales	f__Natrialbaceae	g__Natronococcus	2	0.00%
d__Bacteria	k__unclassified_d__Bacteria	p__Firmicutes	c__Negativicutes	o__Selenomonadales	f__Veillonellaceae	g__Anaeroarcus	2	0.00%
d__Bacteria	k__unclassified_d__Bacteria	p__Candidatus_Nomurabacteria	c__unclassified_p__Candidatus_Nomurabacteria	o__unclassified_p__Candidatus_Nomurabacteria	f__unclassified_p__Candidatus_Nomurabacteria	g__unclassified_p__Candidatus_Nomurabacteria	2	0.00%
d__Bacteria	k__unclassified_d__Bacteria	p__Cyanobacteria	c__unclassified_p__Cyanobacteria	o__Chroococcales	f__unclassified_o__Chroococcales	g__Cyanothece	2	0.00%
d__Eukaryota	k__Viridiplantae	p__Chlorophyta	c__Mamiellophyceae	o__Mamiellales	f__Mamiellaceae	g__Micromonas	2	0.00%
d__Bacteria	k__unclassified_d__Bacteria	p__Actinobacteria	c__Actinobacteria	o__Micromonosporales	f__Micromonosporaceae	g__Dactylosporangium	2	0.00%
d__Archaea	k__unclassified_d__Archaea	p__Euryarchaeota	c__Halobacteria	o__Haloferacales	f__Haloferacaceae	g__Haloplanus	2	0.00%
d__Bacteria	k__unclassified_d__Bacteria	p__Proteobacteria	c__Alphaproteobacteria	o__Rhizobiales	f__Methylocystaceae	g__Pleomorphomonas	2	0.00%
d__Bacteria	k__unclassified_d__Bacteria	p__Actinobacteria	c__Actinobacteria	o__Bifidobacteriales	f__Bifidobacteriaceae	g__Parascardovia	2	0.00%
d__Bacteria	k__unclassified_d__Bacteria	p__Firmicutes	c__Clostridia	o__Clostridiales	f__Lachnospiraceae	g__Lachnobacterium	2	0.00%
d__Bacteria	k__unclassified_d__Bacteria	p__Firmicutes	c__Negativicutes	o__Selenomonadales	f__Acidaminococcaceae	g__Succinispira	2	0.00%
d__Eukaryota	k__Viridiplantae	p__Streptophyta	c__unclassified_p__Streptophyta	o__Caryophyllales	f__Amaranthaceae	g__Beta	2	0.00%
d__Bacteria	k__unclassified_d__Bacteria	p__Planctomycetes	c__Planctomycetia	o__Candidatus_Brocadiales	f__Candidatus_Brocadiaceae	g__Candidatus_Jettenia	2	0.00%
d__Bacteria	k__unclassified_d__Bacteria	p__Proteobacteria	c__Gammaproteobacteria	o__Oceanospirillales	f__Halomonadaceae	g__unclassified_f__Halomonadaceae	2	0.00%
d__Bacteria	k__unclassified_d__Bacteria	p__Proteobacteria	c__Gammaproteobacteria	o__Thiotrichales	f__Thiotrichaceae	g__Beggiatoa	2	0.00%
d__Bacteria	k__unclassified_d__Bacteria	p__Planctomycetes	c__Planctomycetia	o__Planctomycetales	f__Planctomycetaceae	g__unclassified_f__Planctomycetaceae	2	0.00%
d__Bacteria	k__unclassified_d__Bacteria	p__Candidatus_Peregrinibacteria	c__unclassified_p__Candidatus_Peregrinibacteria	o__unclassified_p__Candidatus_Peregrinibacteria	f__unclassified_p__Candidatus_Peregrinibacteria	g__unclassified_p__Candidatus_Peregrinibacteria	2	0.00%
d__Bacteria	k__unclassified_d__Bacteria	p__Bacteroidetes	c__Flavobacteriia	o__Flavobacteriales	f__Flavobacteriaceae	g__Leeuwenhoekiella	2	0.00%
d__Eukaryota	k__Viridiplantae	p__Streptophyta	c__unclassified_p__Streptophyta	o__Rosales	f__Urticaceae	g__Boehmeria	0	0.00%
d__Bacteria	k__unclassified_d__Bacteria	p__Actinobacteria	c__Actinobacteria	o__Catenulisporales	f__Catenulisporaceae	g__Catenulispora	0	0.00%
d__Eukaryota	k__Viridiplantae	p__Streptophyta	c__unclassified_p__Streptophyta	o__Rosales	f__Cannabaceae	g__Humulus	0	0.00%
d__Viruses	k__unclassified_d__Viruses	p__unclassified_d__Viruses	c__unclassified_d__Viruses	o__Picornavirales	f__Secoviridae	g__Fabavirus	0	0.00%
d__Bacteria	k__unclassified_d__Bacteria	p__Ignavibacteriae	c__Ignavibacteria	o__Ignavibacteriales	f__Ignavibacteriaceae	g__Ignavibacterium	0	0.00%
d__Eukaryota	k__Viridiplantae	p__Streptophyta	c__unclassified_p__Streptophyta	o__Fabales	f__Fabaceae	g__Medicago	0	0.00%
d__Eukaryota	k__Viridiplantae	p__Streptophyta	c__unclassified_p__Streptophyta	o__Solanales	f__Solanaceae	g__Nicotiana	0	0.00%
d__Eukaryota	k__Viridiplantae	p__Streptophyta	c__unclassified_p__Streptophyta	o__Malpighiales	f__Salicaceae	g__Populus	0	0.00%
d__Eukaryota	k__Viridiplantae	p__Streptophyta	c__unclassified_p__Streptophyta	o__Lamiales	f__Pedaliaceae	g__Sesamum	0	0.00%
d__Eukaryota	k__Viridiplantae	p__Streptophyta	c__unclassified_p__Streptophyta	o__Malpighiales	f__Euphorbiaceae	g__Jatropha	0	0.00%
d__Eukaryota	k__Viridiplantae	p__Streptophyta	c__unclassified_p__Streptophyta	o__Malpighiales	f__Euphorbiaceae	g__Manihot	0	0.00%
d__Eukaryota	k__Viridiplantae	p__Streptophyta	c__unclassified_p__Streptophyta	o__Fabales	f__Fabaceae	g__Glycine	0	0.00%
d__Eukaryota	k__Viridiplantae	p__Streptophyta	c__unclassified_p__Streptophyta	o__Rosales	f__Rosaceae	g__Eriobotrya	0	0.00%
d__Eukaryota	k__Viridiplantae	p__Streptophyta	c__unclassified_p__Streptophyta	o__Vitales	f__Vitaceae	g__Vitis	0	0.00%
d__Eukaryota	k__Viridiplantae	p__Streptophyta	c__Liliopsida	o__Arecales	f__Arecaceae	g__Elaeis	0	0.00%
d__Eukaryota	k__Viridiplantae	p__Streptophyta	c__unclassified_p__Streptophyta	o__Rosales	f__Rhamnaceae	g__Ziziphus	0	0.00%
d__Bacteria	k__unclassified_d__Bacteria	p__Candidatus_Woesebacteria	c__unclassified_p__Candidatus_Woesebacteria	o__unclassified_p__Candidatus_Woesebacteria	f__unclassified_p__Candidatus_Woesebacteria	g__unclassified_p__Candidatus_Woesebacteria	0	0.00%
d__Bacteria	k__unclassified_d__Bacteria	p__Firmicutes	c__Clostridia	o__Clostridiales	f__unclassified_o__Clostridiales	g__Proteocatella	0	0.00%
d__Eukaryota	k__Viridiplantae	p__Streptophyta	c__unclassified_p__Streptophyta	o__Fabales	f__Fabaceae	g__Arachis	0	0.00%
d__Eukaryota	k__Metazoa	p__Chordata	c__Mammalia	o__unclassified_c__Mammalia	f__Tenrecidae	g__Echinops	0	0.00%
d__Archaea	k__unclassified_d__Archaea	p__Euryarchaeota	c__Thermococci	o__Thermococcales	f__unclassified_o__Thermococcales	g__unclassified_o__Thermococcales	0	0.00%
d__Bacteria	k__unclassified_d__Bacteria	p__Firmicutes	c__Tissierellia	o__Tissierellales	f__Peptoniphilaceae	g__Helcococcus	0	0.00%
d__Bacteria	k__unclassified_d__Bacteria	p__Firmicutes	c__Clostridia	o__Clostridiales	f__Lachnospiraceae	g__Johnsonella	0	0.00%
d__Bacteria	k__unclassified_d__Bacteria	p__Bacteroidetes	c__Cytophagia	o__Cytophagales	f__Flammeovirgaceae	g__Nafulsella	0	0.00%
d__Bacteria	k__unclassified_d__Bacteria	p__Proteobacteria	c__Betaproteobacteria	o__Burkholderiales	f__Oxalobacteraceae	g__Massilia	0	0.00%
d__Eukaryota	k__Viridiplantae	p__Streptophyta	c__unclassified_p__Streptophyta	o__Malvales	f__Malvaceae	g__Gossypium	0	0.00%
d__Eukaryota	k__Metazoa	p__Chordata	c__Mammalia	o__Rodentia	f__Dipodidae	g__Jaculus	0	0.00%
d__Eukaryota	k__Viridiplantae	p__Streptophyta	c__unclassified_p__Streptophyta	o__Fabales	f__Fabaceae	g__Vigna	0	0.00%
d__Bacteria	k__unclassified_d__Bacteria	p__candidate_division_NC10	c__unclassified_p__candidate_division_NC10	o__unclassified_p__candidate_division_NC10	f__unclassified_p__candidate_division_NC10	g__unclassified_p__candidate_division_NC10	0	0.00%
d__Bacteria	k__unclassified_d__Bacteria	p__Proteobacteria	c__Betaproteobacteria	o__Burkholderiales	f__Burkholderiaceae	g__unclassified_f__Burkholderiaceae	0	0.00%
d__Eukaryota	k__Viridiplantae	p__Streptophyta	c__unclassified_p__Streptophyta	o__Myrtales	f__Melastomataceae	g__Melastoma	0	0.00%
d__Eukaryota	k__Viridiplantae	p__Streptophyta	c__unclassified_p__Streptophyta	o__Sapindales	f__Rutaceae	g__Citrus	0	0.00%
d__Bacteria	k__unclassified_d__Bacteria	p__Firmicutes	c__Tissierellia	o__Tissierellales	f__Peptoniphilaceae	g__Finegoldia	0	0.00%
d__Eukaryota	k__Viridiplantae	p__Streptophyta	c__unclassified_p__Streptophyta	o__Fabales	f__Fabaceae	g__Phaseolus	0	0.00%
d__Eukaryota	k__Viridiplantae	p__Streptophyta	c__unclassified_p__Streptophyta	o__Fabales	f__Fabaceae	g__Lotus	0	0.00%
d__Eukaryota	k__Viridiplantae	p__Streptophyta	c__unclassified_p__Streptophyta	o__Brassicales	f__Brassicaceae	g__Arabidopsis	0	0.00%
d__Bacteria	k__unclassified_d__Bacteria	p__Firmicutes	c__Bacilli	o__Bacillales	f__Planococcaceae	g__Solibacillus	0	0.00%
d__Eukaryota	k__Viridiplantae	p__Streptophyta	c__unclassified_p__Streptophyta	o__Myrtales	f__Myrtaceae	g__Eucalyptus	0	0.00%
d__Bacteria	k__unclassified_d__Bacteria	p__Proteobacteria	c__Gammaproteobacteria	o__Cellvibrionales	f__Cellvibrionaceae	g__Marinimicrobium	0	0.00%
d__Eukaryota	k__Metazoa	p__Chordata	c__Actinopteri	o__Perciformes	f__Serranidae	g__Epinephelus	0	0.00%
d__Bacteria	k__unclassified_d__Bacteria	p__Proteobacteria	c__Gammaproteobacteria	o__Legionellales	f__Coxiellaceae	g__Coxiella	0	0.00%
d__Eukaryota	k__Viridiplantae	p__Streptophyta	c__unclassified_p__Streptophyta	o__Asterales	f__Asteraceae	g__Chrysanthemum	0	0.00%
d__Eukaryota	k__Viridiplantae	p__Streptophyta	c__unclassified_p__Streptophyta	o__Apiales	f__Apiaceae	g__Daucus	0	0.00%
d__Eukaryota	k__Fungi	p__Ascomycota	c__Saccharomycetes	o__Saccharomycetales	f__Phaffomycetaceae	g__Komagataella	0	0.00%
d__Eukaryota	k__Viridiplantae	p__Streptophyta	c__Liliopsida	o__Poales	f__Poaceae	g__Zea	0	0.00%
d__Bacteria	k__unclassified_d__Bacteria	p__Firmicutes	c__Clostridia	o__Clostridiales	f__Lachnospiraceae	g__Lachnoanaerobaculum	0	0.00%
d__Eukaryota	k__Metazoa	p__Chordata	c__Mammalia	o__Rodentia	f__Muridae	g__Rattus	0	0.00%
d__Bacteria	k__unclassified_d__Bacteria	p__Acidobacteria	c__unclassified_p__Acidobacteria	o__unclassified_p__Acidobacteria	f__unclassified_p__Acidobacteria	g__unclassified_p__Acidobacteria	0	0.00%
d__Bacteria	k__unclassified_d__Bacteria	p__Proteobacteria	c__Deltaproteobacteria	o__Desulfuromonadales	f__Pelobacteraceae	g__Pelobacter	0	0.00%
d__Bacteria	k__unclassified_d__Bacteria	p__Chloroflexi	c__Dehalococcoidia	o__unclassified_c__Dehalococcoidia	f__unclassified_c__Dehalococcoidia	g__unclassified_c__Dehalococcoidia	0	0.00%
d__Eukaryota	k__Viridiplantae	p__Streptophyta	c__unclassified_p__Streptophyta	o__Caryophyllales	f__Amaranthaceae	g__Spinacia	0	0.00%
d__Archaea	k__unclassified_d__Archaea	p__Crenarchaeota	c__Thermoprotei	o__Thermoproteales	f__Thermofilaceae	g__Thermofilum	0	0.00%
d__Bacteria	k__unclassified_d__Bacteria	p__Nitrospirae	c__Nitrospira	o__Nitrospirales	f__Nitrospiraceae	g__Leptospirillum	0	0.00%
d__Eukaryota	k__Viridiplantae	p__Streptophyta	c__unclassified_p__Streptophyta	o__Brassicales	f__Cleomaceae	g__Tarenaya	0	0.00%
d__Eukaryota	k__Viridiplantae	p__Streptophyta	c__unclassified_p__Streptophyta	o__Malpighiales	f__Euphorbiaceae	g__Ricinus	0	0.00%
d__Eukaryota	k__Viridiplantae	p__Streptophyta	c__unclassified_p__Streptophyta	o__Gentianales	f__Rubiaceae	g__Coffea	0	0.00%
d__Eukaryota	k__Viridiplantae	p__Streptophyta	c__unclassified_p__Streptophyta	o__Rosales	f__Rosaceae	g__Pyrus	0	0.00%
d__Eukaryota	k__Viridiplantae	p__Streptophyta	c__unclassified_p__Streptophyta	o__Proteales	f__Nelumbonaceae	g__Nelumbo	0	0.00%
d__Eukaryota	k__Viridiplantae	p__Streptophyta	c__unclassified_p__Streptophyta	o__Fagales	f__Betulaceae	g__Betula	0	0.00%
d__Eukaryota	k__Viridiplantae	p__Streptophyta	c__unclassified_p__Streptophyta	o__Malpighiales	f__Salicaceae	g__Salix	0	0.00%
d__Bacteria	k__unclassified_d__Bacteria	p__Bacteroidetes	c__Flavobacteriia	o__Flavobacteriales	f__Flavobacteriaceae	g__Gaetbulibacter	0	0.00%
d__Bacteria	k__unclassified_d__Bacteria	p__Proteobacteria	c__Epsilonproteobacteria	o__unclassified_c__Epsilonproteobacteria	f__unclassified_c__Epsilonproteobacteria	g__Sulfurovum	0	0.00%
d__Eukaryota	k__Viridiplantae	p__Streptophyta	c__unclassified_p__Streptophyta	o__Brassicales	f__Brassicaceae	g__Arabis	0	0.00%
d__Eukaryota	k__Viridiplantae	p__Streptophyta	c__unclassified_p__Streptophyta	o__Brassicales	f__Brassicaceae	g__Camelina	0	0.00%
d__Bacteria	k__unclassified_d__Bacteria	p__Proteobacteria	c__Deltaproteobacteria	o__Desulfarculales	f__Desulfarculaceae	g__Desulfocarbo	0	0.00%
d__Bacteria	k__unclassified_d__Bacteria	p__Cyanobacteria	c__unclassified_p__Cyanobacteria	o__Chroococcales	f__unclassified_o__Chroococcales	g__Crocosphaera	0	0.00%
d__Bacteria	k__unclassified_d__Bacteria	p__Actinobacteria	c__Actinobacteria	o__Micrococcales	f__Micrococcaceae	g__Paenarthrobacter	0	0.00%
d__Eukaryota	k__Viridiplantae	p__Streptophyta	c__unclassified_p__Streptophyta	o__Fabales	f__Fabaceae	g__Cajanus	0	0.00%
d__Bacteria	k__unclassified_d__Bacteria	p__unclassified_d__Bacteria	c__unclassified_d__Bacteria	o__unclassified_d__Bacteria	f__unclassified_d__Bacteria	g__Caldithrix	0	0.00%
d__Bacteria	k__unclassified_d__Bacteria	p__Bacteroidetes	c__Bacteroidia	o__Bacteroidales	f__Prolixibacteraceae	g__Draconibacterium	0	0.00%
d__Eukaryota	k__Viridiplantae	p__Streptophyta	c__unclassified_p__Streptophyta	o__Rosales	f__Rosaceae	g__Malus	0	0.00%
d__Eukaryota	k__Viridiplantae	p__Streptophyta	c__Liliopsida	o__Zingiberales	f__Musaceae	g__Musa	0	0.00%
d__Bacteria	k__unclassified_d__Bacteria	p__Firmicutes	c__Erysipelotrichia	o__Erysipelotrichales	f__Erysipelotrichaceae	g__Catenibacterium	0	0.00%
d__Eukaryota	k__Viridiplantae	p__Streptophyta	c__unclassified_p__Streptophyta	o__Rosales	f__Rosaceae	g__Fragaria	0	0.00%
d__Eukaryota	k__Viridiplantae	p__Streptophyta	c__unclassified_p__Streptophyta	o__Asterales	f__Asteraceae	g__Cynara	0	0.00%
d__Bacteria	k__unclassified_d__Bacteria	p__Fusobacteria	c__Fusobacteriia	o__Fusobacteriales	f__Fusobacteriaceae	g__Psychrilyobacter	0	0.00%
d__Bacteria	k__unclassified_d__Bacteria	p__Candidatus_Omnitrophica	c__unclassified_p__Candidatus_Omnitrophica	o__unclassified_p__Candidatus_Omnitrophica	f__unclassified_p__Candidatus_Omnitrophica	g__unclassified_p__Candidatus_Omnitrophica	0	0.00%
d__Eukaryota	k__Viridiplantae	p__Streptophyta	c__unclassified_p__Streptophyta	o__Cucurbitales	f__Cucurbitaceae	g__Cucumis	0	0.00%
d__Bacteria	k__unclassified_d__Bacteria	p__Proteobacteria	c__Alphaproteobacteria	o__Rhodobacterales	f__Rhodobacteraceae	g__Tateyamaria	0	0.00%
d__Eukaryota	k__Viridiplantae	p__Streptophyta	c__Liliopsida	o__Arecales	f__Arecaceae	g__Phoenix	0	0.00%
d__Eukaryota	k__Viridiplantae	p__Streptophyta	c__unclassified_p__Streptophyta	o__Solanales	f__Solanaceae	g__Solanum	0	0.00%
d__Bacteria	k__unclassified_d__Bacteria	p__Bacteroidetes	c__Bacteroidia	o__Bacteroidales	f__Porphyromonadaceae	g__Paludibacter	0	0.00%
d__Bacteria	k__unclassified_d__Bacteria	p__Proteobacteria	c__Deltaproteobacteria	o__Myxococcales	f__Cystobacteraceae	g__Stigmatella	0	0.00%
d__Eukaryota	k__Viridiplantae	p__Streptophyta	c__unclassified_p__Streptophyta	o__Rosales	f__Ulmaceae	g__Ulmus	0	0.00%
d__Viruses	k__unclassified_d__Viruses	p__unclassified_d__Viruses	c__unclassified_d__Viruses	o__unclassified_d__Viruses	f__Retroviridae	g__Intracisternal_A-particles	0	0.00%
d__Eukaryota	k__Viridiplantae	p__Streptophyta	c__unclassified_p__Streptophyta	o__Rosales	f__Moraceae	g__Morus	0	0.00%
d__Eukaryota	k__Viridiplantae	p__Streptophyta	c__unclassified_p__Streptophyta	o__Malvales	f__Malvaceae	g__Theobroma	0	0.00%
d__Viruses	k__unclassified_d__Viruses	p__unclassified_d__Viruses	c__unclassified_d__Viruses	o__unclassified_d__Viruses	f__Retroviridae	g__Gammaretrovirus	0	0.00%
d__Eukaryota	k__Viridiplantae	p__Streptophyta	c__unclassified_p__Streptophyta	o__Rosales	f__Rosaceae	g__Prunus	0	0.00%
d__Eukaryota	k__unclassified_d__Eukaryota	p__unclassified_d__Eukaryota	c__unclassified_d__Eukaryota	o__Kinetoplastida	f__Trypanosomatidae	g__Trypanosoma	0	0.00%
d__Bacteria	k__unclassified_d__Bacteria	p__Nitrospirae	c__unclassified_p__Nitrospirae	o__unclassified_p__Nitrospirae	f__unclassified_p__Nitrospirae	g__unclassified_p__Nitrospirae	0	0.00%
							83777510	

Table S3. CAZymes in TMC7 metagenome.

Family	Gene_coun	Reads_nun	Description
AA2	1	1240	manganese peroxidase (EC 1.11.1.13); versatile peroxidase (EC 1.11.1.16); lignin peroxidase (EC 1.11.1.14); peroxidase (EC 1.11.1.-)
AA4	15	56066	vanillyl-alcohol oxidase (EC 1.1.3.38)
AA6	39	66334	1,4-benzoquinone reductase (EC. 1.6.5.6)
AA7	2	1608	glucooligosaccharide oxidase (EC 1.1.3.-); chitooligosaccharide oxidase (EC 1.1.3.-)
CBM12	1	1294	Modules of approx. 40-60 residues. The majority of these modules is found among chitinases where the function is chitin-binding. Distantly related to the CBM5 family.
CBM16	9	52702	Carbohydrate-binding module 16. Binding to cellulose and glucomannan demonstrated [B. Bae et al (2008) J Biol Chem. 283:12415-25 (PMID: 18025086)]
CBM20	2	12	The granular starch-binding function has been demonstrated in several cases. Interact strongly with cyclodextrins. Often designated as starch-binding domains (SBD).
CBM23	1	108	Mannan-binding function demonstrated in one case.
CBM25	7	12480	Starch-binding function demonstrated in one case.
CBM3	7	28046	Modules of approx. 150 residues found in bacterial enzymes. The cellulose-binding function has been demonstrated in many cases. In one instance binding to chitin has been reported.
CBM31	2	1040	Binding to beta-1,3-xylan has been demonstrated for the C-terminal module of the beta-1,3-xylanase of Alcaligenes sp. XY234.
CBM32	3	4634	Binding to galactose and lactose has been demonstrated for the module of Micromonospora viridifaciens sialidase (PMID: 16239725). Binding to polygalacturonic acid has been shown for a Yersinia member (PMID: 17292916). Binding to LacNAc (beta-D-galactosyl-1,4-beta-D-N-acetylglucosamine) has been shown for an N-acetylglucosaminidase from Clostridium perfingens (PMID: 16990278).
CBM34	1	14	Modules of approx. 120 residues. Granular starch-binding function has been demonstrated in the case of Thermoactinomyces vulgaris R-47α-amylase 1 (TVAI).
CBM35	6	24400	Modules of approx. 130 residues. A module that is conserved in three Cellvibrio xylan-degrading enzymes binds to xylan and the interaction is calcium dependent, while a module from a Cellvibrio mannanase binds to decorated soluble mannans and mannooligosaccharides. A module in a
CBM37	10	33850	Modules of approx. 100 residues, conserved in numerous R. albus polysaccharide-degrading enzymes and other proteins from this bacterium. Several members of CBM37 have been shown to exhibit rather broad binding specificity to xylan, chitin, microcrystalline and phosphoric-acid swollen cellulose, as well as more heterogeneous substrates, such as alfalfa cell walls, banana stem and wheat straw.
CBM4	3	21588	Modules of approx. 150 residues found in bacterial enzymes. Binding of these modules has been demonstrated with xylan, beta-1,3-glucan, beta-1,3-1,4-glucan, beta-1,6-glucan and amorphous cellulose but not with crystalline cellulose.
CBM42	2	6504	Modules of approx. 160 residues found mostly at the C-terminus of GH54 catalytic domains. Binding to arabinofuranose (present in arabinoxylan) has been demonstrated.
CBM44	1	3110	The C-terminal CBM44 module of the Clostridium thermocellum enzyme has been demonstrated to bind equally well cellulose and xyloglucan
CBM46	2	204	Modules of approx. 100 residues, found at the C-terminus of several GH5 cellulases. Cellulose-binding function demonstrated in one case.
CBM48	1	4394	Modules of approx. 100 residues with glycogen-binding function, appended to GH13 modules. Also found in the beta subunit (glycogen-binding) of AMP-activated protein kinases (AMPK)
CBM50	129	239554	Modules of approx. 50 residues found attached to various enzymes from families GH18, GH19, GH23, GH24, GH25 and GH73, i.e. enzymes cleaving either chitin or peptidoglycan. Binding to chitopentaose demonstrated in the case of Pteris ryukyuensis chitinase A [Ohnuma T et al. (2008) J. Biol. Chem. 283:5178-87 (PMID: 18083709)]. CBM50 modules are also found in a multitude of other enzymes targeting the petidoglycan such as peptidases and amidases. These enzymes are not reported in the list below.
CBM51	4	6940	Modules of approx. 150 residues found attached to various enzymes from families GH2, GH27, GH31, GH95, GH98 and GH101 . Binding to galactose and to blood group A/B-antigens demonstrated in the case of C. perfringens GH95CBM51 and GH98CBM51 respectively [Gregg KJ et al. (2008) J. Biol. Chem. 283:12604-13 PMID: 18292090].
CBM54	3	560	Binding to xylan, yeast cell wall glucan and chitin shown in Dvortsov et al., Microbiology UK (2009) in press.
CBM6	6	37160	Modules of approx. 120 residues. The cellulose-binding function has been demonstrated in one case on amorphous cellulose and beta-1,4-xylan. Some of these modules also bind beta-1,3-glucan, beta-1,3-1,4-glucan, and beta-1,4-glucan.
CBM61	5	38294	Modules of approx. 150 residues found appended to GH16, GH30, GH31, GH43, GH53 and GH66 catalytic domains. A beta-1,4-galactan binding function has been demonstrated for the CBM60 of Thermotoga maritima GH53 galactanase [PMID: 20826814].
CBM66	3	366	The CBM66 module, derived from the Bacillus subtilis exo-acting beta-fructosidase SacC, targets the terminal fructoside residue of fructans.
CBM67	4	146	Fujimoto et al. [PMID : 23486481] disclosed the L-rhamnose binding activity and 3-D structure of the CBM67 of Streptomyces avermitilis alpha-L-rhamnosidase (SaRha78A);
CBM68	1	350	Binding to maltotriose and maltotetraose shown for the pullulanase of Anoxybacillus sp. LM18-11. Binding function derived from crystal structure and deletion of the CBM, which showed reduced specific activity and increased Km value compared to the wild type enzyme.
CBM70	3	19088	The hyaluronan-specific binding function of the N-terminal CBM70 module of Streptococcus pneumoniae hyaluronate lyase has been demonstrated.
CBM77	2	1846	Pectin binding modules of approx. 110 residues. The Ruminococcus flavefaciens CBM77 was shown to bind various pectins of low degree of esterification.
CBM9	9	6772	Modules of approx. 170 residues found so far only in xylanases. The cellulose-binding function has been demonstrated in one case.
CE1	44	60674	acetyl xylan esterase (EC 3.1.1.72); cinnamoyl esterase (EC 3.1.1.-); feruloyl esterase (EC 3.1.1.73); carboxylesterase (EC 3.1.1.1); S-formylglutathione hydrolase (EC 3.1.2.12); diacylglycerol O-acyltransferase (EC 2.3.1.20); trehalose 6-O-mycolyltransferase (EC 2.3.1.122)
CE10	21	53606	arylesterase (EC 3.1.1.-); carboxyl esterase (EC 3.1.1.3); acetylcholinesterase (EC 3.1.1.7); cholinesterase (EC 3.1.1.8); sterol esterase (EC 3.1.1.13); brefeldin A esterase (EC 3.1.1.-).
CE12	11	26316	pectin acetylesterase (EC 3.1.1.-); rhamnogalacturonan acetylesterase (EC 3.1.1.-); acetyl xylan esterase (EC 3.1.1.72)
CE14	15	35886	N-acetyl-1-D-myo-inosityl-2-amino-2-deoxy-alpha-D-glucopyranoside deacetylase (EC 3.5.1.89); diacetylchitobiose deacetylase (EC 3.5.1.-); mycothiol S-conjugate amidase (EC 3.5.1.-)
CE15	2	68	4-O-methyl-glucuronoyl methylesterase (EC 3.1.1.-)
CE3	17	39112	acetyl xylan esterase (EC 3.1.1.72).
CE4	95	152940	acetyl xylan esterase (EC 3.1.1.72); chitin deacetylase (EC 3.5.1.41); chitooligosaccharide deacetylase (EC 3.5.1.-); peptidoglycan GlcNAc deacetylase (EC 3.5.1.-); peptidoglycan N-acetylmuramic acid deacetylase (EC 3.5.1.-).
CE6	1	978	acetyl xylan esterase (EC 3.1.1.72).
CE7	16	50096	acetyl xylan esterase (EC 3.1.1.72); cephalosporin-C deacetylase (EC 3.1.1.41).
CE8	8	16440	pectin methylesterase (EC 3.1.1.11).
CE9	20	47026	N-acetylglucosamine 6-phosphate deacetylase (EC 3.5.1.25); N-acetylglucosamine 6-phosphate deacetylase (EC 3.5.1.80)
GH1	29	49444	beta-glucosidase (EC 3.2.1.21); beta-galactosidase (EC 3.2.1.23); beta-mannosidase (EC 3.2.1.25); beta-glucuronidase (EC 3.2.1.31); beta-xylosidase (EC 3.2.1.37); beta-D-fucosidase (EC 3.2.1.38); phlorizin hydrolase (EC 3.2.1.62); exo-beta-1,4-glucanase (EC 3.2.1.74); 6-phospho-beta-galactosidase (EC 3.2.1.85); 6-phospho-beta-glucosidase (EC 3.2.1.86); strictosidine beta-glucosidase (EC 3.2.1.105); lactase (EC 3.2.1.108); amygdalin beta-glucosidase (EC 3.2.1.117); prunasin beta-glucosidase (EC 3.2.1.118); vicianin hydrolase (EC 3.2.1.119); raucaffricine beta-glucosidase (EC 3.2.1.125); thioglucosidase (EC 3.2.1.147); beta-primeverosidase (EC 3.2.1.149); isoflavonoid 7-O-beta-apiosyl-beta-glucosidase (EC 3.2.1.161); ABA-specific beta-glucosidase (EC 3.2.1.175); DIMBOA beta-glucosidase (EC 3.2.1.182); beta-glycosidase (EC 3.2.1.-); hydroxyisourate hydrolase (EC 3.-.-.-)
GH10	20	141490	endo-1,4-beta-xylanase (EC 3.2.1.8); endo-1,3-beta-xylanase (EC 3.2.1.32); tomatinase (EC 3.2.1.-); xylan endotransglycosylase (EC 2.4.2.-)
GH105	10	49172	unsaturated rhamnogalacturonyl hydrolase (EC 3.2.1.172); d-4,5-unsaturated beta-glucuronyl hydrolase (EC 3.2.1.-)
GH106	8	55788	alpha-L-rhamnosidase (EC 3.2.1.40)
GH109	77	122594	alpha-N-acetylgalactosaminidase (EC 3.2.1.49)
GH11	1	7380	endo-beta-1,4-xylanase (EC 3.2.1.8); endo-beta-1,3-xylanase (EC 3.2.1.32)
GH112	1	16536	lacto-N-biose phosphorylase or galacto-N-biose phosphorylase (EC 2.4.1.211); D-galactosyl-beta-1,4-L-rhamnose phosphorylase (EC 2.4.1.247); galacto-N-biose/lacto-N-biose phosphorylase (EC 2.4.1.-)
GH113	1	1470	beta-mannanase (EC 3.2.1.78)
GH114	1	946	endo-alpha-1,4-polygalactosaminidase (EC 3.2.1.109)
GH115	1	12512	xylan alpha-1,2-glucuronidase (3.2.1.131); alpha-(4-O-methyl)-glucuronidase (3.2.1.-)
GH116	1	1236	beta-glucosidase (EC 3.2.1.21); beta-xylosidase (EC 3.2.1.37); acid beta-glucosidase/beta-glucosylceramidase (EC 3.2.1.45); beta-N-acetylglucosaminidase (EC 3.2.1.52)
GH120	5	17812	beta-xylosidase (EC 3.2.1.37)
GH124	1	3652	endoglucanase (EC 3.2.1.4)
GH125	1	12	exo-alpha-1,6-mannosidase (EC 3.2.1.-)
GH126	2	4994	alpha-amylase (EC 3.2.1.-)
GH127	3	31670	beta-L-arabinofuranosidase (EC 3.2.1.185); 3-C-carboxy-5-deoxy-L-xylose (aceric acid) hydrolase
GH129	3	3426	alpha-N-acetylgalactosaminidase (EC 3.2.1.49);
GH13	5	43888	alpha-amylase (EC 3.2.1.1); pullulanase (EC 3.2.1.41); cyclomaltodextrin glucanotransferase (EC 2.4.1.19); cyclomaltodextrinase (EC 3.2.1.54); trehalose-6-phosphate hydrolase (EC 3.2.1.93); oligo-alpha-glucosidase (EC 3.2.1.10); maltogenic amylase (EC 3.2.1.133); neopullulanase (EC 3.2.1.135); alpha-glucosidase (EC 3.2.1.20); maltotetraose-forming alpha-amylase (EC 3.2.1.60); isoamylase (EC 3.2.1.68); glucodextranase (EC 3.2.1.70); maltohexaose-forming alpha-amylase (EC 3.2.1.98); maltotriose-forming alpha-amylase (EC 3.2.1.116); branching enzyme (EC 2.4.1.18); trehalose synthase (EC 5.4.99.16); 4-alpha-glucanotransferase (EC 2.4.1.25); maltopentaose-forming alpha-amylase (EC 3.2.1.-) ; amylosucrase (EC 2.4.1.4) ; sucrose phosphorylase (EC 2.4.1.7); malto-oligosyltrehalose trehalohydrolase (EC 3.2.1.141); isomaltulose synthase (EC 5.4.99.11); malto-oligosyltrehalose synthase (EC 5.4.99.15); amylo-alpha-1,6-glucosidase (EC 3.2.1.33); alpha-1,4-glucan: phosphate alpha-maltosyltransferase (EC 2.4.99.16); 6'-P-su
GH130	24	54076	beta-1,4-mannosylglucose phosphorylase (EC 2.4.1.281); beta-1,4-mannooligosaccharide phosphorylase (EC 2.4.1.319); beta-1,4-mannosyl-N-acetyl-glucosamine phosphorylase (EC 2.4.1.320); beta-1,2-mannobiose phosphorylase (EC 2.4.1.-); beta-1,2-oligomannan phosphorylase (EC 2.4.1.-); beta-1,2-mannosidase (EC 3.2.1.-)
GH133	5	27784	amylo-alpha-1,6-glucosidase (EC 3.2.1.33);
GH137	2	38	beta-L-arabinofuranosidase (EC 3.2.1.185)
GH138	2	2772	alpha-galacturonidase (EC 3.2.1.-)
GH13_1	1	5916	GH13's subfamily
GH13_13	3	10168	GH13's subfamily
GH13_18	4	17168	GH13's subfamily
GH13_2	3	11756	GH13's subfamily
GH13_20	9	53178	GH13's subfamily
GH13_21	1	12334	GH13's subfamily
GH13_29	3	7808	GH13's subfamily
GH13_31	8	21372	GH13's subfamily
GH13_9	8	38026	GH13's subfamily
GH140	5	19678	endo-apiosidase (EC 3.2.1.-)
GH141	1	9140	alpha-L-fucosidase (EC 3.2.1.51)
GH142	1	56	beta-L-arabinofuranosidase (EC 3.2.1.185)
GH143	2	62	2-keto-3-deoxy-D-lyxo-heptulosaric acid-hydrolase (EC 3.2.1.-)
GH15	5	35152	glucoamylase (EC 3.2.1.3); glucodextranase (EC 3.2.1.70); alpha,alpha-trehalase (EC 3.2.1.28); dextran dextrinase (EC 2.4.1.2)
GH16	3	4480	xyloglucan:xyloglucosyltransferase (EC 2.4.1.207); keratan-sulfate endo-1,4-beta-galactosidase (EC 3.2.1.103); endo-1,3-beta-glucanase (EC 3.2.1.39); endo-1,3(4)-beta-glucanase (EC 3.2.1.6); licheninase (EC 3.2.1.73); beta-agarase (EC 3.2.1.81); kappa;-carrageenase (EC 3.2.1.83); xyloglucanase (EC 3.2.1.151); endo-beta-1,3-galactanase (EC 3.2.1.181); beta-porphyranase (EC 3.2.1.178); hyaluronidase (EC 3.2.1.35); endo-beta-1,4-galactosidase (EC 3.2.1.-); chitin beta-1,6-glucanosyltransferase (EC 2.4.1.-); endo-beta-1,4-galactosidase (EC 3.2.1.-)
GH18	46	156032	chitinase (EC 3.2.1.14); lysozyme (EC 3.2.1.17); endo-beta-N-acetylglucosaminidase (EC 3.2.1.96); peptidoglycan hydrolase with endo-beta-N-acetylglucosaminidase specificity (EC 3.2.1.-); Nod factor hydrolase (EC 3.2.1.-); xylanase inhibitor; concanavalin B; narbonin
GH2	25	168714	beta-galactosidase (EC 3.2.1.23) ; beta-mannosidase (EC 3.2.1.25); beta-glucuronidase (EC 3.2.1.31); alpha-L-arabinofuranosidase (EC 3.2.1.55); mannosylglycoprotein endo-beta-mannosidase (EC 3.2.1.152); exo-beta-glucosaminidase (EC 3.2.1.165)
GH20	1	264	beta-hexosaminidase (EC 3.2.1.52); lacto-N-biosidase (EC 3.2.1.140); beta-1,6-N-acetylglucosaminidase) (EC 3.2.1.-); beta-6-SO3-N-acetylglucosaminidase (EC 3.2.1.-)
GH23	30	56072	lysozyme type G (EC 3.2.1.17); peptidoglycan lyase (EC 4.2.2.n1) also known in the literature as peptidoglycan lytic transglycosylase; chitinase (EC 3.2.1.14)
GH25	2	12	lysozyme (EC 3.2.1.17)
GH26	7	59378	beta-mannanase (EC 3.2.1.78); exo-beta-1,4-mannobiohydrolase (EC 3.2.1.100); beta-1,3-xylanase (EC 3.2.1.32); lichenase / endo-beta-1,3-1,4-glucanase (EC 3.2.1.73); mannobiose-producing exo-beta-mannanase (EC 3.2.1.-)
GH27	1	8546	alpha-galactosidase (EC 3.2.1.22); alpha-N-acetylgalactosaminidase (EC 3.2.1.49); isomalto-dextranase (EC 3.2.1.94); beta-L-arabinopyranosidase (EC 3.2.1.88); galactan:galactan galactosyltransferase (EC 2.4.1.-)
GH28	5	28470	polygalacturonase (EC 3.2.1.15); alpha-L-rhamnosidase (EC 3.2.1.40); exo-polygalacturonase (EC 3.2.1.67); exo-polygalacturonosidase (EC 3.2.1.82); rhamnogalacturonase (EC 3.2.1.171); rhamnogalacturonan alpha-1,2-galacturonohydrolase (EC 3.2.1.173); endo-xylogalacturonan hydrolase (EC 3.2.1.-)
GH29	6	25280	alpha-L-fucosidase (EC 3.2.1.51); alpha-1,3/1,4-L-fucosidase (EC 3.2.1.111)
GH3	29	100644	beta-glucosidase (EC 3.2.1.21); xylan 1,4-beta-xylosidase (EC 3.2.1.37); beta-glucosylceramidase (EC 3.2.1.45); beta-N-acetylhexosaminidase (EC 3.2.1.52); alpha-L-arabinofuranosidase (EC 3.2.1.55); glucan 1,3-beta-glucosidase (EC 3.2.1.58); glucan 1,4-beta-glucosidase (EC 3.2.1.74); isoprimeverose-producing oligoxyloglucan hydrolase (EC 3.2.1.120); coniferin beta-glucosidase (EC 3.2.1.126); exo-1,3-1,4-glucanase (EC 3.2.1.-); beta-N-acetylglucosaminide phosphorylases (EC 2.4.1.-)
GH30_8	1	4114	GH30's subfamily
GH31	14	47634	alpha-glucosidase (EC 3.2.1.20); alpha-galactosidase (EC 3.2.1.22); alpha-mannosidase (EC 3.2.1.24); alpha-1,3-glucosidase (EC 3.2.1.84); sucrase-isomaltase (EC 3.2.1.48) (EC 3.2.1.10); alpha-xylosidase (EC 3.2.1.177); alpha-glucan lyase (EC 4.2.2.13); isomaltosyltransferase (EC 2.4.1.-); oligosaccharide alpha-1,4-glucosyltransferase (EC 2.4.1.161); sulfoquinovosidase (EC 3.2.1.-)
GH32	5	9698	invertase (EC 3.2.1.26); endo-inulinase (EC 3.2.1.7); beta-2,6-fructan 6-levanbiohydrolase (EC 3.2.1.64); endo-levanase (EC 3.2.1.65); exo-inulinase (EC 3.2.1.80); fructan beta-(2,1)-fructosidase/1-exohydrolase (EC 3.2.1.153); fructan beta-(2,6)-fructosidase/6-exohydrolase (EC 3.2.1.154); sucrose:sucrose 1-fructosyltransferase (EC 2.4.1.99); fructan:fructan 1-fructosyltransferase (EC 2.4.1.100); sucrose:fructan 6-fructosyltransferase (EC 2.4.1.10); fructan:fructan 6G-fructosyltransferase (EC 2.4.1.243); levan fructosyltransferase (EC 2.4.1.-); [retaining[sucrose:sucrose 6-fructosyltransferase (6-SST) (EC 2.4.1.-); cycloinulo-oligosaccharide fructanotransferase (EC 2.4.1.-)
GH33	2	72	sialidase or neuraminidase (EC 3.2.1.18); trans-sialidase (EC 2.4.1.-); 2-keto-3-deoxynononic acid hydrolase (EC 3.2.1.-); anhydrosialidase (EC 4.2.2.15); 3-deoxy-D-manno-octulosonic-acid hydrolase (EC 3.2.1.-)
GH35	2	16812	beta-galactosidase (EC 3.2.1.23); exo-beta-glucosaminidase (EC 3.2.1.165); exo-beta-1,4-galactanase (EC 3.2.1.-); beta-1,3-galactosidase (EC 3.2.1.-)
GH36	11	61974	alpha-galactosidase (EC 3.2.1.22); alpha-N-acetylgalactosaminidase (EC 3.2.1.49); stachyose synthase (EC 2.4.1.67); raffinose synthase (EC 2.4.1.82)
GH37	1	11212	alpha,alpha-trehalase (EC 3.2.1.28).
GH38	8	26766	alpha-mannosidase (EC 3.2.1.24); mannosyl-oligosaccharide alpha-1,2-mannosidase (EC 3.2.1.113); mannosyl-oligosaccharide alpha-1,3-1,6-mannosidase (EC 3.2.1.114); alpha-2-O-mannosylglycerate hydrolase (EC 3.2.1.170); mannosyl-oligosaccharide alpha-1,3-mannosidase (EC 3.2.1.-)
GH39	6	38952	alpha-L-iduronidase (EC 3.2.1.76); beta-xylosidase (EC 3.2.1.37).
GH4	22	47864	maltose-6-phosphate glucosidase (EC 3.2.1.122); alpha-glucosidase (EC 3.2.1.20); alpha-galactosidase (EC 3.2.1.22); 6-phospho-beta-glucosidase (EC 3.2.1.86); alpha-glucuronidase (EC 3.2.1.139); alpha-galacturonase (EC 3.2.1.67); palatinase (EC 3.2.1.-)
GH42	8	15742	beta-galactosidase (EC 3.2.1.23); alpha-L-arabinopyranosidase (EC 3.2.1.-)
GH43_1	1	650	GH43's subfamily
GH43_10	5	48654	GH43's subfamily
GH43_11	1	900	GH43's subfamily
GH43_12	4	13700	GH43's subfamily
GH43_17	1	7506	GH43's subfamily
GH43_2	1	1962	GH43's subfamily
GH43_20	1	9342	GH43's subfamily
GH43_22	1	1598	GH43's subfamily
GH43_24	1	4988	GH43's subfamily
GH43_26	2	13476	GH43's subfamily
GH43_27	1	2310	GH43's subfamily
GH43_29	1	6278	GH43's subfamily
GH43_35	2	11986	GH43's subfamily
GH43_4	2	16716	GH43's subfamily
GH44	1	11206	endoglucanase (EC 3.2.1.4); xyloglucanase (EC 3.2.1.151)
GH48	3	20574	reducing end-acting cellobiohydrolase (EC 3.2.1.176); endo-beta-1,4-glucanase (EC 3.2.1.4); chitinase (EC 3.2.1.14)
GH5	2	10780	endo-beta-1,4-glucanase / cellulase (EC 3.2.1.4); endo-beta-1,4-xylanase (EC 3.2.1.8); beta-glucosidase (EC 3.2.1.21); beta-mannosidase (EC 3.2.1.25); beta-glucosylceramidase (EC 3.2.1.45); glucan beta-1,3-glucosidase (EC 3.2.1.58); licheninase (EC 3.2.1.73); exo-beta-1,4-glucanase / cellodextrinase (EC 3.2.1.74); glucan endo-1,6-beta-glucosidase (EC 3.2.1.75); mannan endo-beta-1,4-mannosidase (EC 3.2.1.78); cellulose beta-1,4-cellobiosidase (EC 3.2.1.91); steryl beta-glucosidase (EC 3.2.1.104); endoglycoceramidase (EC 3.2.1.123); chitosanase (EC 3.2.1.132); beta-primeverosidase (EC 3.2.1.149); xyloglucan-specific endo-beta-1,4-glucanase (EC 3.2.1.151); endo-beta-1,6-galactanase (EC 3.2.1.164); hesperidin 6-O-alpha-L-rhamnosyl-beta-glucosidase (EC 3.2.1.168); beta-1,3-mannanase (EC 3.2.1.-); arabinoxylan-specific endo-beta-1,4-xylanase (EC 3.2.1.-); mannan transglycosylase (EC 2.4.1.-)
GH51	8	32728	endoglucanase (EC 3.2.1.4); endo-beta-1,4-xylanase (EC 3.2.1.8); beta-xylosidase (EC 3.2.1.37); alpha-L-arabinofuranosidase (EC 3.2.1.55)
GH52	1	11314	beta-xylosidase (EC 3.2.1.37).
GH53	5	46350	endo-beta-1,4-galactanase (EC 3.2.1.89).
GH57	2	666	alpha-amylase (EC 3.2.1.1); alpha-galactosidase (EC 3.2.1.22); amylopullulanase (EC 3.2.1.41); cyclomaltodextrinase (EC 3.2.1.54); branching enzyme (EC 2.4.1.18); 4-alpha-glucanotransferase (EC 2.4.1.25)
GH5_1	4	22024	GH5's subfamily
GH5_19	1	11092	GH5's subfamily
GH5_2	1	1036	GH5's subfamily
GH5_22	1	2364	GH5's subfamily
GH5_25	1	7732	GH5's subfamily
GH5_34	1	9682	GH5's subfamily
GH5_35	1	1500	GH5's subfamily
GH5_36	1	6806	GH5's subfamily
GH5_37	2	5344	GH5's subfamily
GH5_4	1	6512	GH5's subfamily
GH65	4	2042	alpha,alpha-trehalase (EC 3.2.1.28); maltose phosphorylase (EC 2.4.1.8); trehalose phosphorylase (EC 2.4.1.64); kojibiose phosphorylase (EC 2.4.1.230); trehalose-6-phosphate phosphorylase (EC 2.4.1.216); nigerose phosphorylase (EC 2.4.1.279); 3-O-alpha-glucopyranosyl-L-rhamnose phosphorylase (EC 2.4.1.282); 2-O-alpha-glucopyranosylglycerol: phosphate beta-glucosyltransferase (EC 2.4.1.-); alpha-glucosyl-1,2-beta-galactosyl-L-hydroxylysine alpha-glucosidase (EC 3.2.1.107)
GH67	4	29962	alpha-glucuronidase (EC 3.2.1.139); xylan alpha-1,2-glucuronidase (EC 3.2.1.131)
GH73	18	44086	lysozyme (EC 3.2.1.17); mannosyl-glycoprotein endo-beta-N-acetylglucosaminidase (EC 3.2.1.96); peptidoglycan hydrolase with endo-beta-N-acetylglucosaminidase specificity (EC 3.2.1.-)
GH74	7	40172	endoglucanase (EC 3.2.1.4); oligoxyloglucan reducing end-specific cellobiohydrolase (EC 3.2.1.150); xyloglucanase (EC 3.2.1.151)
GH76	7	17582	alpha-1,6-mannanase (EC 3.2.1.101)
GH77	2	1640	amylomaltase or 4-alpha-glucanotransferase (EC 2.4.1.25)
GH78	3	156	alpha-L-rhamnosidase (EC 3.2.1.40); rhamnogalacturonan alpha-L-rhamnohydrolase (EC 3.2.1.174)
GH8	1	4926	chitosanase (EC 3.2.1.132); cellulase (EC 3.2.1.4); licheninase (EC 3.2.1.73); endo-1,4-beta-xylanase (EC 3.2.1.8); reducing-end-xylose releasing exo-oligoxylanase (EC 3.2.1.156)
GH81	1	6332	endo-beta-1,3-glucanase (EC 3.2.1.39)
GH88	2	9944	d-4,5-unsaturated beta-glucuronyl hydrolase (EC 3.2.1.-)
GH9	16	100654	endoglucanase (EC 3.2.1.4); endo-beta-1,3(4)-glucanase / lichenase-laminarinase (EC 3.2.1.6); beta-glucosidase (EC 3.2.1.21); lichenase / endo-beta-1,3-1,4-glucanase (EC 3.2.1.73); exo-beta-1,4-glucanase / cellodextrinase (EC 3.2.1.74); cellobiohydrolase (EC 3.2.1.91); xyloglucan-specific endo-beta-1,4-glucanase / endo-xyloglucanase (EC 3.2.1.151); exo-beta-glucosaminidase (EC 3.2.1.165)
GH94	15	127136	cellobiose phosphorylase (EC 2.4.1.20); laminaribiose phosphorylase (EC 2.4.1.31); cellodextrin phosphorylase (EC 2.4.1.49); chitobiose phosphorylase (EC 2.4.1.-); cyclic beta-1,2-glucan synthase (EC 2.4.1.-); cellobionic acid phosphorylase (EC 2.4.1.321); beta-1,2-oligoglucan phosphorylase (EC 2.4.1.-)
GH95	11	44410	alpha-L-fucosidase (EC 3.2.1.51); alpha-1,2-L-fucosidase (EC 3.2.1.63); alpha-L-galactosidase (EC 3.2.1.-)
GT1	5	14606	UDP-glucuronosyltransferase (EC 2.4.1.17); zeatin O-beta-xylosyltransferase (EC 2.4.2.40); 2-hydroxyacylsphingosine 1-beta-galactosyltransferase (EC 2.4.1.45); N-acylsphingosine galactosyltransferase (EC 2.4.1.47); flavonol 3-O-glucosyltransferase (EC 2.4.1.91); anthocyanidin 3-O-glucosyltransferase (EC 2.4.1.115); sinapate 1-glucosyltransferase (EC 2.4.1.120); indole-3-acetate beta-glucosyltransferase (EC 2.4.1.121); flavonol L-rhamnosyltransferase (EC 2.4.1.159); sterol glucosyltransferase (EC 2.4.1.173); UDP-Glc: 4-hydroxybenzoate 4-O-beta-glucosyltransferase (EC 2.4.1.194); zeatin O-beta-glucosyltransferase (EC 2.4.1.203); limonoid glucosyltransferase (EC 2.4.1.210); UDP-GlcA: baicalein 7-O-beta-glucuronosyltransferase (EC 2.4.1.253); UDP-Glc: chalcone 4'-O-beta-glucosyltransferase (EC 2.4.1.286); ecdysteroid UDP-glucosyltransferase (EC 2.4.1.-); salicylic acid beta-glucosyltransferase (EC 2.4.1.-); anthocyanin 3-O-galactosyltransferase (EC 2.4.1.-); anthocyanin 5-O-glucosyltransferase (EC 2.4.1.-); dTD
GT10	1	970	galactoside alpha-1,3/1,4-L-fucosyltransferase (EC 2.4.1.65); galactoside alpha-1,3-L-fucosyltransferase (EC 2.4.1.152); glycoprotein alpha-1,3-L-fucosyltransferase (EC 2.4.1.214)
GT19	9	15824	lipid-A-disaccharide synthase (EC 2.4.1.182).
GT2	119	256614	cellulose synthase (EC 2.4.1.12); chitin synthase (EC 2.4.1.16); dolichyl-phosphate beta-D-mannosyltransferase (EC 2.4.1.83); dolichyl-phosphate beta-glucosyltransferase (EC 2.4.1.117); N-acetylglucosaminyltransferase (EC 2.4.1.-); N-acetylgalactosaminyltransferase (EC 2.4.1.-); hyaluronan synthase (EC 2.4.1.212); chitin oligosaccharide synthase (EC 2.4.1.-); beta-1,3-glucan synthase (EC 2.4.1.34); beta-1,4-mannan synthase (EC 2.4.1.-); beta-mannosylphosphodecaprenol-mannooligosaccharide alpha-1,6-mannosyltransferase (EC 2.4.1.199); UDP-Galf: rhamnopyranosyl-N-acetylglucosaminyl-PP-decaprenol beta-1,4/1,5-galactofuranosyltransferase (EC 2.4.1.287); UDP-Galf: galactofuranosyl-galactofuranosyl-rhamnosyl-N-acetylglucosaminyl-PP-decaprenol beta-1,5/1,6-galactofuranosyltransferase (EC 2.4.1.288); dTDP-L-Rha: N-acetylglucosaminyl-PP-decaprenol alpha-1,3-L-rhamnosyltransferase (EC 2.4.1.289)
GT23	12	37582	N-acetyl-beta-D-glucosaminide alpha-1,6-L-fucosyltransferase (EC 2.4.1.68); chitin-oligosaccharide alpha-1,6-L-fucosyltransferase (EC 2.4.1.-)
GT26	19	30816	UDP-ManNAcA: beta-N-acetyl mannosaminuronyltransferase (EC 2.4.1.-); UDP-ManNAc: beta-N-acetyl-mannosaminyltransferase (EC 2.4.1.-); UDP-Glc: beta-1,4-glucosyltransferase (EC 2.4.1.-); beta-1,4-galactosyltransferase (EC 2.4.1.-)
GT27	1	806	polypeptide alpha-N-acetylgalactosaminyltransferase (EC 2.4.1.41)
GT28	40	79624	1,2-diacylglycerol 3-beta-galactosyltransferase (EC 2.4.1.46); 1,2-diacylglycerol 3-beta-glucosyltransferase (EC 2.4.1.157); UDP-GlcNAc: Und-PP-MurAc-pentapeptide beta-N-acetylglucosaminyltransferase (EC 2.4.1.227); digalactosyldiacylglycerol synthase (EC 2.4.1.241)
GT32	2	10616	alpha-1,6-mannosyltransferase (EC 2.4.1.-); alpha-1,4-N-acetylglucosaminyltransferase (EC 2.4.1.-); alpha-1,4-N-acetylgalactosaminyltransferase (EC 2.4.1.-);
GT34	1	56	UDP-Gal: galactomannan alpha-1,6-galactosyltransferase (EC 2.4.1.-); UDP-Xyl: xyloglucan alpha-1,6-xylosyltransferase (EC 2.4.2.39); alpha-1,2-galactosyltransferase (EC 2.4.1.-)
GT35	12	62474	glycogen or starch phosphorylase (EC 2.4.1.1).
GT39	5	13086	Dol-P-Man: protein alpha-mannosyltransferase (EC 2.4.1.109)
GT4	129	226144	sucrose synthase (EC 2.4.1.13); sucrose-phosphate synthase (EC 2.4.1.14); alpha-glucosyltransferase (EC 2.4.1.52); lipopolysaccharide N-acetylglucosaminyltransferase (EC 2.4.1.56); phosphatidylinositol alpha-mannosyltransferase (EC 2.4.1.57); GDP-Man: Man1GlcNAc2-PP-dolichol alpha-1,3-mannosyltransferase (EC 2.4.1.132); GDP-Man: Man3GlcNAc2-PP-dolichol/Man4GlcNAc2-PP-dolichol alpha-1,2-mannosyltransferase (EC 2.4.1.131); digalactosyldiacylglycerol synthase (EC 2.4.1.141); 1,2-diacylglycerol 3-glucosyltransferase (EC 2.4.1.157); diglucosyl diacylglycerol synthase (EC 2.4.1.208); trehalose phosphorylase (EC 2.4.1.231); NDP-Glc: alpha-glucose alpha-glucosyltransferase / alpha,alpha-trehalose synthase (EC 2.4.1.245); GDP-Man: Man2GlcNAc2-PP-dolichol alpha-1,6-mannosyltransferase (EC 2.4.1.257); UDP-GlcNAc: 2-deoxystreptamine alpha-N-acetylglucosaminyltransferase (EC 2.4.1.283); UDP-GlcNAc: ribostamycin alpha-N-acetylglucosaminyltransferase (EC 2.4.1.285); UDP-Gal alpha-galactosyltransferase (EC 2.4.1.-); UDP-Xyl
GT44	1	3434	UDP-Glc: alpha-glucosyltransferase (EC 2.4.1.-); UDP-GlcNAc: alpha-N-acetylglucosaminyltransferase (EC 2.4.1.-).
GT45	1	3864	alpha-N-acteylglucosaminyltransferase (EC 2.4.1.-)
GT46	3	2458	Deleted
GT5	14	54002	UDP-Glc: glycogen glucosyltransferase (EC 2.4.1.11); ADP-Glc: starch glucosyltransferase (EC 2.4.1.21); NDP-Glc: starch glucosyltransferase (EC 2.4.1.242); UDP-Glc: alpha-1,3-glucan synthase (EC 2.4.1.183) UDP-Glc: alpha-1,4-glucan synthase (EC 2.4.1.-)
GT51	30	92952	murein polymerase (EC 2.4.1.129).
GT70	2	7512	UDP-GlcA: beta-glucuronosyltransferase (EC 2.4.1.17)
GT76	3	11522	Dol-P-Man: alpha-1,6-mannosyltransferase (EC 2.4.1.-)
GT8	9	12120	lipopolysaccharide alpha-1,3-galactosyltransferase (EC 2.4.1.44); UDP-Glc: (glucosyl)lipopolysaccharide alpha-1,2-glucosyltransferase (EC 2.4.1.-); lipopolysaccharide glucosyltransferase 1 (EC 2.4.1.58); glycogenin glucosyltransferase (EC 2.4.1.186); inositol 1-alpha-galactosyltransferase (galactinol synthase) (EC 2.4.1.123); homogalacturonan alpha-1,4-galacturonosyltransferase (EC 2.4.1.43); UDP-GlcA: xylan alpha-glucuronyltransferase (EC 2.4.1.-)
GT81	13	15628	NDP-Glc: glucosyl-3-phosphoglycerate synthase (EC 2.4.1.-); NDP-Man: mannosyl-3-phosphoglycerate synthase (EC 2.4.1.-);
GT83	8	44090	undecaprenyl phosphate-alpha-L-Ara4N: 4-amino-4-deoxy-beta-L-arabinosyltransferase (EC 2.4.2.43); dodecaprenyl phosphate-beta-galacturonic acid: lipopolysaccharide core alpha-galacturonosyl transferase (EC 2.4.1.-)
GT84	1	140	cyclic beta-1,2-glucan synthase (EC 2.4.1.-);
GT9	2	1558	lipopolysaccharide N-acetylglucosaminyltransferase (EC 2.4.1.56); heptosyltransferase (EC 2.4.-.-).
GT94	3	6562	GDP-Man: GlcA-beta-1,2-Man-alpha-1,3-Glc-beta-1,4-Glc-alpha-1-PP-undecaprenol beta-1,4-mannosyltransferase (2.4.1.251)
PL1	9	6220	pectate lyase (EC 4.2.2.2); exo-pectate lyase (EC 4.2.2.9); pectin lyase (EC 4.2.2.10).
PL10	1	9648	pectate lyase (EC 4.2.2.2)
PL11	4	49112	rhamnogalacturonan endolyase (EC 4.2.2.23); rhamnogalacturonan exolyase (EC 4.2.2.24)
PL11_1	1	60	PL11's subfamily
PL15_2	1	372	PL15's subfamily
PL1_11	1	6306	PL1's subfamily
PL1_2	3	96	PL1's subfamily
PL1_5	1	5764	PL1's subfamily
PL2	2	96	pectate lyase (EC 4.2.2.2); exo-polygalacturonate lyase (EC 4.2.2.9).
PL22	2	8654	oligogalacturonate lyase / oligogalacturonide lyase (EC 4.2.2.6)
PL26	2	19682	rhamnogalacturonan exolyase (EC 4.2.2.24).
PL4	1	72	rhamnogalacturonan endolyase (EC 4.2.2.23).
PL9	7	47564	pectate lyase (EC 4.2.2.2); exopolygalacturonate lyase (EC 4.2.2.9); thiopeptidoglycan lyase (EC 4.2.2.-).
PL9_1	1	354	PL9's subfamily

Table S4. Calculation of contribution coefficients.

Reads number of CAZymes annoted as varies microbes (N g-f)

	Ruminiclostridium	Thermoanaerob	Geobacillus	Bacillaceae	Aeribacillus	Symbiobacterium	Desulfotomacul	Caldibacillus	Clostridium	others	Nf (total reads number of specific functi
LMEs											
AA2	0	0	0	0	0	1240	0	0	0	0	1240
AA4	0	8394	23988	6178	6996	910	1866	798	976	5960	56066
AA6	25030	16834	9082	6118	926	0	2330	0	1050	4964	66334
AA7	0	0	1560	0	0	0	0	0	0	48	1608
SUM	25030	25228	34630	12296	7922	2150	4196	798	2026	10972	125248
Endoglucanase											
GH5's subfamilies	61468	17898	0	1500	0	0	0	0	0	4006	84872
GH8	4926	0	0	0	0	0	0	0	0	0	4926
GH9	100654	0	0	0	0	0	0	0	0	0	100654
GH16	3800	0	0	0	0	0	0	0	0	680	4480
GH26	22686	34642	0	0	0	1066	984	0	0	0	59378
GH44	11206	0	0	0	0	0	0	0	0	0	11206
GH48	20574	0	0	0	0	0	0	0	0	0	20574
GH51	20722	9816	0	0	0	0	0	0	0	2190	32728
GH74	7144	28044	3514	0	0	1470	0	0	0	0	40172
GH81	6332	0	0	0	0	0	0	0	0	0	6332
SUM	259512	90400	3514	1500	0	2536	984	0	0	6876	365322
β-glucosidase											
GH1	8816	17598	13082	0	800	0	0	2428	1530	622	44876
GH3	82982	0	6554	0	1860	0	0	1162	926	1926	95410
GH5's subfamilies	61468	17898	0	1500	0	0	0	0	0	4006	84872
GH9	100654	0	0	0	0	0	0	0	0	0	100654
GH30_8	4114	0	0	0	0	0	0	0	0	0	4114
GH116	0	0	0	0	0	0	0	0	0	1236	1236
SUM	258034	35496	19636	1500	2660	0	0	3590	2456	7790	331162
exoglucanase											
GH5's subfamilies	61468	17898	0	1500	0	0	0	0	0	4006	84872
GH9	100654	0	0	0	0	0	0	0	0	0	100654
GH48	20574	0	0	0	0	0	0	0	0	0	20574
GH74	7144	28044	3514	0	0	1470	0	0	0	0	40172
SUM	189840	45942	3514	1500	0	1470	0	0	0	4006	246272
endo-β-1,4-xylanase 6GHs											
GH5's subfamilies	61468	17898	0	1500	0	0	0	0	0	4006	84872
GH8	4926	0	0	0	0	0	0	0	0	0	4926
GH10	90692	34650	0	0	0	0	0	0	2826	13322	141490
GH11	7380	0	0	0	0	0	0	0	0	0	7380
GH30_8	4114	0	0	0	0	0	0	0	0	0	4114
GH51	20722	9816	0	0	0	0	0	0	0	2190	32728
SUM	189302	62364	0	1500	0	0	0	0	2826	19518	275510
β-xylosidase 9GHs											
GH1	8816	17598	13082	0	800	0	0	2428	1530	5190	49444
GH3	82982	0	6554	0	1860	0	0	1162	926	7160	100644
GH30_8	4114	0	0	0	0	0	0	0	0	0	4114
GH39	25054	8480	0	0	0	0	0	0	0	5418	38952
GH43's subfamilies	120612	7506	0	0	0	0	0	900	0	11048	140066
GH51	20722	9816	0	0	0	0	0	0	0	2190	32728
GH52	0	11314	0	0	0	0	0	0	0	0	11314
GH116	0	0	0	0	0	0	0	0	0	1236	1236
GH120	0	12586	0	0	0	0	0	0	2394	2832	17812
SUM	262300	67300	19636	0	2660	0	0	4490	4850	35074	396310
β-mannosidase											
GH1	8816	17598	13082	0	800	0	0	2428	1530	5190	49444
GH2	120070	27188	8820	0	0	0	0	1340	2974	8322	168714
GH5's subfamilies	61468	17898	0	1500	0	0	0	0	0	4006	84872
SUM	190354	62684	21902	1500	800	0	0	3768	4504	17518	303030
arabinofuranosidases 6GHs											
GH2	120070	27188	8820	0	0	0	0	1340	2974	8322	168714
GH43's subfamilies	120612	7506	0	0	0	0	0	900	0	11048	140066
GH51	20722	9816	0	0	0	0	0	0	0	2190	32728
GH127	31670	0	0	0	0	0	0	0	0	0	31670
GH137	0	0	0	0	0	0	0	0	0	38	38
GH142	0	0	0	0	0	0	0	0	0	56	56
SUM	293074	44510	8820	0	0	0	0	2240	2974	21654	373272
α-mannosidase 2GHs											
GH38	18858	0	1138	0	0	0	0	0	1746	5024	26766
GH125	0	0	0	0	0	0	0	0	0	12	12
SUM	18858	0	1138	0	0	0	0	0	1746	5036	26778
α-rhamnosidase 3GHs											
GH78	0	0	0	0	0	0	0	0	0	156	156
GH106	55540	0	0	0	0	0	0	0	0	248	55788
SUM	55540	0	0	0	0	0	0	0	0	404	55944
galactosidase 11GHs											
GH1	8816	17598	13082	0	800	0	0	2428	1530	5190	49444
GH2	120070	27188	8820	0	0	0	0	1340	2974	8322	168714
GH4	11424	18946	0	8970	2504	924	0	778	1826	2492	47864
GH16	3800	0	0	0	0	0	0	0	0	680	4480
GH27	8546	0	0	0	0	0	0	0	0	0	8546
GH31	19042	13046	0	0	0	0	0	1046	2672	11828	47634
GH35	16778	0	0	0	0	0	0	0	0	34	16812
GH36	34026	14186	7154	0	0	0	0	946	0	5652	61964
GH42	0	12130	0	0	0	0	0	1026	0	2586	15742
GH53	23452	21720	0	0	0	0	0	1178	0	0	46350
GH95	37974	0	0	0	0	0	0	0	3302	3134	44410
SUM	283928	124814	29056	8970	3304	924	0	8742	12304	39918	511960
α-fucosidase 4GHs											
GH3	82982	0	6554	0	1860	0	0	1162	926	7160	100644
GH29	15164	8318	0	0	0	0	0	0	0	1798	25280
GH95	37974	0	0	0	0	0	0	0	3302	3134	44410
GH141	9140	0	0	0	0	0	0	0	0	0	9140
SUM	145260	8318	6554	0	1860	0	0	1162	4228	12092	179474
glycuronidase 7GHs											
GH4	11424	18946	0	8970	2504	924	0	778	1826	2492	47864
GH28	28346	0	0	0	0	0	0	0	0	124	28470
GH67	18406	11556	0	0	0	0	0	0	0	0	29962
GH88	9926	0		0	0	0		0	0	18	9944
GH105	48932	0	0	0	0	0	0	0	0	240	49172
GH115	12512	0	0	0	0	0	0	0	0	0	12512
GH138	0	0	0	0	0	0	0	0	2672	100	2772
SUM	129546	30502	0	8970	2504	924	0	778	4498	2974	180696
esterases 10CEs											
CE1	24396	0	11088	7664	5594	4304	0	2110	0	5518	60674
CE3	21556	5408	4894	3358	0	594	2330	910	0	62	39112
CE4	72584	36350	17676	3884	3520	5238	4584	2110	1144	5724	152814
CE6	0	0	0	0	0	0	0	0	0	978	978
CE7	14094	20876	11174	0	0	1182	0	620	0	2150	50096
CE8	15716	0	0	0	0	0	0	0	0	162	15878
CE9	28260	7934	0	4528	1660	2464	0	670	540	970	47026
CE10	40448	0	1218	3722	0	4980	0	1768	0	1470	53606
CE12	19080	6546	0	0	0	0	0	0	0	114	25740
SUM	236134	77114	46050	23156	10774	18762	6914	8188	1684	17148	445924

Abundance of specific genus on specific function R g-f = Ng-f/Nf

Functions

	Ruminiclos	Thermoan	Geobacillu	Bacillaceae	Aeribacillu	Symbioba	Desulfotor	Caldibacill	Clostridiur	others
Cellulose	355424	107998	23150	0	2660	3714	0	3590	2456	21768
	**68.25%**	**20.74%**	**4.45%**	**0.00%**	**0.51%**	**0.71%**	**0.00%**	**0.69%**	**0.47%**	**4.18%**
Hemicellulose	1186506	324052	82798	35588	15938	20864	6914	18992	24552	108952
	**65.01%**	**17.75%**	**4.54%**	**1.95%**	**0.87%**	**1.14%**	**0.38%**	**1.04%**	**1.35%**	**5.97%**
AA	25030	25228	34630	12296	7922	2150	4196	798	2026	10972
	**19.98%**	**20.14%**	**27.65%**	**9.82%**	**6.33%**	**1.72%**	**3.35%**	**0.64%**	**1.62%**	**8.76%**

Cellulose

	Ruminiclos	Thermoan	Geobacillu	Bacillaceae	Aeribacillu	Symbioba	Desulfotor	Caldibacill	Clostridiur	others
Endoglucanase	259512	90400	3514	1500	0	2536	984	0	0	6876
	**71.04%**	**24.75%**	**0.96%**	**0.41%**	**0.00%**	**0.69%**	**0.27%**	**0.00%**	**0.00%**	**1.88%**
exoglucanase	189840	45942	3514	1500	0	1470	0	0	0	4006
	**77.09%**	**18.65%**	**1.43%**	**0.61%**	**0.00%**	**0.60%**	**0.00%**	**0.00%**	**0.00%**	**1.63%**
β-glucosidase	258034	35496	19636	1500	2660	0	0	3590	2456	7790
	**77.92%**	**10.72%**	**5.93%**	**0.45%**	**0.80%**	**0.00%**	**0.00%**	**1.08%**	**0.74%**	**2.35%**

Hemicellulose

Main-chain

	Ruminiclos	Thermoan	Geobacillu	Bacillaceae	Aeribacillu	Symbioba	Desulfotor	Caldibacill	Clostridiur	others
endo-β-1,4-xylanase	189302	62364	0	1500	0	0	0	0	2826	19518
	**68.71%**	**22.64%**	**0.00%**	**0.54%**	**0.00%**	**0.00%**	**0.00%**	**0.00%**	**1.03%**	**7.08%**
β-xylosidase	262300	67300	19636	0	2660	0	0	4490	4850	35074
	**66.19%**	**16.98%**	**4.95%**	**0.00%**	**0.67%**	**0.00%**	**0.00%**	**1.13%**	**1.22%**	**8.85%**
β-mannosidase	190354	62684	21902	1500	800	0	0	3768	4504	17518
	**62.82%**	**20.69%**	**7.23%**	**0.50%**	**0.26%**	**0.00%**	**0.00%**	**1.24%**	**1.49%**	**5.78%**

debranching

	Ruminiclos	Thermoan	Geobacillu	Bacillaceae	Aeribacillu	Symbioba	Desulfotor	Caldibacill	Clostridiur	others
arabinofuranosidases	293074	44510	8820	0	0	0	0	2240	2974	21654
	**78.51%**	**11.92%**	**2.36%**	**0.00%**	**0.00%**	**0.00%**	**0.00%**	**0.60%**	**0.80%**	**5.80%**
α-mannosidase	18858	0	1138	0	0	0	0	0	1746	5036
	**70.42%**	**0.00%**	**4.25%**	**0.00%**	**0.00%**	**0.00%**	**0.00%**	**0.00%**	**6.52%**	**18.81%**
α-rhamnosidase	55540	0	0	0	0	0	0	0	0	404
	**99.28%**	**0.00%**	**0.00%**	**0.00%**	**0.00%**	**0.00%**	**0.00%**	**0.00%**	**0.00%**	**0.72%**
galactosidase	283928	124814	29056	8970	3304	924	0	8742	12304	39918
	**55.46%**	**24.38%**	**5.68%**	**1.75%**	**0.65%**	**0.18%**	**0.00%**	**1.71%**	**2.40%**	**7.80%**
α-fucosidase	145260	8318	6554	0	1860	0	0	1162	4228	12092
	**80.94%**	**4.63%**	**3.65%**	**0.00%**	**1.04%**	**0.00%**	**0.00%**	**0.65%**	**2.36%**	**6.74%**
glycuronidase	129546	30502	0	8970	2504	924	0	778	4498	2974
	**71.69%**	**16.88%**	**0.00%**	**4.96%**	**1.39%**	**0.51%**	**0.00%**	**0.43%**	**2.49%**	**1.65%**
esterases	236134	77114	46050	23156	10774	18762	6914	8188	1684	17148
	**52.95%**	**17.29%**	**10.33%**	**5.19%**	**2.42%**	**4.21%**	**1.55%**	**1.84%**	**0.38%**	**3.85%**

	Ruminiclos	Thermoan	Geobacillu	Bacillaceae	Aeribacillu	Symbioba	Desulfotor	Caldibacill	Clostridiur	others
**Hemi_debran**	992872	239124	82798	34088	15938	20864	6914	18992	19332	82126
	**65.62%**	**15.80%**	**5.47%**	**2.25%**	**1.05%**	**1.38%**	**0.46%**	**1.26%**	**1.28%**	**5.43%**
**Hemi_mainchain**	546836	147036	28456	3462	2660	1178	0	5830	10650	57584
	**68.04%**	**18.30%**	**3.54%**	**0.43%**	**0.33%**	**0.15%**	**0.00%**	**0.73%**	**1.33%**	**7.16%**

Summary

	Ruminiclos	Thermoan	Geobacillu	Bacillaceae	Aeribacillu	Symbioba	Desulfotor	Caldibacill	Clostridiur	others
Cellulose	0.68251	0.207385	0.044454	0	0.005108	0.007132	0	0.006894	0.004716	0.0418
Hemicellulose	0.650085	0.177548	0.045365	0.019499	0.008732	0.011431	0.003788	0.010406	0.013452	0.059695
AA	0.199844	0.201424	0.276491	0.098173	0.063251	0.017166	0.033502	0.006371	0.016176	0.087602
Endoglucanase	0.710365	0.247453	0.009619	0.004106	0	0.006942	0.002694	0	0	0.018822
exoglucanase	0.770855	0.18655	0.014269	0.006091	0	0.005969	0	0	0	0.016267
β-glucosidase	0.779178	0.107186	0.059294	0.00453	0.008032	0	0	0.010841	0.007416	0.023523
endo-β-1,4-xylanase	0.687097	0.226358	0	0.005444	0	0	0	0	0.010257	0.070843
β-xylosidase	0.661856	0.169817	0.049547	0	0.006712	0	0	0.01133	0.012238	0.088501
β-mannosidase	0.628169	0.206857	0.072277	0.00495	0.00264	0	0	0.012434	0.014863	0.057809
arabinofuranosidases	0.785149	0.119243	0.023629	0	0	0	0	0.006001	0.007967	0.058011
α-mannosidase	0.704235	0	0.042498	0	0	0	0	0	0.065203	0.188065
α-rhamnosidase	0.992778	0	0	0	0	0	0	0	0	0.007222
galactosidase	0.55459	0.243796	0.056754	0.017521	0.006454	0.001805	0	0.017076	0.024033	0.077971
α-fucosidase	0.809365	0.046347	0.036518	0	0.010364	0	0	0.006474	0.023558	0.067375
glycuronidase	0.716928	0.168803	0	0.049641	0.013858	0.005114	0	0.004306	0.024893	0.016459
esterases	0.529539	0.172931	0.103269	0.051928	0.024161	0.042074	0.015505	0.018362	0.003776	0.038455

Taxon abundance Rg

	Ruminiclos	Thermoan	Geobacillu	Bacillaceae	Aeribacillu	Symbioba	Desulfotor	Caldibacill	Clostridiur	others
Taxon abundance	42.85%	18.41%	10.44%	7.48%	2.65%	2.47%	2.33%	1.56%	1.26%	10.55%

Ccontribution coefficient CC=R g-f/Rg

	Ruminiclos	Thermoan	Geobacillu	Bacillaceae	Aeribacillu	Symbioba	Desulfotor	Caldibacill	Clostridiur	others
Cellulose	1.592789	1.126482	0.425807	0	0.192752	0.28874	0	0.441908	0.3743	0.396213
Hemicellulose	1.517117	0.964408	0.43453	0.260677	0.329525	0.462808	0.162582	0.667031	1.067619	0.565826
AA	0.466379	1.094103	2.648385	1.312476	2.386812	0.694977	1.437834	0.40842	1.283802	0.830353
Endoglucanase	1.657795	1.344122	0.092135	0.054893	0	0.281045	0.115601	0	0	0.178405
exoglucanase	1.798961	1.013307	0.136674	0.081428	0	0.24166	0	0	0	0.154185
β-glucosidase	1.818384	0.582217	0.567953	0.060555	0.303107	0	0	0.694911	0.588596	0.222969
endo-β-1,4-xylanase	1.603493	1.22954	0	0.072787	0	0	0	0	0.814075	0.671499
β-xylosidase	1.544587	0.922415	0.474589	0	0.25328	0	0	0.726251	0.971261	0.838876
β-mannosidase	1.465972	1.123614	0.692305	0.066177	0.099623	0	0	0.797078	1.17962	0.547957
arabinofuranosidases	1.832319	0.647707	0.22633	0	0	0	0	0.384679	0.632332	0.54987
α-mannosidase	1.643488	0	0.407065	0	0	0	0	0	5.174824	1.782605
α-rhamnosidase	2.316869	0	0	0	0	0	0	0	0	0.06845
galactosidase	1.29426	1.324261	0.543625	0.234237	0.243533	0.07307	0	1.094587	1.907391	0.739061
α-fucosidase	1.888833	0.251747	0.349788	0	0.39108	0	0	0.41503	1.869661	0.638622
glycuronidase	1.673111	0.916908	0	0.663655	0.522926	0.207027	0	0.275998	1.975606	0.156006
esterases	1.235796	0.939331	0.989164	0.694226	0.911738	1.703418	0.665446	1.177043	0.299717	0.364502
